# Dynamism and evolvability in nucleic acid enzymes

**DOI:** 10.1038/s41467-026-76473-9

**Published:** 2026-08-14

**Authors:** Erica M. Lee, John C. Chaput

**Affiliations:** 1 Department of Pharmaceutical Sciences, University of California, Irvine, CA 92697 USA; 2 Department of Chemistry, University of California, Irvine, CA 92697 USA; 3 Department of Molecular Biology and Biochemistry, University of California, Irvine, CA 92697 USA; 4 Department of Chemical and Biomolecular Engineering, University of California, Irvine, CA 92697 USA

**Keywords:** Synthetic biology, Nucleic acids, Biocatalysis

## Abstract

Protein dynamism and evolvability are key parameters linking enzyme flexibility to adaptive potential, yet how these concepts apply to nucleic acid enzymes remains largely unexplored. Here, we propose that threose nucleic acid (TNA), a genetic polymer that is more conformationally restricted than DNA, may be evolutionarily constrained by its preorganized backbone. RNA-cleavage profiles comparing the well-known 10-23 DNA enzyme (DNAzyme) with two in vitro selected TNA enzymes (threozymes) reveal striking differences in their temperature dependence. While the DNAzyme catalyzed reaction is optimal at 37 °C and weakly active at 50 °C, threozymes display the opposite trend, suggesting that TNA catalysis must overcome a higher free-energy barrier than DNA catalysis. Consistent with this view, the reaction becomes less temperature dependent for threozymes that operate with more flexible active sites. Together, these findings highlight the importance of backbone structure as a critical parameter for evolvability with implications for RNA world models and biomedical applications.

## Abstract (web summary)

Dynamism and evolvability link enzyme flexibility to adaptive potential, yet how these concepts apply to nucleic acid enzymes is largely unexplored. Here the authors compare the evolution of threose nucleic acids (TNA) and DNA-based enzymes, and show the rigid TNA backbone limits adaptability.

## Introduction

A defining feature of protein enzymes is their capacity to explore conformational landscapes. Rather than acting as rigid scaffolds, most enzymes populate ensembles of interconverting states that collectively shape their catalytic efficiency and evolutionary potential through active sites that position reactive groups to achieve a stable transition state^1^. Mutations can alter these ensembles by stabilizing alternative conformations that allow new enzyme functions to emerge from existing folds^2–4^. This coupling between conformational dynamics and function led to the view that enzyme evolution proceeds through conformational landscapes where structural plasticity enables proteins to access new catalytic states, thereby making dynamism an important determinant of evolvability^5^.

The rise of diverse classes of genetic polymers, so-called xeno-nucleic acids or XNAs, provides an opportunity to ask whether the principles linking dynamics and evolution in proteins also extend to nucleic acid enzymes^6,7^. Unlike proteins, whose folding behavior is largely driven by side chain packing in a hydrophobic core^8,9^, nucleic acid structure is dictated by the composition and geometry of the sugar-phosphate backbone^10,11^. Even subtle chemical changes can profoundly alter the folding stability, helical geometry, and base-pairing preferences of a genetic system^12^. As an example, deoxyxylo nucleic acid (dXyloNA), an epimer of DNA with inverted stereochemistry at the 3’ hydroxy, forms a left-handed duplex with helical parameters that are distinct from either DNA or RNA^13^. Such observations suggest that backbone chemistry may strongly control the folding of a genetic system, enabling some polymers to access conformationally diverse and functionally productive landscapes while restricting others to narrower regions of structure-function space. From this perspective, nucleic acid backbones could represent a critical determinant of evolvability, dictating the extent to which sequences can be converted into folds and folds into functions.

**Fig. 1:**
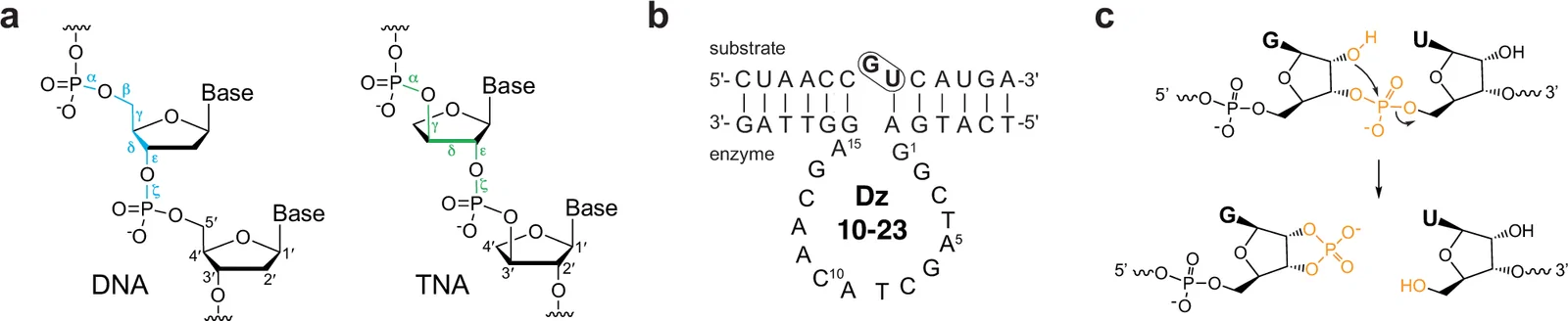
Nucleic acid catalysis. **a** Chemical structures, backbone torsion angles, and sugar atom numbering of DNA (blue) and TNA (green). **b** Dz 10-23 in complex with an RNA substrate. The G-U cleavage site is shown in bold inside a circle. **c** Mechanism of magnesium-dependent RNA cleavage.

Among the XNA systems capable of Darwinian evolution, 3’,2’-α-L-threose nucleic acid (TNA, Fig. 1a) has received considerable attention from the origins community as a possible progenitor of RNA and from the synthetic biology community as an alternative genetic polymer with potential applications in biotechnology and medicine^14–18^. Despite having a shorter backbone based on a four-carbon threose sugar, TNA forms stable antiparallel Watson-Crick duplexes with itself as well as with complementary strands of DNA and RNA^19^, and it can undergo Darwinian evolution using engineered polymerases^20^. These advances have enabled the isolation of aptamers and catalysts from large, unbiased pools of random TNA sequences^21–25^. However, structural studies reveal that TNA is more conformationally restricted than DNA and RNA^26^, raising the possibility that backbone geometry may influence the evolutionary landscape accessible to TNA enzymes.

Here, we examine how backbone chemistry shapes nucleic acid catalysis by comparing RNA cleavage activity mediated by the well-characterized 10-23 DNA enzyme (DNAzyme, Dz)^27^ with that of two independently evolved TNA enzymes (threozymes, Tz) that act on the same substrate. Whereas the DNAzyme displays maximal activity near physiological temperature, the threozymes become increasingly active at elevated temperatures. These opposing temperature profiles suggest that TNA-mediated catalysis requires overcoming a larger free-energy barrier, consistent with a catalytic profile constrained by a more rigid backbone architecture. Notably, the temperature dependence narrows for TNA catalysts that operate with less defined active sites, indicating that increased structural flexibility alleviates this constraint. Together, these results show that backbone chemistry can shape the energetic landscape of nucleic acid catalysts and suggest that the architecture of a genetic polymer may place fundamental limits on its catalytic evolvability.

## Results

Recognizing that TNA maintains a backbone repeat unit that is both shorter and more physically constrained than DNA and RNA (Fig. 1a)^19^, we asked whether these properties might limit the ability of TNA to fold into structures with defined functional properties. We were particularly interested in how RNA-cleaving catalysts, one of the most accessible classes of nucleic acid enzymes^28–30^, would compare when evolved in the context of a TNA backbone. Would in vitro selection produce a rich distribution of threozymes with varied catalytic activities, as is commonly seen for DNA enzymes^31^, or would the conformational preorganization of the TNA backbone compress the evolutionary landscape into a flatter horizon with only a limited set of functional solutions?

To address this question, we performed parallel in vitro selection experiments designed to isolate RNA-cleaving catalysts from pools of unbiased random-sequence TNA molecules. Guided by prior studies^32–34^, we targeted a segment of the Kirsten rat sarcoma (KRAS) oncogene, one of the most frequently mutated drivers in human cancers^35^. The clinically relevant G12V variant, long considered difficult to target therapeutically^36^, arises from a G→U mutation at position 2 of codon 12, producing a glycine-to-valine substitution in the translated protein sequence. This single-nucleotide difference creates a unique G-U junction in the mutant transcript that is absent from the wild-type sequence, enabling allele-specific targeting by RNA-cleaving DNAzymes^37^.

Prior structural and biochemical studies have shown that the classic 10-23 DNAzyme cleaves such junctions via a general acid-base mechanism (Fig. 1b, c) whereby dG14 within the catalytic loop abstracts a proton from the O2’ atom of the scissile rG residue, while a hydrated magnesium ion simultaneously donates a proton to the O5’ atom of the adjacent U^-1^ residue^38,39^. The resulting O2’ oxyanion then performs an in-line nucleophilic attack on the adjacent phosphodiester bond, yielding an upstream fragment bearing a cyclic 2’,3’-monophosphate and a downstream product with a 5’ hydroxyl terminus. Whether TNA can be evolved to catalyze this reaction using a similarly constrained active site—one in which the substrate remains fully base-paired up to the exocyclic guanine at the G-U cleavage site—remains unknown, raising the broader question of whether a conformationally restricted backbone can support the precise positioning and chemical cooperativity required for efficient phosphodiester cleavage.

**Fig. 2:**
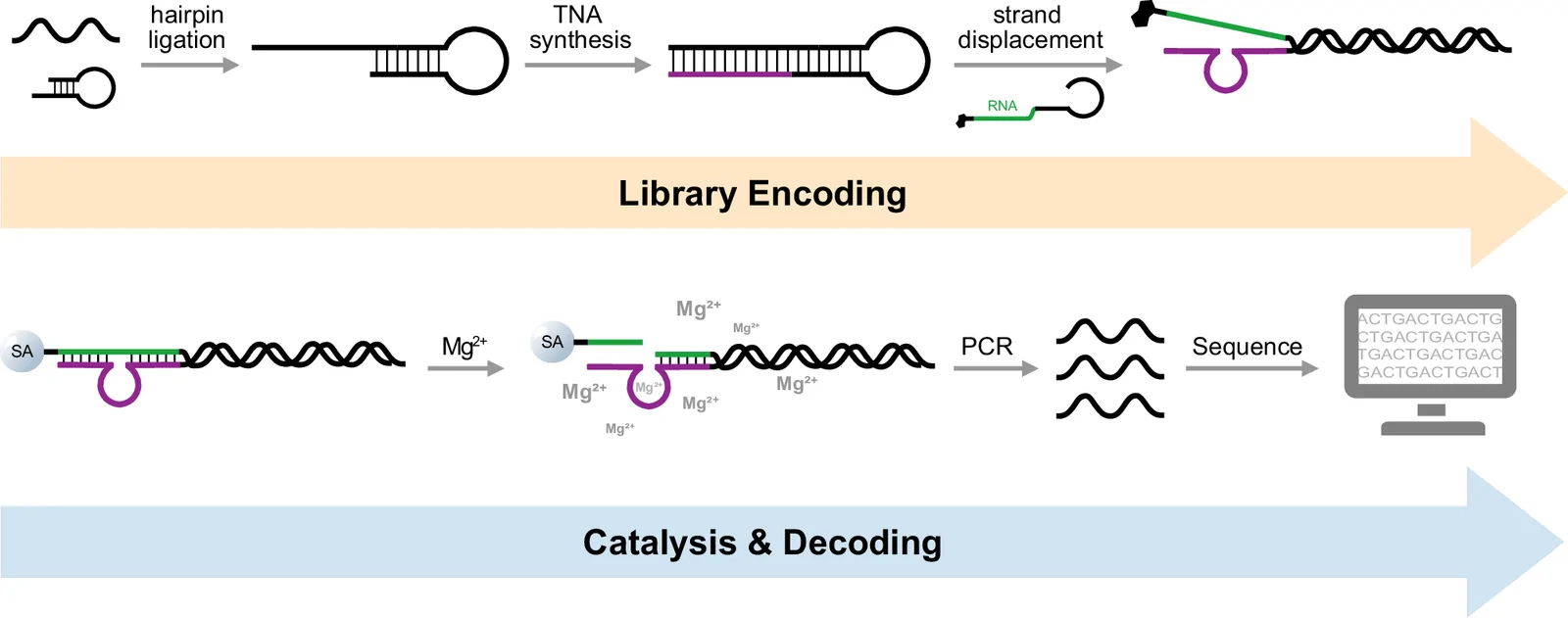
TNA libraries prepared and screened by DNA display. A pool of single-stranded DNA (black) ligated to a self-priming stem-loop is extended with TNA (purple). Strand displacement initiated by a second primer extension reaction generates the genotype-phenotype linkage. The RNA substrate (green) is introduced via conjugation to the strand-displacement primer. The libraries are immobilized on streptavidin-coated (SA) magnetic beads, and functional TNA molecules cleave themselves off the bead in the presence of magnesium ions. The recovered DNA is PCR-amplified and used to generate another round of selection or to be sequenced.

The selection was performed using a DNA display strategy in which each TNA molecule was physically linked to its encoding double-stranded (ds) DNA template^40^. As illustrated in Fig. 2, a 5’-phosphorylated DNA stem-loop structure was ligated onto the 3’ end of two different chemically synthesized DNA libraries carrying random regions of either 15 or 20 consecutive nucleotides (nt) (Supplementary Table 1). The pools of self-priming templates were then extended with chemically synthesized TNA triphosphates (tNTPs)^41,42^ using the recently evolved 10-92 TNA polymerase^43^. The resulting TNA-DNA hairpin duplex was denatured in a strand displacement reaction using Bst DNA polymerase to extend a DNA primer annealed to the loop region of the hairpin with DNA (Supplementary Fig. 1). The products of this reaction were pools of single-stranded (ss) TNA molecules that had become physically linked to their encoding dsDNA. This design placed the TNA molecules across from the RNA substrate, introduced via the strand displacement primer.

Iterative cycles of in vitro selection and amplification were performed using libraries that oversampled the sequence space defined by 15- and 20 random nucleotide positions (N_15_ and N_20_), corresponding to 60,200 and 60 copies per sequence, respectively. This library length was chosen to be consistent with Dz 10-23, which has a catalytic domain comprised of 15 unpaired nucleotides^27^, and a slightly longer length to account for the reduced flexibility of TNA^26^. For each round of selection, the DNA display libraries were applied to a streptavidin-coated solid support and washed with selection buffer to remove the unbound molecules (Fig. 2). Functional TNA catalysts were then eluted by incubating the resin with selection buffer supplemented with 20 mM MgCl_2_ at pH 7.5 and 37 °C. Under these conditions, catalytically active sequences cleave a predefined G-U phosphodiester linkage in the RNA substrate (Fig. 1b), releasing the 3’ cleavage product into the eluate. TNA sequences recovered from the eluate were isolated and amplified by the polymerase chain reaction (PCR) using DNA primers designed to recognize fixed sequences flanking the random region in the encoding DNA tag. The amplified DNA was made single stranded and used to generate new libraries of TNA molecules for subsequent rounds of selection.

As the selections progressed, the stringency was increased by progressively reducing both the incubation time and MgCl_2_ concentration. The divalent metal ion cofactor was decreased from 20 to 0.5 mM MgCl_2_, while the reaction time was shortened from 19 hours to 15 minutes. After 8 rounds of selection, DNA amplicons isolated from rounds 4, 6, and 8 of both selections were analyzed by next-generation sequencing (NGS) using an Illumina MiSeq platform. The six most enriched sequences from each selection (Supplementary Table 2) were synthesized in a 7 + 7 binding arm configuration by solid phase synthesis (SPS) using TNA phosphoramidites prepared by chemical synthesis^41^. The TNA sequences are largely unrelated to one another (Hamming distances: N15 members, 6-13; N20 members, 12-15), indicating that each molecule emerged as an independent solution to the problem of how TNA could catalyze the cleavage of an RNA substrate.

**Fig. 3:**
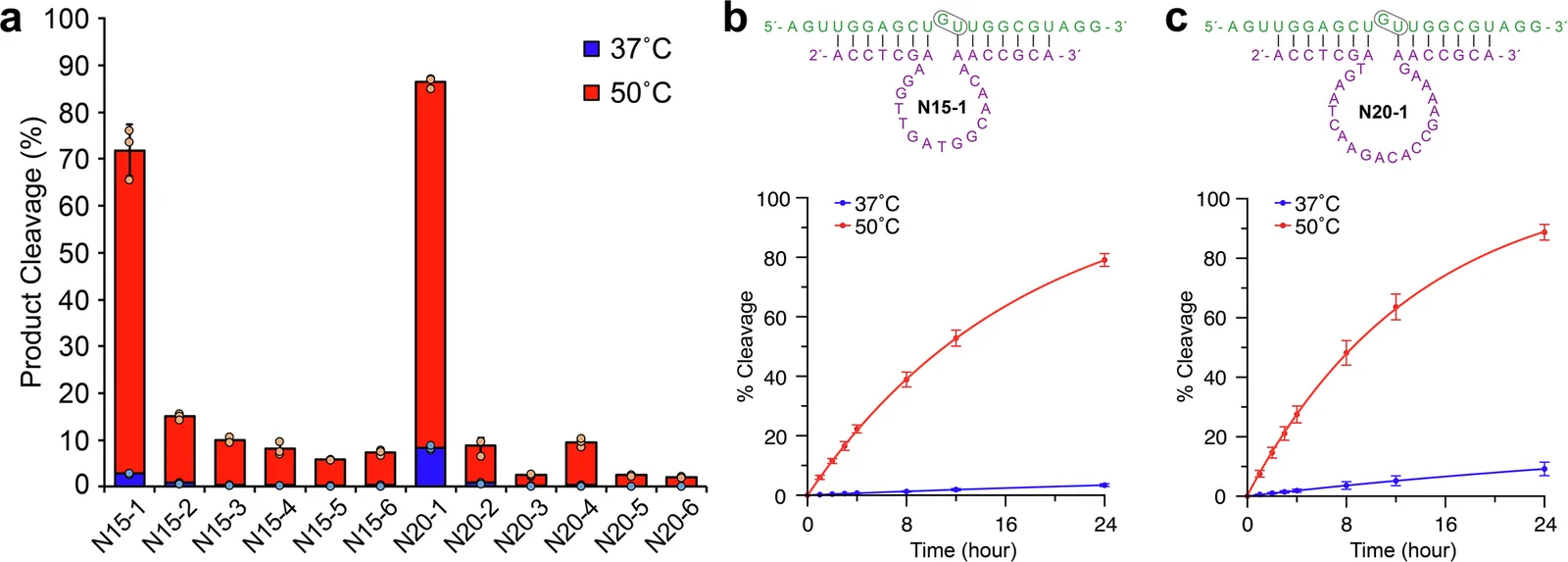
Threozyme-mediated RNA cleavage at moderate and elevated temperatures. **a** Activity screen. Single endpoint detection analysis of RNA cleavage mediated by the six most enriched threozymes isolated from the N15 and N20 libraries. Bar graphs denote average RNA cleavage activity after 24 hours of incubation at 37 °C (blue) and 50 °C (red). **b, c** Kinetic curves. Reaction profiles showing RNA cleavage of the most active variants isolated from the N15 (N15−1, panel b) and N20 (N20-1, panel c) libraries at 37°C (blue) and 50 °C (red). The enzyme-substrate complex is provided above the plot. The G-U cleavage is the site denoted with a circle. Data presented as the mean ± standard deviation (*n* = 3 independent experiments). All reactions were performed in 50 mM Tris (pH 7.5), 10 mM NaCl, 140 mM KCl, and 20 mM MgCl_2_ with 250 nM substrate and 500 nM enzyme. Source data for this figure is available in the Source Data file.

Each chemically synthesized threozyme was evaluated for its ability to cleave a 5’ labeled RNA substrate under bimolecular, in-trans cleavage conditions, where the enzyme and substrate were provided as separate strands (Supplementary Tables 2–3). Under single turnover conditions (20 mM MgCl_2_, 37 °C, 24 hours), only the most enriched sequence from each selection (N15-1 and N20-1) exhibits detectable RNA cleavage activity, and only at trace levels (Fig. 3a, Supplementary Fig. 2). This result suggests that threozyme activity may be strongly influenced by temperature. One possible explanation is that the shorter, more conformationally constrained TNA backbone limits access to catalytically productive conformations at lower temperatures, thereby requiring thermal activation to form an active complex. However, other factors, including substrate binding, metal ion coordination, or temperature-dependent folding equilibria, could also contribute to the observed behavior. Consistent with a strong temperature dependence, all six threozymes derived from the N15 library and three of the six threozymes from the N20 library show increases in catalytic activity when the assay is repeated at 50 °C (Fig. 3a, Supplementary Fig. 2). Notably, the top performing sequences (N15-1 and N20-1) exhibit a dramatic enhancement in reactivity, with substrate cleavage increasing from ~3-5% at 37 °C to >50% at 50 °C.

A time-course analysis of the top-performing enzymes illustrates the temperature-dependent behavior (Fig. 3b, c, Supplementary Fig. 3) of the selected threozymes. Both N15-1 and N20-1 exhibit negligible activity at 37 °C, whereas the same reactions performed at 50 °C show rapid and sustained product formation. The N15-1 catalyzed reaction reaches ~80% product cleavage within 24 h, while N20-1 achieves slightly higher activity ( ~ 90%) with faster initial rates. The kinetic profiles are consistent with a temperature-activated catalytic mechanism, where elevated temperature enhances the conformational dynamics required for phosphodiester bond cleavage.

To confirm that RNA cleavage occurs at the intended site, we compared the migration pattern of threozyme-mediated RNA cleavage products to an RNase T1 digestion ladder, which specifically cleaves RNA after guanosine residues, using denaturing PAGE. Both N15-1 and N20-1 generate an RNA product that co-migrates with the expected fragment corresponding to cleavage of the RNA substrate at the desired G-U dinucleotide junction (Supplementary Fig. 4). We next examined the magnesium dependence of the reaction. Increasing the Mg^2+^ concentration from 0 to 20 mM resulted in corresponding increases in RNA cleavage for both N15-1 and N20-1 (Supplementary Fig. 5), with no detectable activity observed in the absence Mg^2+^ ions (0 mM). These results indicate that catalysis proceeds through a magnesium-dependent cleavage mechanism, consistent with divalent metal ion-catalyzed phosphodiester cleavage. Finally, we evaluated the resistance of threozymes to biological nucleases. Preincubation of the enzyme-substrate complexes with Turbo DNase or human RNase H1 had no measurable effect on the catalytic activity of either N15-1 or N20-1 (Supplementary Figs. 6–7), indicating that both enzymes are refractory to biological nucleases. In contrast, Dz 10-23, used as a general model for DNAzyme-mediated RNA cleavage, lost all activity following DNase treatment and exhibited altered cleavage patterns in the presence of RNase H1 (Supplementary Figs. 6–7), consistent with its susceptibility to RNase H1-mediated strand cleavage^44^. Together, these findings reinforce the intrinsic biostability of TNA and highlight its promise for applications in biological environments where resistance to nuclease degradation is critical for function^45^.

**Fig. 4:**
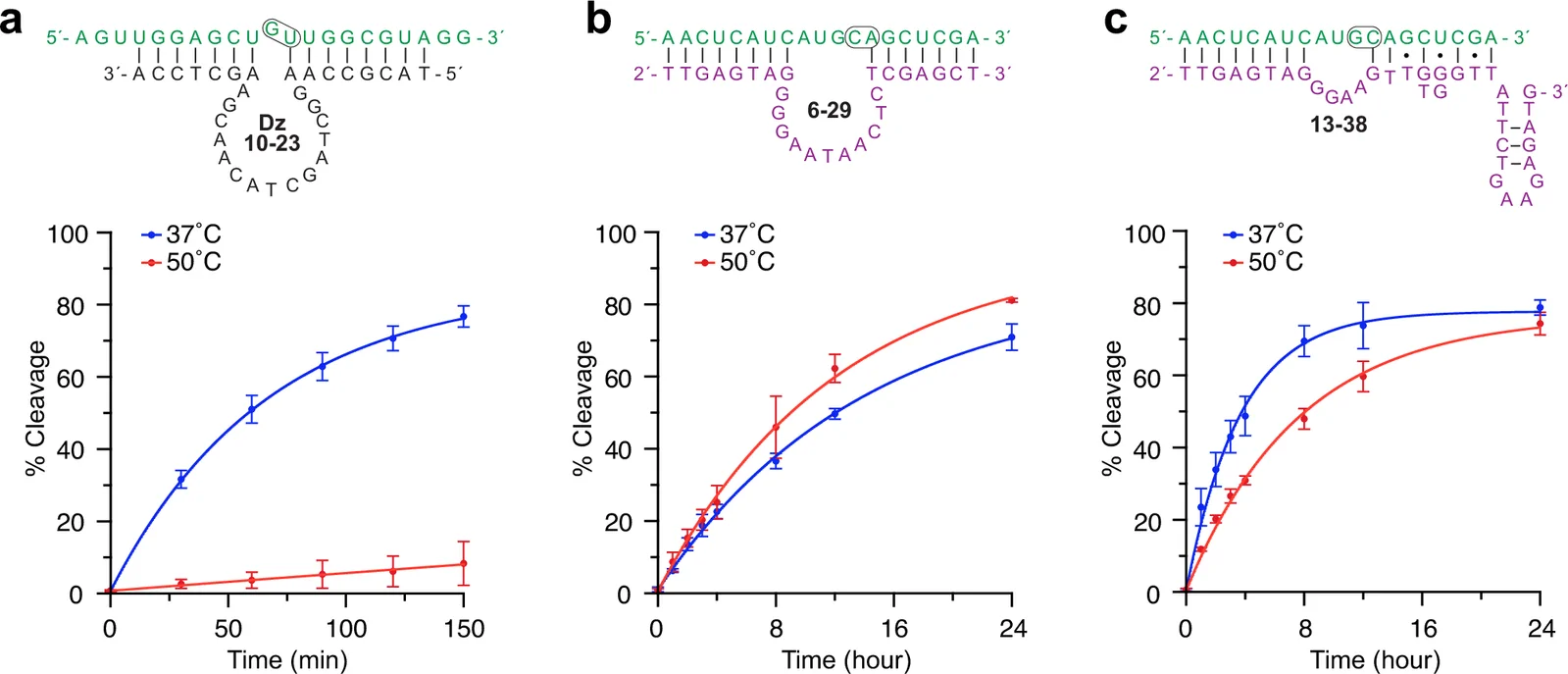
Comparative reaction profiles performed at moderate and elevated temperatures. **a** Kinetic curves of Dz 10-23. **b, c** Kinetic curves of Tz’s 6-29 and 13–38. Reaction profiles show RNA cleavage at 37 °C (blue) and 50 °C (red). The enzyme-substrate complex is provided above the plot. The cleavage site is denoted with a circle. Data presented as the mean ± standard deviation (*n *= 3 independent experiments). All reactions were performed in 50 mM Tris (pH 7.5), 10 mM NaCl, and 140 mM KCl. Dz catalyzed reactions contained 2 mM MgCl_2_ with 250 nM substrate and 25 nM enzyme, while the Tz catalyzed reactions contained 20 mM MgCl_2_ with 250 nM substrate and 500 nM enzyme. Source data for this figure is available in the Source Data file.

To evaluate how backbone chemistry might influence catalytic behavior, we evaluated the RNA cleavage activity of Dz 10-23 at physiological and elevated temperature^27^. To obtain measurable reaction kinetics that could be compared with those of the threozymes, Dz 10-23 reactions were performed under deliberately attenuated conditions using 2 mM Mg^2+^ rather than 20 mM Mg^2+^. Reactions were evaluated under multiple and single turnover conditions. Under multiple turnover conditions, Dz 10-23 exhibited robust catalytic activity at 37 °C, achieving ~80% substrate cleavage in 150 minutes. In contrast, catalytic activity was markedly reduced at 50 °C, with cleavage yields remaining below ~15%, even at extended reaction times (Fig. 4a, Supplementary Fig. 8). A similar trend was observed under single turnover conditions, where Dz 10-23 achieved ~90% substrate cleavage within 10 minutes at 37° C but only ~15% at 50 °C (Supplementary Fig. 9). These observations indicate that Dz 10–23 activity decreases substantially at elevated temperature, which is consistent with previous reports describing the temperature-dependence of other RNA-cleaving DNAzymes^46,47^.

Thermal denaturation profiles performed in the reaction buffer using a 2’-deoxyguanosine (dG) substitution at the cleavage junction indicate that while substrate dissociation could account for loss of activity at elevated temperature, the Tm value of Dz 10-23 is strikingly similar to that of Tz N20-1, which remains optimal at 50 °C (Supplementary Fig. 10). It is worth noting that the dG substitution at the substrate cleavage site could lower the melting temperature of the enzyme-substrate complex by disrupting key interactions involving coordination of the catalytic metal-ion to the 2’ hydroxyl group. Indeed, NMR analysis of Dz 10-23 reveals a highly dynamic loop structure coordinated to three divalent metal ions^39,48^.

Next, we extended these studies to include chemically synthesized threozymes (Tz’s 6-29 and 13-38) previously evolved to cleave RNA with a more flexible active site (Fig. 4b, c)^24^. Notably, these enzymes display a reduced temperature dependence, maintaining high catalytic activity at both 37 °C and 50 °C (Supplementary Fig. 11). The activity profiles of the enzymes evaluated in this study across a range of temperatures (Supplementary Fig. 12) reveal a fundamental difference in the catalytic behavior of nucleic acid enzymes that operate with distinct active site architectures. Enzymes with more conformationally constrained active sites appear to function optimally within a narrow temperature range, whereas those with more flexible active sites exhibit broader thermal tolerance, retaining catalytic activity across a wider temperature range.

To further evaluate the influence of temperature on catalytic activity, apparent rate constants (k_obs_) measured at 37 °C and 50 °C were used to estimate activation energies (E_a_) for each catalyst (Supplementary Table 4). The two enzymes isolated from the random sequence libraries, Tz N15-1 and Tz N20-1, exhibit a strong positive temperature dependence, with k_obs_ increasing from 0.02 h^-1^ at 37 °C to 0.06-0.07 h^-1^ at 50 °C, corresponding to apparent activation energies of 77.5 ± 7.2 and 79.4 ± 2.7 kJ/mol, respectively. In contrast, previously reported threozymes with more flexible active sites display a reduced or reversed temperature dependence. Tz 6-29 showed only a modest increase in activity with temperature (0.07 to 0.08 h^-1^), yielding a near zero activation energy (9.1 ± 5.3 kJ/mol), while Tz 13-38 exhibited a 2-fold decrease in activity at elevated temperature (0.27 to 0.13 h^-1^), corresponding to an apparent negative activation energy of -47.0 ± 5.7 kJ/mol. A similar but more pronounced trend was observed for Dz 10-23, which showed robust activity at 37 °C (21.7 h^-1^) but a greater than 10-fold reduction at 50 °C (1.8 h^-1^), yielding an apparent activation energy of -158.2 ± 14.4 kJ/mol. Collectively, these results demonstrate that the newly identified threozymes exhibit a fundamentally different temperature dependence from both Dz 10-23 and previously characterized threozymes, with catalytic activity increasing rather than decreasing with elevated temperature. This behavior is consistent with a model in which catalysis by the newly selected threozymes requires thermal activation to access productive conformational states.

## Discussion

A central principle of enzymology is that catalytic function emerges from the ability of molecules to access ensembles of interconverting conformations^1^. In proteins, this dynamic plasticity provides a foundation for evolvability, enabling mutations to reshape conformational landscapes and reveal new catalytic states^2–4^. Our results suggest that an analogous principle applies to nucleic acid enzymes, but with an important distinction. While protein dynamics are encoded primarily through side-chain interactions^8,9^, nucleic acid dynamics are fundamentally constrained more directly by the chemical structure of their sugar-phosphate backbone^10^. In this context, TNA provides an informative model system for examining how backbone geometry governs the relationship between molecular motion and catalytic function.

The opposing temperature-dependent activity profiles observed for similarly sized DNA and TNA catalysts acting on the same RNA substrate reveal differences in how nucleic acid enzymes access productive transition states. Dz 10-23 exhibits maximal activity near physiological temperature but is strongly attenuated at elevated temperature, whereas Tz’s N15-1 and N20-1 display only minimal activity at 37 °C and become highly active at 50 °C. Based on this data and their associated melting curves, we suggest a model in which TNA catalysis depends on conformational states that are only sparsely populated at low temperature. Raising the temperature then provides the thermal energy necessary to overcome this barrier, allowing threozymes to access conformations that are more compatible with the geometric constraints of site-specific RNA hydrolysis.

This interpretation is consistent with the structural properties of TNA, which maintains a backbone repeat unit that is one bond shorter than that of DNA or RNA; thus, each nucleotide has fewer degrees of conformational freedom. Although this feature may stabilize certain folded states, it may also restrict the ability of TNA to sample alternative conformations required for catalysis^49^. From this perspective, the weak activity of in vitro selected threozymes at 37 °C reflects an entropic bottleneck in which the enzymes remain kinetically trapped in unproductive conformations. Elevating the temperature partially relieves this constraint by increasing the probability of forming a productive active site through conformational sampling.

Our comparison with previously reported threozymes that possess more flexible active sites reinforces this model. Unlike N15-1 and N20-1, these catalysts remain active across a broader range of temperatures, suggesting that local relaxation of the active site can partially offset intrinsic constraints imposed by the TNA backbone. Such findings point to a continuum in which catalytic efficiency is shaped by the balance between backbone structure and access to dynamic catalytic states. Highly constrained architectures may favor precise positioning but at the cost of reduced conformational flexibility, whereas less constrained systems may sample a broader range of states, including those compatible with catalysis. This interpretation is consistent with the NMR structure of 10-23 showing extensive sampling of conformational space (Supplementary Fig. 13)^39^. As expected, there is likely to be a limit to this balance, as some genetic systems may prove too rigid or too flexible to support efficient catalysis, but systematically testing this idea across a range of XNA systems would offer clues into how different classes of nucleic acid systems fold and function^50^. Additionally, base modifications could influence the temperature-dependent activity of nucleic acid enzymes by altering the reaction mechanism^51–53^.

These findings have important implications for the evolvability of nucleic acid enzymes. Although in vitro selection has now yielded multiple RNA-cleaving threozymes, demonstrating that functional solutions do exist in TNA sequence space, their comparatively low activity and pronounced temperature dependence suggests that the accessible fitness landscape is narrower than that observed for comparable DNA enzymes^28–30^. Such compression could help explain both the limited diversity of catalytic motifs recovered in our selections and the failure to isolate highly active threozymes under standard conditions. More broadly, our results support the idea that backbone chemistry does not simply modulate the stability of the polymer but also defines the dynamic landscape from which catalytic function can emerge. Such factors could influence the choice of genetic polymers evaluated for practical applications as well as various scenarios used to describe the emergence of the RNA world^14–18^.

In summary, our study establishes a direct link between backbone geometry and catalytic activity in nucleic acid enzymes. By showing that TNA-mediated catalysis is governed by the temperature-dependent activation of conformational states, we provide experimental evidence that backbone chemistry can impose fundamental limits on evolvability, which could have important implications for RNA world models and biomedical applications.

## Methods

### Materials

TNA triphosphates and phosphoramidites were synthesized in-house^41,42^. DNA phosphoramidite monomers were purchased from Glen Research (Sterling, VA). DNA triphosphates were purchased from Thermo Fisher Scientific (Waltham, MA). DNA/RNA oligonucleotides and DNA libraries were purchased from Integrated DNA Technologies (Coralville, Iowa). Glen-Pak DNA Purification Cartridges were purchased from Glen Research (Sterling, VA). T7 DNA ligase and 10-92 TNA polymerase were expressed and purified from E. coli^54^. T4 DNA ligase buffer, ThermoPol buffer, and Bst 3.0 DNA polymerase were purchased from New England Biolabs (Ipswich, MA). PCRBIO HiFi polymerase and PCRBIO HIFI buffer were purchased from PCR Biosystems (London, UK). Phenol:chloroform:isoamyl alcohol (25:24:1 v/v, saturated with 10 mM Tris, pH 8.0, 1 mM EDTA) was purchased from Sigma-Aldrich (St. Louis, MO). Amicon ultra-centrifugal filters were purchased from Millipore Sigma (Burlington, MO). DNA Clean and Concentrator-5 was purchased from Zymo Research (Irvine, CA). Dynabeads MyOne Strepavidin T1 beads were purchased from Thermo Fisher Scientific (Waltham, MA). The MagneSphere 12-position rack was purchased from Promega (Madison, WI). RNase T1 and Turbo DNase were purchased from Thermo Fisher Scientific (Waltham, MA). Human RNase H1 was purchased from Abcam (Cambridge, UK). Refer to Supplementary Table 1 for DNA library and primer sequences. Refer to Supplementary Table 2 for threozyme sequences. Refer to Supplementary Table 3 for RNA substrate sequences.

### Library preparation for DNA display selection strategy

*Library design*. Inspired by the 10-23 DNAzyme framework, the DNA library features a central random region of 15 or 20 nucleotides (A:G:C:T = 25:25:25:25) as the catalytic core region. The random region is flanked by defined binding arm regions of 10 nucleotides that recognize the desired RNA substrate. Expanding further beyond both binding arm regions are the primer-binding sites of 20 nucleotides.

#### Hairpin ligation

A self-priming DNA library was generated by ligating a DNA library (5 µM) to a phosphorylated DNA hairpin primer (6 µM) in 1x T4 DNA ligase buffer using T7 DNA ligase (1 µM). The DNA library was annealed to the DNA hairpin primer by heating at 95 °C for 5 min and cooling on ice for 5 min. The reaction was then initiated with the addition of T7 DNA ligase and incubated overnight at 24 °C. The resulting self-priming DNA library was purified by 10% denaturing polyacrylamide gel electrophoresis (PAGE), electroeluted, and desalted using an Amicon^™^ ultra-centrifugal filter.

#### TNA transcription

The self-priming DNA library was extended with tNTPs to generate a chimeric DNA/TNA heteroduplex. The reaction was initiated with the addition of 10-92 TNA polymerase (2 µM) followed by tNTPs (0.1 mM of each tATP/tCTP/tGTP/tTTP) and incubated for 1 h at 40 °C. Afterward, an equal volume of phenol:chloroform:isoamyl alcohol (25:24:1v/v) was added to extract excess polymerase following the manufacturer’s protocol. Following extraction, the top aqueous layer was collected and desalted using an Amicon^™^ ultra-centrifugal filter.

#### Strand displacement

TNA was displaced by extending a chimeric 5’-biotin DNA primer containing the RNA substrate. First, the DNA primer (2 µM) was annealed to the hairpin loop of the library (1 µM) in 1x ThermoPol^®^ buffer by heating to 95 °C and cooling on ice for 5 min. The reaction was then supplemented with an additional 5 mM of MgCl_2_ before adding Bst 3.0 DNA polymerase (80 U/mL) and dNTPs (0.5 mM of each dATP/dCTP/dGTP/dTTP) to initiate the reaction and incubated for 1 h at 50 °C. Excess polymerase was extracted, and the aqueous layer was collected as described for TNA transcription. Residual phenol was removed by adding 500 µL of ethanol to the desalted solution, and the TNA library was dried by a SpeedVac concentrator.

### Selection by DNA display strategy

MyOne T1 streptavidin beads (35.7 µL) were pre-treated for RNA applications following manufacturer’s protocol and equilibrated in 500 µL of binding buffer (50 mM Tris (pH 7.5), 150 mM NaCl, 0.01% Tween-20, 0.5 mM EDTA) for 30 min at 24 °C with rotation. The TNA library (100 pmol) was then incubated with the pre-treated magnetic beads in 500 µL of binding buffer for 30 min at 24 °C with rotation to immobilize the library on the beads. The beads were washed 3-4 times with 500 µL of pre-cleavage buffer (50 mM Tris (pH 7.5), 10 mM NaCl, 140 mM KCl). Following the washes, 500 µL of cleavage buffer (50 mM Tris (pH 7.5), 10 mM NaCl, 140 mM KCl, 20 mM MgCl_2_) was added to the reaction tube to initiate RNA cleavage. The selection stringency was increased over 8 rounds at a constant temperature of 37 °C. The starting parameters were 20 mM MgCl_2_ and 19 h. The magnesium concentration and reaction time were incrementally decreased to a final condition of 0.5 mM MgCl_2_ and 15 min.

### Threozyme synthesis

All threozymes were prepared by solid-phase oligonucleotide synthesis using an automated Dr. Oligo DNA synthesizer on a 1-μmol scale universal CPG column (Glen Research) with optimized coupling times. Oligonucleotides were removed from the column and deprotected by incubation in ammonia gas for 2 h at 80 °C (60 psi), and purified by ion-pair reversed-phase HPLC followed by a Glen-Pak™ DNA Purification Cartridge (Glen Research). The samples were lyophilized to dryness, resuspended in water, UV-quantified, and verified by MALDI-TOF mass spectrometry.

### Activity assay

Threozymes were assayed under single turnover conditions with 500 nM Tz and 250 nM IR680-labeled RNA substrate (1:2, S:E) in a reaction buffer comprised of 50 mM Tris-HCl (pH 7.5), 10 mM NaCl, 140 mM KCl, and 20 mM MgCl_2_. Reactions were initiated with the addition of MgCl_2_ and incubated at either 37 °C or 50 °C for up to 24 hours. Aliquots (1.5 μL) of each reaction mixture were removed at designated time points and quenched in 16.5 μL formamide stop buffer (99% deionized formamide, 25 mM EDTA). Quenched samples were denatured at 95 °C for 10 min and resolved using 20% denaturing PAGE. The gel was imaged using an Odyssey CLx imaging system (LI-COR) and quantified using Image Studio 5.2 (LI-COR).

### Product validation

RNA substrate (1 μM) was digested by 1 U of RNase T1 in 50 mM Tris (pH 7.5), 10 mM NaCl, and 140 mM KCl at room temperature for 1 min to generate an RNA ladder. For threozyme-catalyzed reactions, 500 nM Tz and 250 nM IR680-labeled RNA substrate (1:2, S:E) were incubated in the same buffer used for RNase T1 digestion, but with the addition of 20 mM MgCl_2_. Samples were collected, quenched, and analyzed as described for the activity assay.

### Magnesium dependence screen

Threozymes were assayed under single turnover conditions with 500 nM Tz and 250 nM IR680-labeled RNA substrate (1:2, S:E) in reaction buffer comprised of 50 mM Tris-HCl (pH 7.5), 10 mM NaCl, 140 mM KCl, and varied concentrations of MgCl_2_ (0, 1, 5, 10, and 20 mM). Reactions were initiated with the addition of MgCl_2_ and incubated at 50 °C for 18 hours. Samples were collected, quenched, and analyzed as described for the activity assay.

### Temperature dependence screen

DNAzymes and threozymes were assayed across a range of temperatures. All reactions were incubated in 50 mM Tris (pH 7.5), 10 mM NaCl, and 140 mM KCl for 8 h. Dz catalyzed reactions contained 2 mM MgCl_2_ with 250 nM IR680-labeled RNA substrate and 25 nM enzyme, while the Tz catalyzed reactions contained 20 mM MgCl_2_ with 250 nM IR680-labeled or Cy5-labeled RNA substrate and 500 nM enzyme. Samples were collected, quenched, and analyzed as described for the activity assay.

### Turbo DNase assay

DNAzymes and threozymes were pretreated with Turbo DNase (2 U) in 50 mM Tris (pH 7.5), 10 mM NaCl, and 140 mM KCl at 37 °C for 10 min and 60 min, respectively. Dz catalyzed reactions contained 2 mM MgCl_2_, while Tz reactions contained 20 mM MgCl_2_. After preincubation, the IR680-labeled RNA substrate was added to the reaction, reaching final concentrations of 250 nM substrate and 250 nM enzyme (1:1, S:E). For the Dz reactions, temperature was maintained at 37 °C after RNA substrate addition and allowed to incubate for an additional hour. For the Tz reactions, the temperature was increased to 50 °C after RNA substrate addition and allowed to incubate for an additional 18 h.

### RNase H1 assay

DNAzymes and threozymes were assayed under single turnover with 250 nM enzyme and 250 nM IR680-labeled RNA substrate (1:1, S:E) in the absence and presence of RNase H1. All reactions were incubated in 50 mM Tris (pH 7.5), 10 mM NaCl, and 140 mM KCl at 37 °C. Dz catalyzed reactions contained 2 mM MgCl_2_, while the Tz catalyzed reactions contained 20 mM MgCl_2_. For the Tz reactions, the reaction temperature was increased to 50 °C for an additional 4 hours after 1 hour of initial incubation at 37 °C.

### Melting temperature (Tm) study

All melts were performed with 1:1 oligonucleotide stoichiometry (1 μM enzyme + 1 μM RNA substrate) in the same buffer used for the activity assay (50 mM Tris (pH 7.5), 10 mM NaCl, 140 mM KCl, and either 2 mM or 20 mM MgCl_2_). Prior to melting, the strands were annealed in an Eppendorf tube by heating to 95 °C for 5 minutes and cooling on ice. Melting curves were obtained in the forward melting direction in a quartz cuvette of 1-cm path length with a temperature gradient of 20° to 90 °C and a ramping rate of 1 °C per min by monitoring the change in UV absorbance at 260 nm using a Cary 100 UV-Vis spectrophotometer equipped with a Peltier thermal controller. Tm values were determined by the hyperchromicity method using Cary WinUV 4.2 (468).

### Rate constant (k_obs_) determination

Observed rate constants of single turnover assays were obtained by fitting the percentage of the cleaved substrate over the reaction time to the one-phase association curve (1) using LabPlot 2.10.1 (KDE).

1 $$Y={Y}_{0}+\left({Y}_{{plateau}}-{Y}_{0}\right)\times (1-{e}^{-{k}_{{obs}}\times t})$$

Y is the percent cleavage at finite time t, ${Y}_{0}$ is the percent cleavage at time equals zero, ${Y}_{{plateau}}$ is the percent cleavage at an infinite time where the reaction reaches a plateau, and ${k}_{{obs}}$ is the observed pseudo-first-order rate constant.

### Activation energy (E_a_) determination

Apparent activation energies were calculated using the two-point form of the Arrhenius Eq. (2).

2 $${{\mathrm{ln}}}\frac{{k}_{2}}{{k}_{1}}=\frac{{E}_{a}}{R}\left(\frac{1}{{T}_{1}}-\frac{1}{{T}_{2}}\right)$$

${T}_{1}$ and ${T}_{2}$ are temperatures (K) of the reaction, ${k}_{1}$ and ${k}_{2}$ are observed rate constants of the reaction at the respective temperatures, R is the universal gas constant (8.314 $J/{mol}\cdot K$), and ${E}_{a}$ is the activation energy.

### Statistics and reproducibility

The mean and standard deviation were calculated in Microsoft Excel 16.78 (23100802). All experiments were reproduced in sets of two or three independent replicates. No data were excluded from the analyses. Sample sizes were determined using sample sizes standard in nucleic acid research (duplicate or triplicate). The investigators were not blinded to allocation during the experiments and outcome assessments.

### Reporting summary

Further information on research design is available in the Nature Portfolio Reporting Summary linked to this article.

## Supplementary information

Supplementary Information

Reporting Summary

Transparent Peer Review file

## Source data

Source Data

## Data Availability

The authors declare that the data supporting the findings of this study are available within the article and its Supplementary Information file. All raw DNA sequences collected from the threozyme selection have been deposited in the Sequence Read Archive and can be found in the NCBI BioProject database under accession code PRJNA1499794 [https://www.ncbi.nlm.nih.gov/bioproject/PRJNA1499794]. Structural information referenced here can be found in the Protein Data Bank under accession code 7PDU. Source data are provided with this paper.
