# Supplementary material for: Dynamism and evolvability in nucleic acid enzymes
Source: Nat Commun. 2026 Aug 14;17:9757. doi: 10.1038/s41467-026-76473-9 (PMC13572484; doi:10.1038/s41467-026-76473-9)
Supplement: Supplementary file 1 [file 41467_2026_76473_MOESM1_ESM.pdf]

Supplementary Information

Dynamism and Evolvability in Nucleic Acid Enzymes

Erica M. Lee[1] and John C. Chaput[1-4]*

[1]Department of Pharmaceutical Sciences, University of California, Irvine, CA 92697-3958 USA
[2]Department of Chemistry, University of California, Irvine, CA 92697-3958 USA
[3]Department of Molecular Biology and Biochemistry, University of California, CA 92697-3958 USA
[4]Department of Chemical and Biomolecular Engineering, University of California, Irvine, CA 92697-3958 USA

Table of Contents

Supplementary Table 1. Oligonucleotides used in the threozyme selection.

Supplementary Table 2. Chemically synthesized threozymes used in the study.

Supplementary Table 3. RNA substrates used in the study.

Supplementary Table 4. Rate constants (k_{obs}) and activation energies (E_a).

Supplementary Figure 1. Schematic of DNA display.

Supplementary Figure 2. Threozyme screen for RNA-cleavage activity.

Supplementary Figure 3. Temperature-dependent RNA cleavage activity of top performing threozymes isolated by in vitro evolution.

Supplementary Figure 4. Cleavage site validation by Tz N15-1 and N20-1.

Supplementary Figure 5. Magnesium requirement for threozyme activity.

Supplementary Figure 6. Nucleic acid catalysis following pre-incubation with Turbo DNase.

Supplementary Figure 7. Nucleic acid catalysis in the absence and presence of RNase H1.

Supplementary Figure 8. Temperature-dependent RNA cleavage activity of Dz 10-23 under multiple turnover conditions.

Supplementary Figure 9. Temperature-dependent RNA cleavage activity of Dz 10-23 under single turnover conditions.

Supplementary Figure 10. Melting temperature of enzyme-substrate complexes.

Supplementary Figure 11. Temperature-dependent RNA cleavage activity of known threozymes that function with less constrained active sites.

Supplementary Figure 12. Comparison of temperature optimum for RNA cleavage.

Supplementary Figure 13. NMR-derived structural ensemble of Dz 10-23.

Supplementary Table 1. Oligonucleotides used in the threozyme selection.

Name	Sequence (5' → 3')
KRAS G12V N15 DNA Library	CTCTGCTGAAGCCAGTTACC AGTTGGAGCT(N15)TTGGCGTAGG GGTGGTATCCCCAAGGGGAC
KRAS G12V N20 DNA Library	CTCTGCTGAAGCCAGTTACC AGTTGGAGCT(N20)TTGGCGTAGG GGTGGTATCCCCAAGGGGAC
Hairpin Primer	pACTACGTACCCACAACCTCGGCCGTACCACGGTACGTAGT GTCCCCTTGGGGATACCACC
Strand Displacement Primer w/KRAS G12V RNA Substrate	Biotin-r(GUGGUAGUUGGAGCUGUUGGCGUAGG)GGTGGTATCC CCAAGGGGACACTACGTACCGTGGTACGGCCGAGGTTGTG
Reverse Primer PBS8	GTCCCCTTGGGGATACCACC
Forward Primer PBS23	CTCTGCTGAAGCCAGTTACC
PEGylated Forward Primer PBS23	AACAAACAAACAAACAAACA-iSp9-CTCTGCTGAAGCCAGTTACC

RNA (r); triethylene glycol spacer (iSp9).

Supplementary Table 2. Chemically synthesized threozymes used in the study.

Name	Sequence (3' → 2')	Calc. Mass (Da)	Obs. Mass (Da)	Δ Mass (Da)
N15-1	ACGCCAAACAACGGTAGTTGGAAGCTCCA	8498.2	8501.8	3.6
N15-2	ACGCCAAAATCGACCGAGCTTTAGCTCCA	8409.1	8412.2	3.1
N15-3	ACGCCAAACAATAGAAGCTGTTAGCTCCA	8457.2	8461.0	3.8
N15-4	ACGCCAATTAAAAACACTACAAAGCTCCA	8394.2	8398.0	3.8
N15-5	ACGCCAATTTACACGATACAAAAGCTCCA	8401.1	8404.4	3.3
N15-6	ACGCCAATTCACCGCTAGCTTGAGCTCCA	8376.1	8379.8	3.7
N20-1	ACGCCAAGAAAAGCCACAGAACTAAGTAGCTCCA	9939.1	9943.5	4.4
N20-2	ACGCCAAAATGGCCCTGTTAAGCGTTTAGCTCCA	9910.0	9914.7	4.7
N20-3	ACGCCAAGTATCGCTACTTACAAAACCAGCTCCA	9832.0	9836.7	4.7
N20-4	ACGCCAATGATAGAAGTTTCCTGGTCGAGCTCCA	9950.0	9954.1	4.1
N20-5	ACGCCAATGGAGTGCAACTAAGGTCAAAGCTCCA	9977.1	9980.8	3.7
N20-6	ACGCCAAATCGACTAGTATACACGAACAGCTCCA	9881.0	9884.7	3.7
6-29	TCGAGCTCTCAATAAGGGGATGAGTT	7685.7	7689.0	3.3
13-38	GTAGAGAAGTCTTATTGGTGGTTGAAGGGATGAGTT	10800.6	10808.1	7.5

Supplementary Table 3. RNA substrates used in the study.

Name	Sequence (5' → 3')
KRAS G12V RNA	IR680-AGUUGGAGCUGUUGGCGUAGG
EGFR T790M RNA	Cy5-AACUCAUCAUGCAGCUCGA

Supplementary Table 4. Rate constants (k_{obs}) and activation energies (E_a).

Enzyme	Temp (˚C)	k_{obs} (h^{-1})	± SE	E_a (kJ/mol)	± SE
Tz N15-1	37	0.02	0.002	77.5	7.2
	50	0.06	0.0008		
Tz N20-1	37	0.02	0.0008	79.4	2.7
	50	0.07	0.001		
Tz 6-29	37	0.07	0.004	9.1	5.3
	50	0.08	0.005		
Tz 13-38	37	0.27	0.02	-47.0	5.7
	50	0.13	0.008		
Dz 10-23	37	21.7	1.1	-158.2	14.4
	50	1.8	0.4		

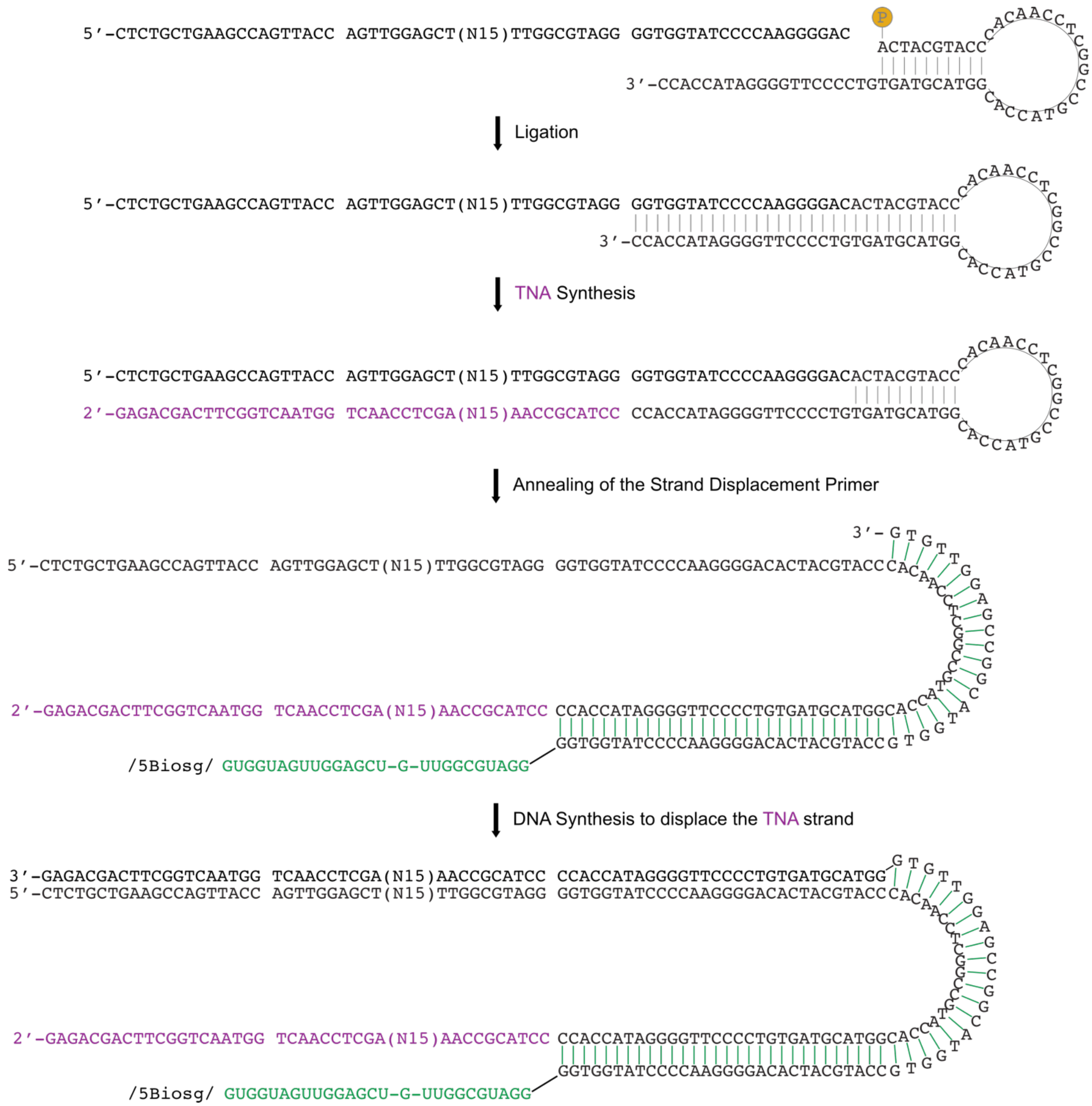

Supplementary Figure 1. Schematic of DNA display. A DNA stem-loop structure is ligated to a DNA library, extended with TNA, and the resulting hairpin undergoes a strand displacement reaction by extending a DNA substrate annealed to the loop region with DNA. Colors: DNA (black), TNA (purple), and RNA (green).

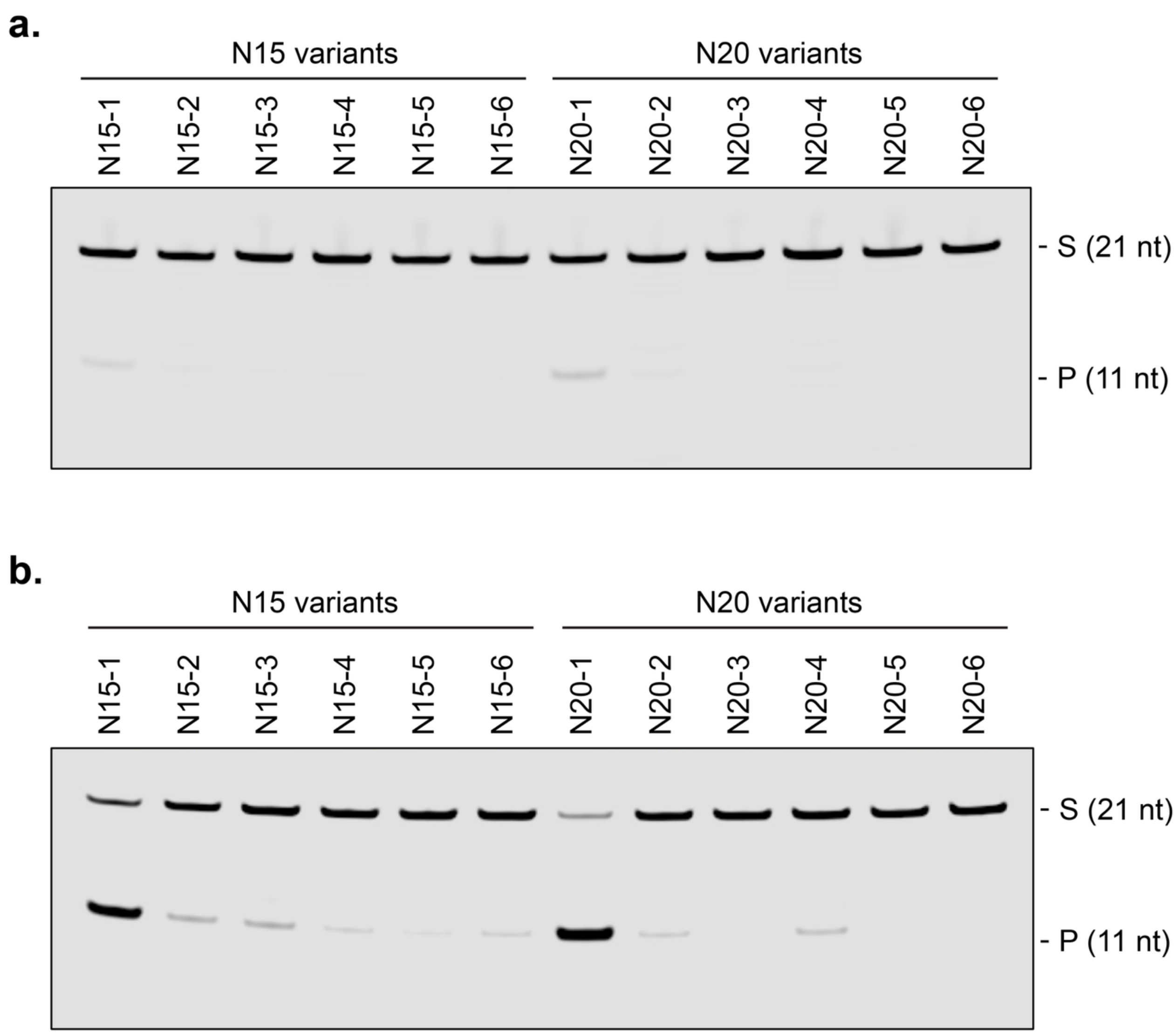

Supplementary Figure 2. Threozyme screen for RNA-cleavage activity. a-b. Representative denaturing PAGE gels of the six most enriched sequences from the N15 and N20 libraries screened for activity at 37°C (panel a) and 50°C (panel b), respectively. All reactions (n=3 independent experiments) were performed in 50 mM Tris (pH 7.5), 10 mM NaCl, 140 mM KCl, and 20 mM $MgCl_2$ at either 37°C or 50°C for 24 hours with 250 nM substrate and 500 nM enzyme (1:2, S:E). S: 5'-IR680 labeled full-length substrate, P: 5'-IR680 labeled cleavage product. Molecular weight markers indicated to the right of the gel. Source data for this figure is available in the Source Data file.

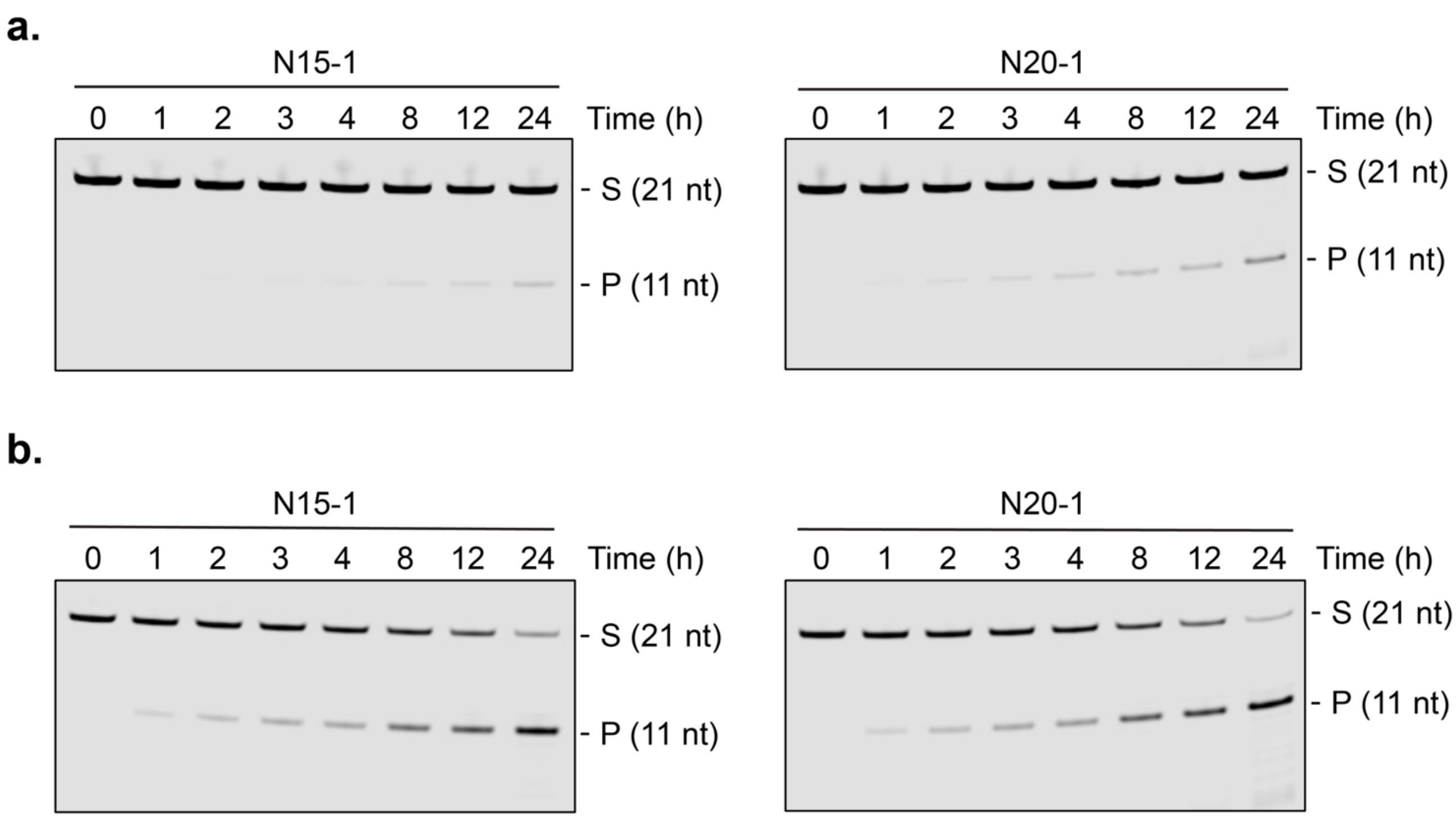

Supplementary Figure 3. Temperature-dependent RNA cleavage activity of top performing threozymes isolated by in vitro evolution. a-b. Representative denaturing PAGE gels of the most active threozymes from the N15 and N20 libraries assessed in time course reactions performed at 37°C (panel a) and 50°C (panel b), respectively. All reactions (n=3 independent experiments) were performed in 50 mM Tris (pH 7.5), 10 mM NaCl, 140 mM KCl, and 20 mM $MgCl_2$ at either 37°C or 50°C with 250 nM substrate and 500 nM enzyme (1:2, S:E). S: 5'-IR680 labeled full-length substrate, P: 5'-IR680 labeled cleavage product. Molecular weight markers indicated to the right of the gel. Source data for this figure is available in the Source Data file.

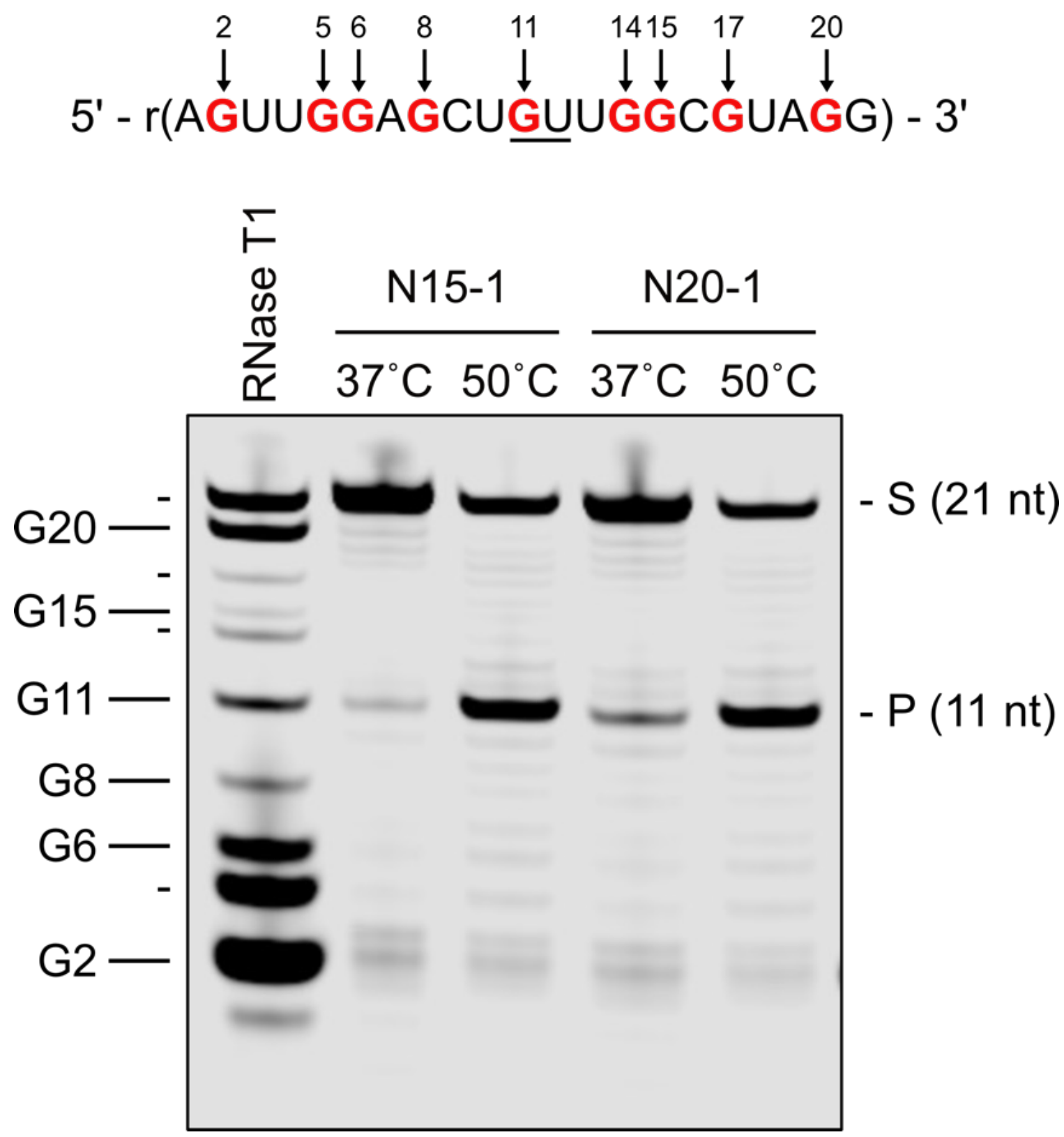

Supplementary Figure 4. Cleavage site validation by Tz N15-1 and N20-1. Schematic depiction of the 21-nt RNA substrate showing the RNase T1 cut sites after G residues (red) and desired G-U cleavage junction (underlined). A denaturing PAGE gel showing the full-length substrate and upstream product of threozyme-mediated cleavage reactions alongside an RNA ladder produced by RNase T1 digestion. Threozyme reactions were performed in 50 mM Tris (pH 7.5), 10 mM NaCl, 140 mM KCl, and 20 mM $MgCl_2$ at either 37˚C or 50°C with 250 nM substrate and 500 nM enzyme (1:2, S:E). S: 5'-IR680 labeled full-length substrate, P: 5'-IR680 labeled cleavage product. Molecular weight markers indicated to the right of the gel. Source data for this figure is available in the Source Data file.

a.

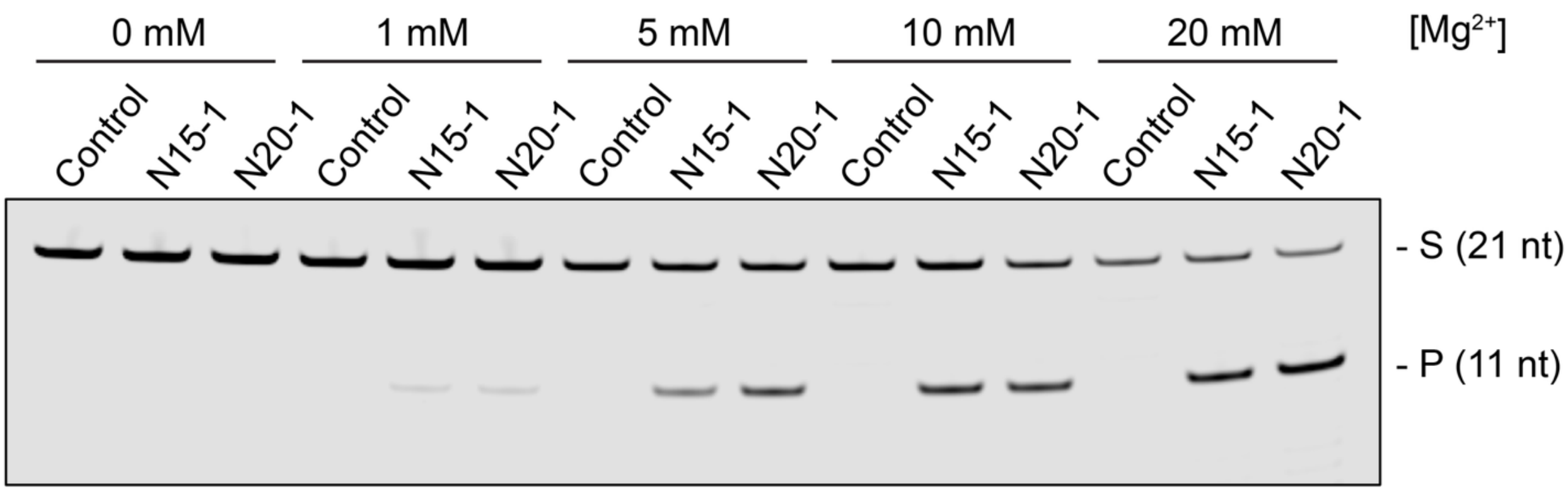

b.

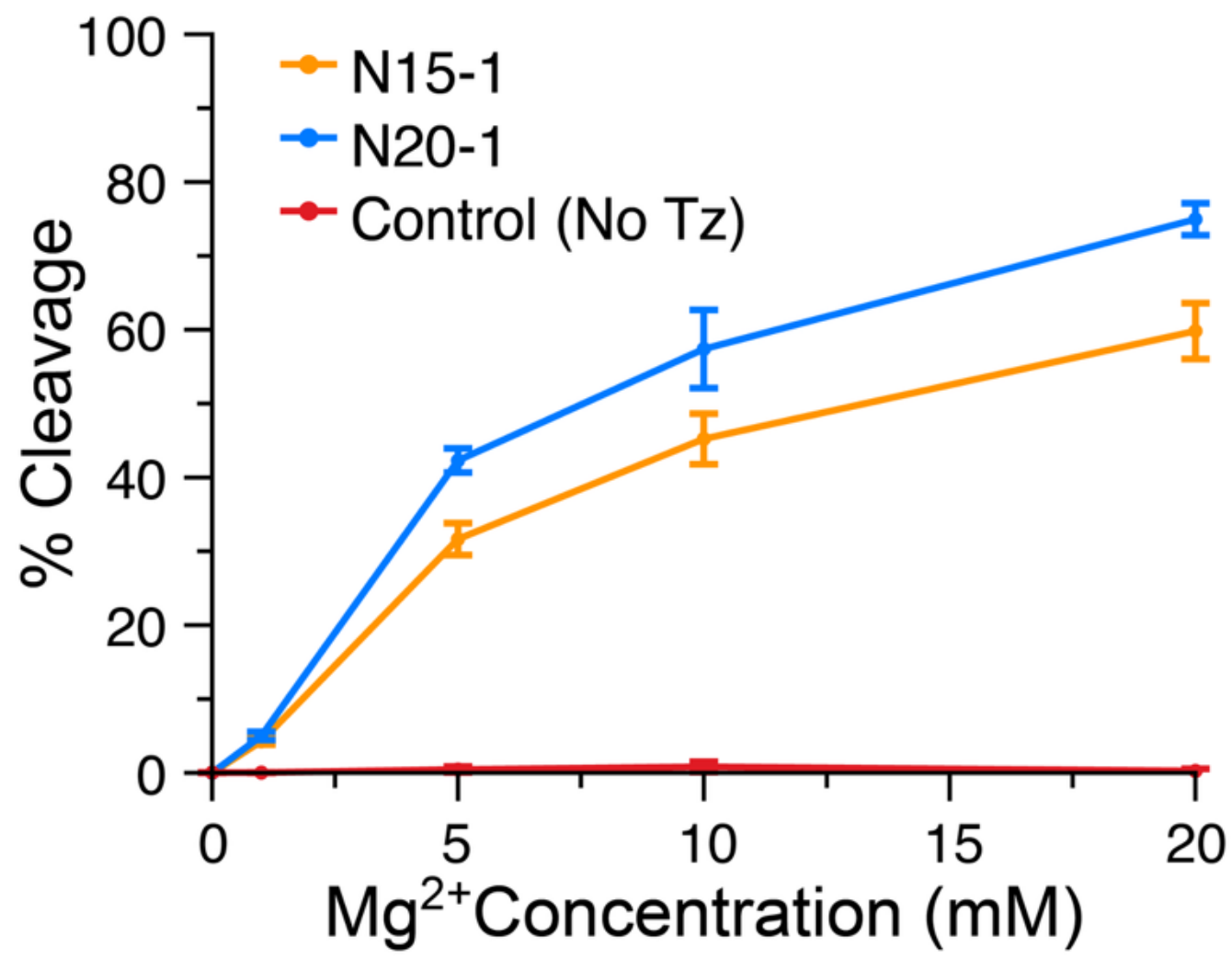

Supplementary Figure 5. Magnesium requirement for threozyme activity. a. Representative denaturing PAGE gel of Tz N15-1 and N20-1 assessed in the presence of increasing concentration of Mg^{2+} (0 – 20 mM) after 18 hours of incubation at 50°C. **b.** Plot of catalytic activity as a function of magnesium concentration. Data presented as the mean ± standard deviation (n=3 independent experiments). All reactions were performed in 50 mM Tris (pH 7.5), 10 mM NaCl, and 140 mM KCl with 250 nM substrate and 500 nM enzyme (1:2, S:E). S: 5'-IR680 labeled full-length substrate, P: 5'-IR680 labeled cleavage product. Source data for this figure is available in the Source Data file.

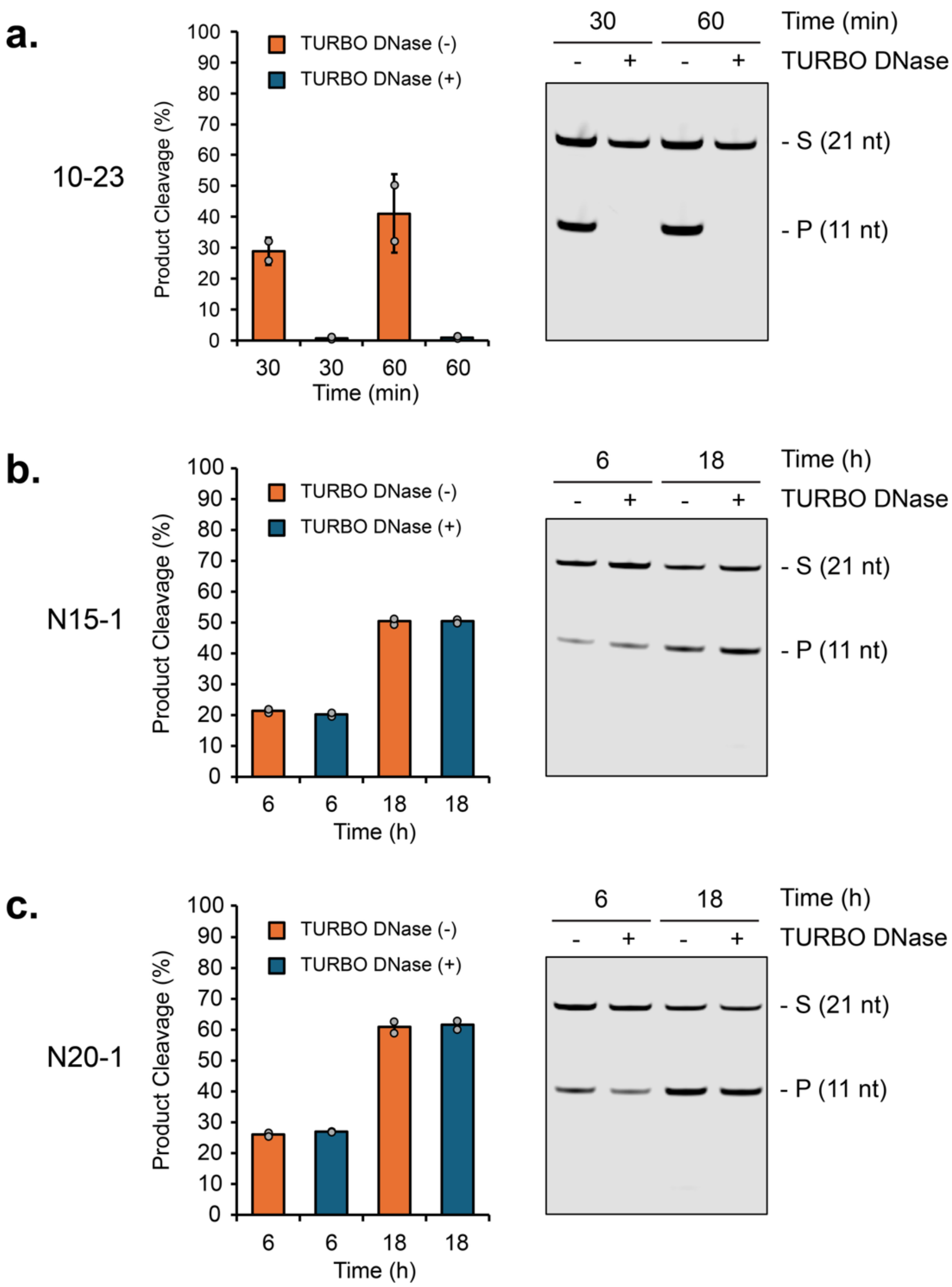

Supplementary Figure 6. Nucleic acid catalysis following preincubation with Turbo DNase. a. Representative denaturing PAGE gel of Dz 10-23 mediated RNA cleavage in the absence and presence of Turbo DNase. **b-c.** Representative denaturing PAGE gels of Tz N15-1 and N20-1 mediated RNA cleavage in the absence and presence of Turbo DNase. Data presented as the mean ± standard deviation (n=2 independent experiments). All reactions were performed by pretreating the catalysts with Turbo DNase in 50 mM Tris (pH 7.5), 10 mM NaCl, and 140 mM KCl at 37°C before addition of the RNA substrate to the reaction. Dz catalyzed reactions contained 2 mM $MgCl_2$, while Tz reactions contained 20 mM $MgCl_2$. For the Tz reactions, the temperature was increased to 50°C after the initial preincubation period and RNA substrate addition. S: 5'-IR680 labeled full-length substrate, P: 5'-IR680 labeled cleavage product. Source data for this figure is available in the Source Data file.

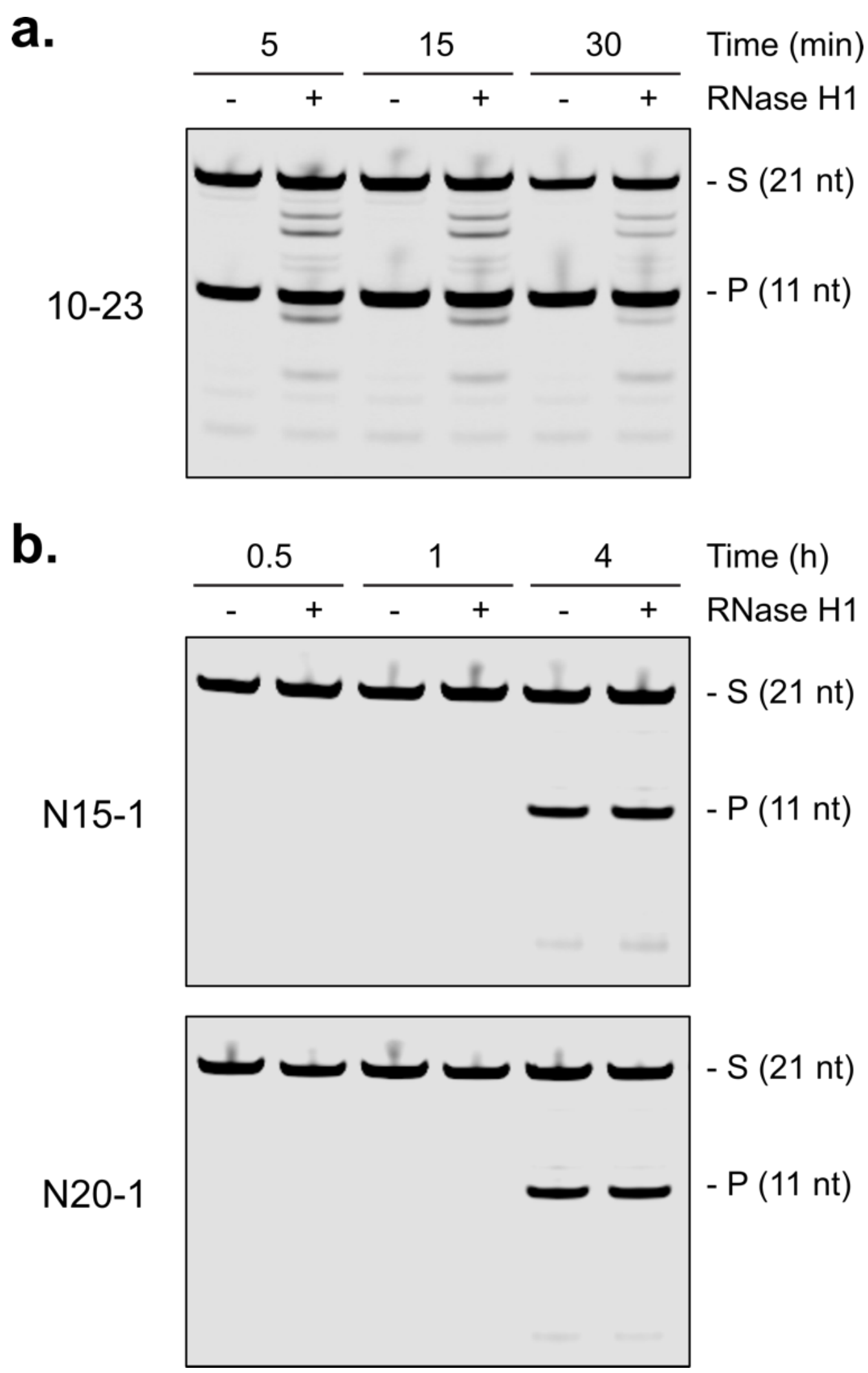

Supplementary Figure 7. Nucleic acid catalysis in the absence and presence of RNase H1. a. Representative denaturing PAGE gel of Dz 10-23 mediated RNA cleavage in the absence and presence of human RNase H1. **b.** Representative denaturing PAGE gels of Tz N15-1 and N20-1 mediated RNA cleavage in the absence and presence of human RNase H1. All reactions (n=2 independent experiments) were performed in 50 mM Tris (pH 7.5), 10 mM NaCl, and 140 mM KCl with 250 nM substrate and 250 nM enzyme (1:1, S:E) at 37˚C. Dz catalyzed reactions contained 2 mM $MgCl_2$, while the Tz catalyzed reactions contained 20 mM $MgCl_2$. After 1 hour of incubation at 37˚C, the temperature of the Tz reaction was increased to 50°C for an additional 4 hours. S: 5'-IR680 labeled full-length substrate, P: 5'-IR680 labeled cleavage product. Source data for this figure is available in the Source Data file.

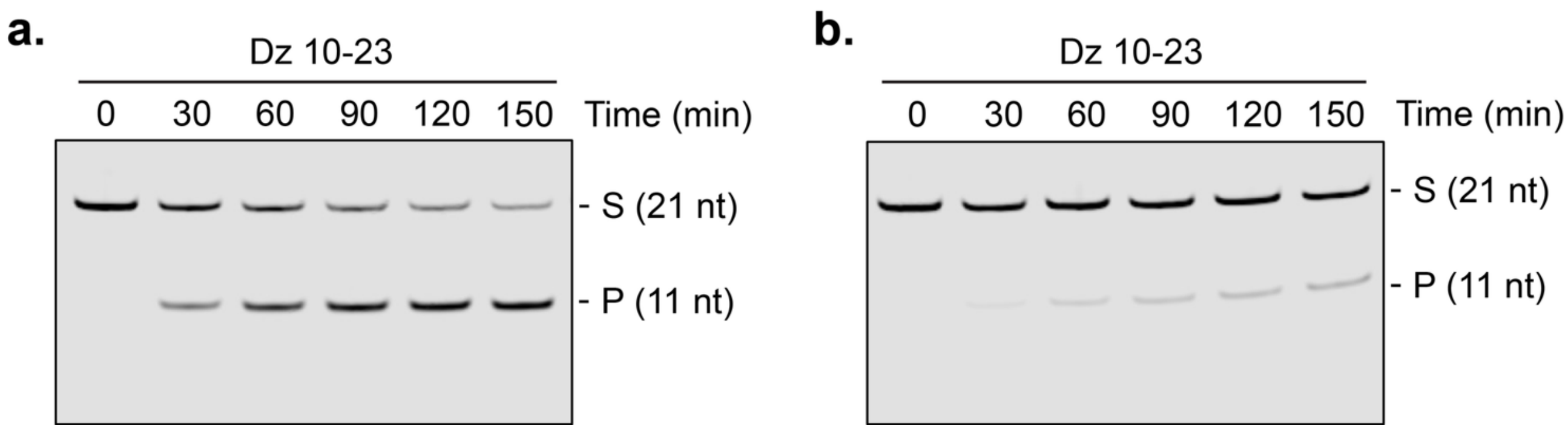

Supplementary Figure 8. Temperature-dependent RNA cleavage activity of Dz 10-23 under multiple turnover conditions. a-b. Representative denaturing PAGE gels of 10-23 activity in time course reactions performed at 37°C (panel a) and 50°C (panel b), respectively. All reactions (n=3 independent experiments) were performed in 50 mM Tris (pH 7.5), 10 mM NaCl, 140 mM KCl, and 2 mM $MgCl_2$ at either 37°C or 50°C with 250 nM substrate and 25 nM enzyme (10:1, S:E). S: 5'-IR680 labeled full-length substrate, P: 5'-IR680 labeled cleavage product. Molecular weight markers indicated to the right of the gel. Source data for this figure is available in the Source Data file.

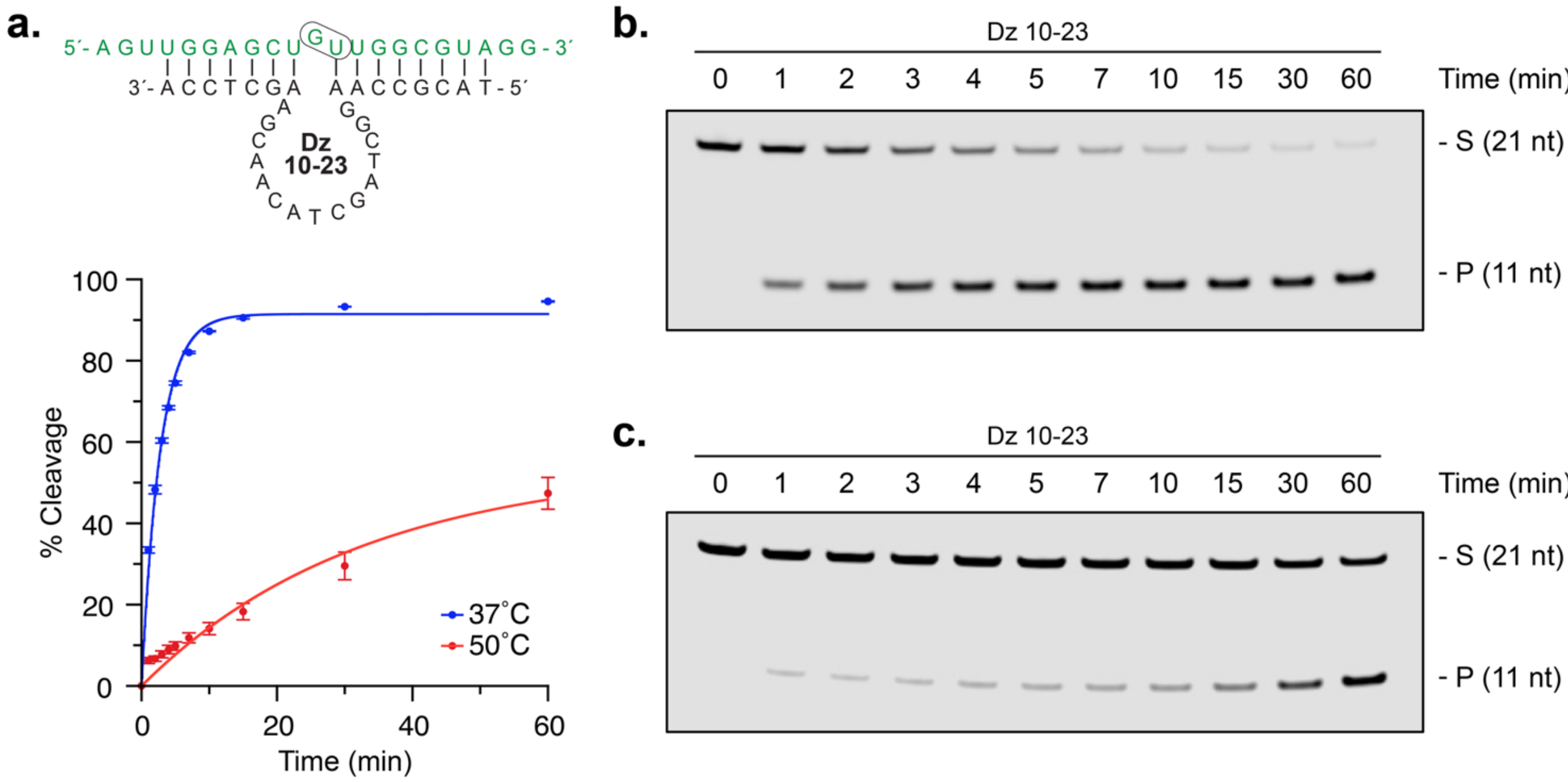

Supplementary Figure 9. Temperature-dependent RNA cleavage activity of Dz 10-23 under single turnover conditions. a. Kinetic curves of Dz 10-23. Reaction profiles show RNA cleavage at 37°C (blue) and 50°C (red). Enzyme-substrate complex is provided above the plot. The cleavage site is denoted with a circle. **b-c.** Representative denaturing PAGE gels of 10-23 activity in time course reactions performed at 37°C (panel b) and 50°C (panel c), respectively. Data presented as the mean ± standard deviation (n=3 independent experiments). All reactions were performed in 50 mM Tris (pH 7.5), 10 mM NaCl, 140 mM KCl, and 2 mM $MgCl_2$ at either 37°C or 50°C with 0.5 μM substrate and 2.5 μM enzyme (1:5, S:E). S: 5'-IR680 labeled full-length substrate, P: 5'-IR680 labeled cleavage product. Molecular weight markers indicated to the right of the gel. Source data for this figure is available in the Source Data file.

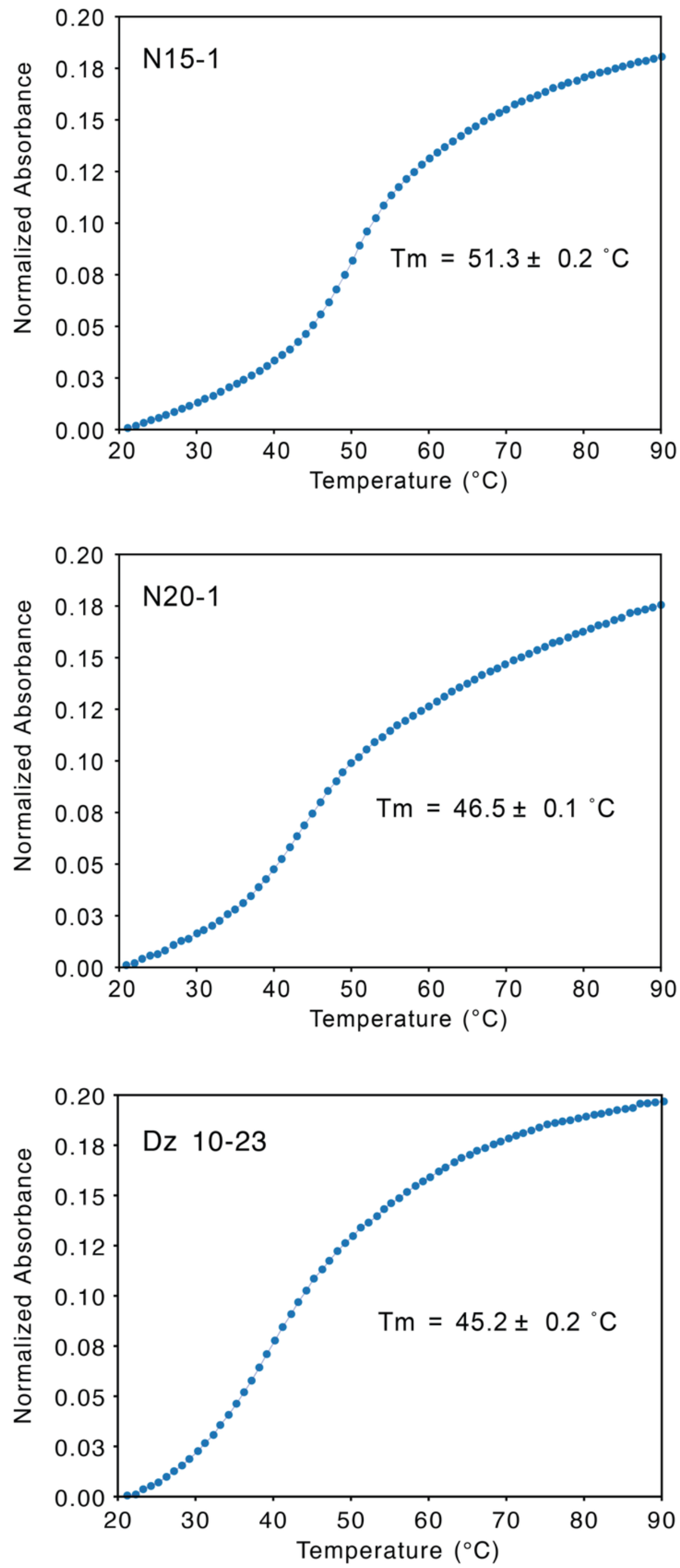

Supplementary Figure 10. Melting temperature of enzyme-substrate complexes. An uncleavable 21-nt RNA substrate with a 2′-deoxyribose G substitution at the G-U dinucleotide cleavage junction was used for melt measurements. All melts were performed with 1:1 oligonucleotide stoichiometry (1 µM enzyme + 1 µM RNA substrate) in the Tz and Dz reaction buffers. Melting curves were obtained (n=2 independent experiments) in the forward direction in a quartz cuvette with a 1-cm path length and a temperature range of 20º to 90ºC. Thermal denaturation was performed with a ramping rate of 1°C per min by monitoring changes in UV absorbance (260 nm). Source data for this figure is available in the Source Data file.

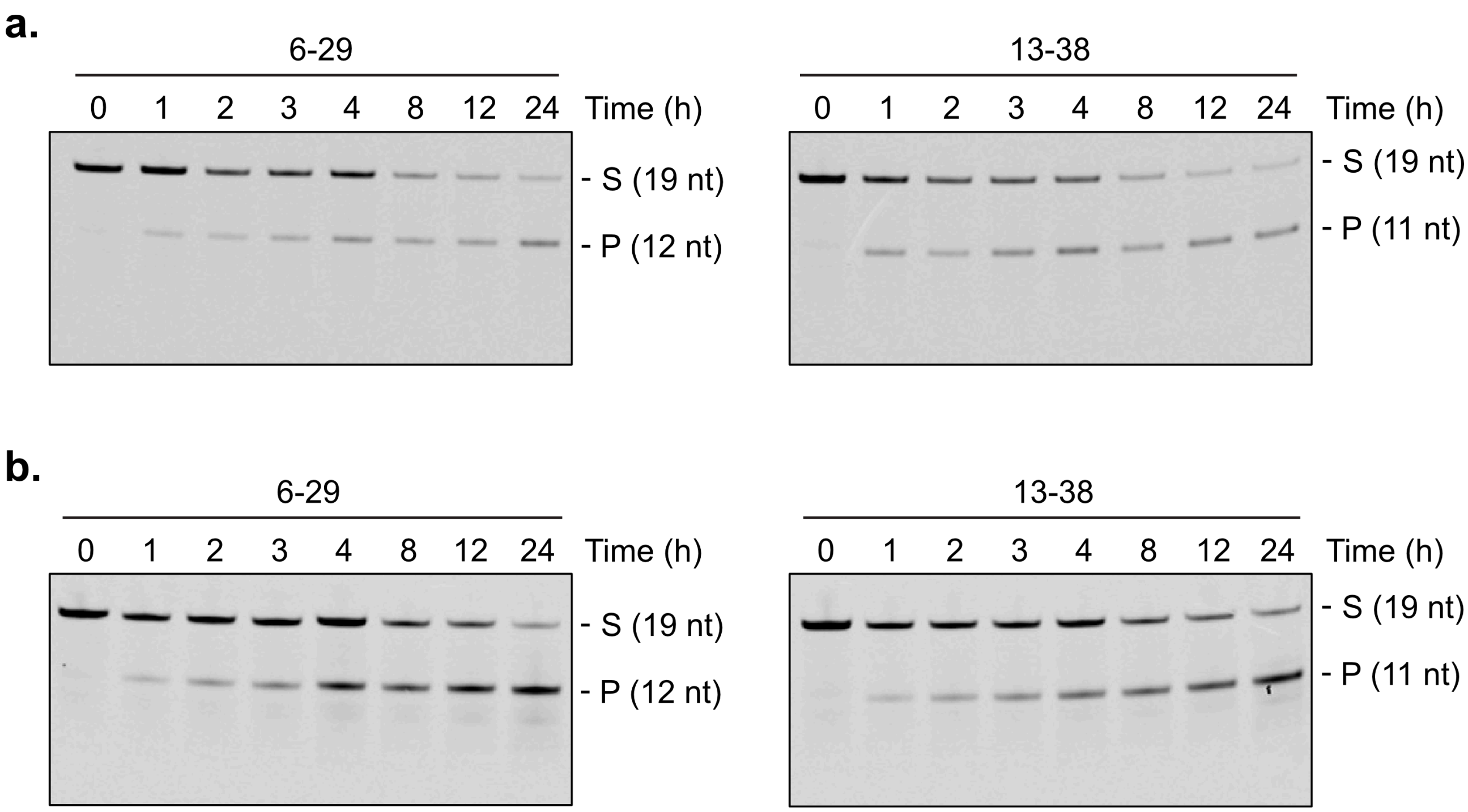

Supplementary Figure 11. Temperature-dependent RNA cleavage activity of known threozymes that function with more flexible active sites. a-b. Representative denaturing PAGE gels of threozyme activity in time course reactions performed at 37°C (panel a) and 50°C (panel b), respectively. All reactions (n=3 independent experiments) were performed in 50 mM Tris (pH 7.5), 10 mM NaCl, 140 mM KCl, and 20 mM $MgCl_2$ at either 37°C or 50°C with 250 nM substrate and 500 nM enzyme (1:2, S:E). S: 5'-Cy5 labeled full-length substrate, P: 5'-Cy5 labeled cleavage product. Molecular weight markers indicated to the right of the gel. Source data for this figure is available in the Source Data file.

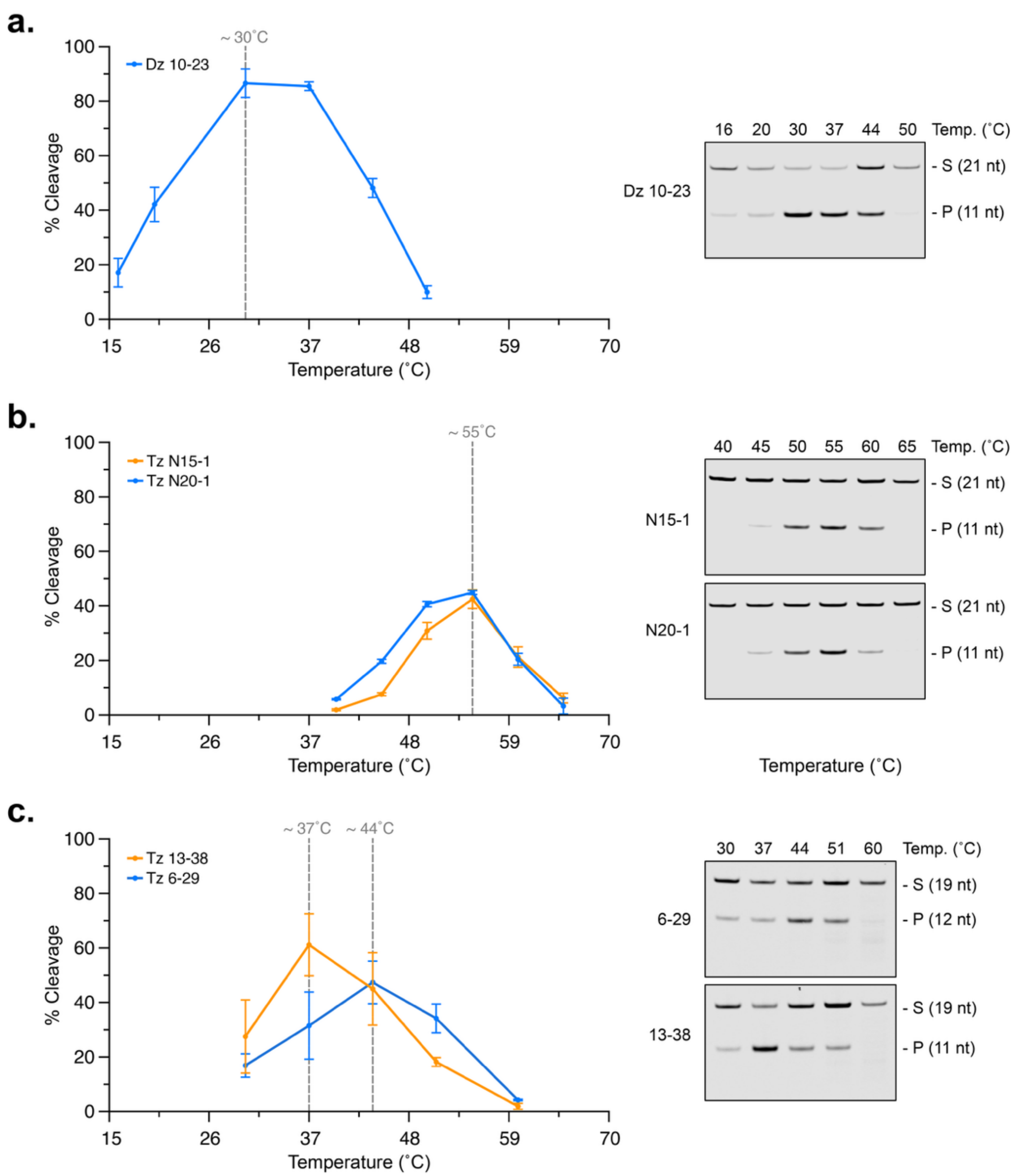

Supplementary Figure 12. Comparison of temperature optimum for RNA cleavage. a-c. Plots and representative denaturing PAGE gel of RNA cleavage activity at increasing temperatures for Dz 10-23 (panel a), Tz N15-1 and N20-1 with constrained active sites (panel b), and Tz 6-29 and 13-38 with flexible active sites (panel c). Data presented as the mean ± standard deviation (n=3 independent experiments). All reactions were performed in 50 mM Tris (pH 7.5), 10 mM NaCl, and 140 mM KCl with 8 hours of incubation. Dz catalyzed reactions contained 2 mM $MgCl_2$ with 250 nM substrate and 25 nM enzyme, while the Tz catalyzed reactions contained 20 mM $MgCl_2$ with 250 nM substrate and 500 nM enzyme. S: 5'-Cy5 labeled full-length substrate, P: 5'-Cy5 labeled cleavage product. Molecular weight markers indicated to the right of the gel. Source data for this figure is available in the Source Data file.

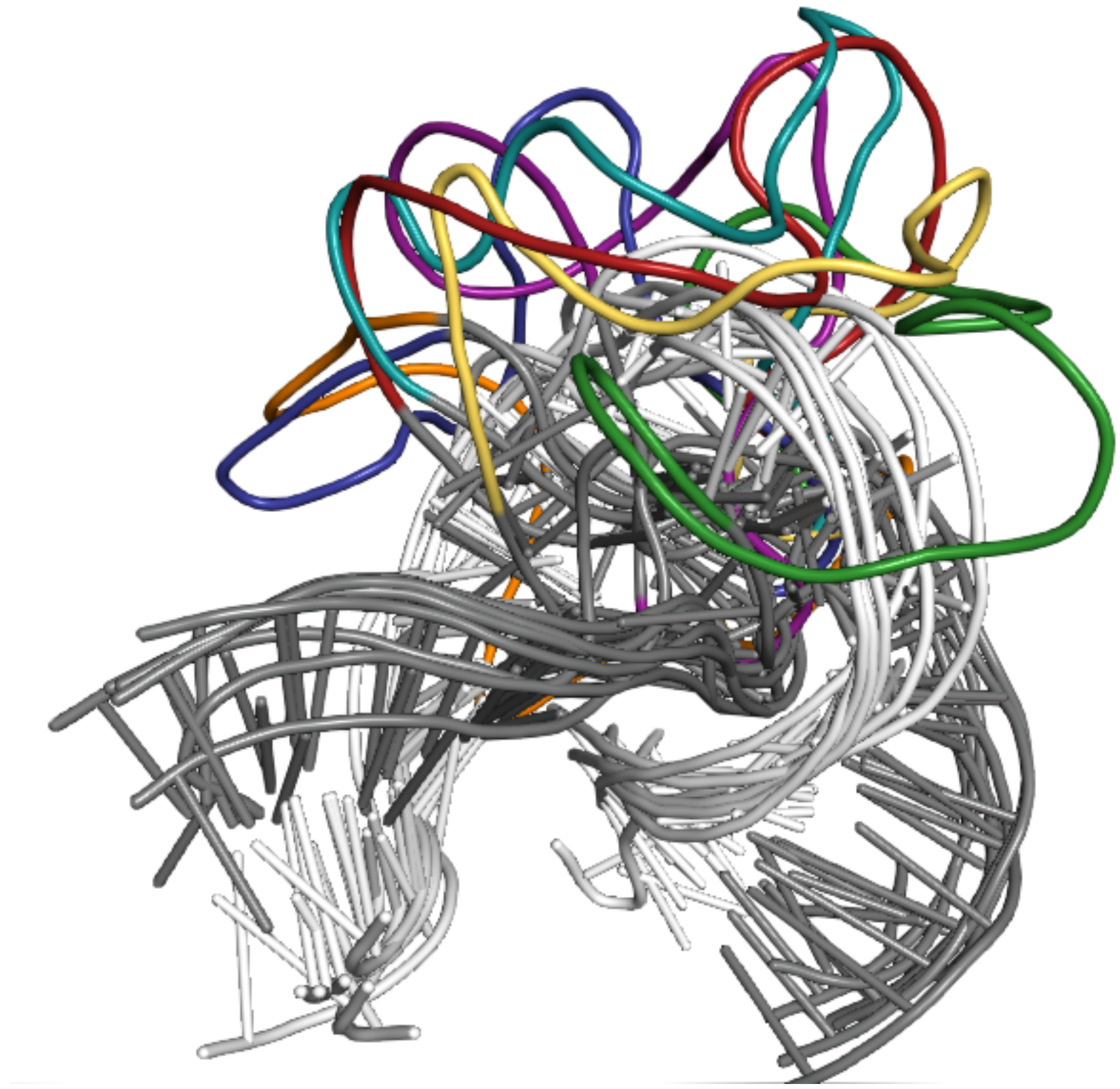

Supplementary Figure 13. NMR-derived structural ensemble of Dz 10-23. NMR analysis of Dz 10-23 (PDB: 7PDU) showing a highly dynamic catalytic loop (colored loops) displayed on more rigid substrate binding arms (grey).
