# Supplementary material for: Dynamism and evolvability in nucleic acid enzymes
Source: Nat Commun. 2026 Aug 14;17:9757. doi: 10.1038/s41467-026-76473-9 (PMC13572484; doi:10.1038/s41467-026-76473-9)
Supplement: Supplementary file 3 [file 41467_2026_76473_MOESM3_ESM.pdf]

nature portfolio

Peer Review File

Dynamism and Evolvability in Nucleic Acid Enzymes

Corresponding Author: Professor John Chaput

This file contains all reviewer reports in order by version, followed by all author rebuttals in order by version.

Version 0:

Reviewer comments:

Reviewer #1

(Remarks to the Author)
This is an excellent study that addresses fundamental question in nucleic acid chemistry: how backbone structure influences the evolvability of catalytic nucleic acids. The authors pose clear and compelling question, whether conformational restriction in nucleic acid backbones, exemplified here by the nucleic acid mimic TNA, imposes constraints on the emergence and optimisation of catalytic function.

Using established in vitro selection methodologies, the authors successfully isolate two TNA enzymes capable of cleaving the same substrate the well-characterised 10–23 DNAzyme. Through kinetic and temperature-dependent analyses, they demonstrate that these TNA enzymes exhibit enhanced catalytic activity at elevated temperatures. This behaviour is interpreted reflecting higher energetic barrier to formation of productive active site in TNA relative to DNA. Importantly, the authors further show that variants with increased active site flexibility display reduced temperature dependence, providing coherent mechanistic link between backbone rigidity, conformational dynamics, and catalytic efficiency.

far I can see, the methodology is sound and consistent with current standards in the field. The use of in vitro selection, combined with kinetic and temperature-dependent assays, is appropriate for addressing the stated research question. The comparison to classic DNAzyme system further strengthens the study design.

Some minor points:

1. Could the increased activity at higher temperature reflect effects on substrate binding, metal ion coordination, or global folding equilibria rather than solely active site formation. greater discussion of these alternative contributions would improve balance and clarity and would not detract from the overall conclusions.

2. I also wonder what would have happened if the authors had attempted the selection experiments conditions at 50 and not 37 degC? Perhaps not possible, but did the authors try this?

Given the growing interest in xeno nucleic acids and their implications for synthetic biology and origins-of-life research, this work is both significant and timely. Excellent work, well done.

Reviewer #2

(Remarks to the Author)
The authors have used in vitro selection to identify two TNAzymes capable of hydrolyzing RNA substrates. Interestingly, despite being selected at 37°C, both TNAzymes were poor catalysts at this temperature. Catalysis was much improved when the temperature was increased to 50°C. To shed light into this discrepancy, the authors investigated the temperature dependence of other TNAzymes well one of the most efficient DNAzymes (Dz10-23). This analysis revealed that Dz10-23 displayed an opposite tendency, i.e. performed well at 37°C but not at 50°C, while the other TNAzymes displayed an intermediate behaviour since they catalyzed RNA hydrolysis at both temperatures. The authors conclude that both the effect of conformationally restrained backbone of TNA and the nature of the active site dictated the efficiency of nucleic acid catalysts. All experiments were carried out in competent manner, the manuscript is very well-written and clear, and the supporting information is of good quality. This manuscript addresses very interesting question and of fundamental

importance to derive more potent nucleic acid-based catalysts. However, the extent of the effects of the rigidity of the backbone and that of the conformation of the active site are difficult to separate and quantify. In addition, the conclusions of this manuscript rely on very limited set of different sugar chemistries and substrates (see comments below). Hence, this manuscript could be considered for publication in Nature Communications if additional data are provided and the following revisions are considered:

- The authors should evaluate the temperature dependence of Dz10-23 with more flexible active site. Temperature dependence might be identical to that observed with the less flexible active site but will allow for direct comparison with Tz6-29 and Tz13-38 in Figure 4.
- The authors should also evaluate the temperature dependence of nucleic acid enzymes with other backbones including small ribozymes cleaving KRAS substrates (see e.g. 10.1016/j.chembiol.2026.03.001; 10.1054/bjoc.2000.1363), FANAzymes (e.g. 10.1038/s41557-022-01021-z), LNAzyme (e.g. 10.1039/d5ra00161g) and HNAzyme (10.1038/nature13982) which will have different degrees of conformational flexibility. These experiments are important to further confirm the hypothesis of strong effect of backbone structure on catalysis.
- The authors should evaluate whether increasing the temperature to 50°C during the in vitro selection protocol would yield better catalysts against this target RNA. Temperature of in vitro selection has been shown to be an important parameter (10.1093/nar/gkh1007).
- The authors may want to nuance their conclusions in the discussion section because besides backbone structure and flexibility of the active site, the presence of modifications on the nucleobase can change the outcome of selection experiment and the temperature dependence (see e.g. 10.1002/cbic.202100600; 10.1039/C5MB00102A; 10.1093/nar/gkn1070).
- The observed first-order rate constants of all catalysts should be provided in the main manuscript.

Reviewer #3

(Remarks to the Author)
The manuscript, “Dynamism and Evolvability in Nucleic Acid Enzymes,” examines how backbone chemistry may influence the catalytic behavior of nucleic acid enzymes. The authors compare the well-known 10-23 DNAzyme with newly selected TNA-based RNA-cleaving enzymes, or threozymes, acting on related RNA substrate. The main finding is that 10-23 DNAzyme is active at 37°C but loses much of its activity at 50°C, whereas the selected threozymes show weak activity at 37°C but substantially enhanced cleavage at 50°C. The authors further compare these enzymes with previously reported threozymes that contain more flexible active-site architectures. Overall, the work addresses an interesting question at the interface of nucleic acid chemistry, XNA biology, catalysis, and molecular evolution. The manuscript is clearly written, and the DNA-display selection strategy is technically interesting.

However, I think the manuscript requires major revision before publication. The main concern is that the conceptual interpretation—linking TNA backbone rigidity to catalytic dynamism and evolvability—is broader than what the current data can fully support. The temperature-dependent activity difference is interesting, but additional experimental and quantitative support would make the conclusions more convincing.

1. The key experiments should be repeated with stronger statistical support: Many central experiments, including the activity screens, kinetic curves, melting analyses, and nuclease-resistance assays, are reported with n = 2. Given that the manuscript makes broad mechanistic claims about temperature-dependent catalysis, conformational constraints, and evolvability, the key quantitative experiments should ideally be repeated with at least n = 3 independent replicates, with appropriate error analysis. This is especially important for Figures 3 and 4 and the associated supplementary figures.

2. The comparison between DNAzyme and threozymes should be better controlled or more cautiously interpreted: The DNAzyme and threozyme reactions are performed under substantially different conditions. For example, the DNAzyme reactions use 2 mM $MgCl_2$, 25 nM enzyme, and 250 nM substrate, whereas the threozyme reactions use 20 mM $MgCl_2$, 500 nM enzyme, and 250 nM substrate. These differences may be necessary, but they complicate direct comparison of catalytic performance and temperature dependence. The authors should either provide matched-condition experiments, where feasible, particularly enzyme/substrate settings, or more explicitly explain the rationale and limitations of the comparison.

3. The mechanistic claim about higher free-energy barrier should be softened or supported by additional analysis: The data clearly show that the selected threozymes are more active at elevated temperature. However, the conclusion that TNA catalysis “must overcome higher free-energy barrier” is stronger than the current evidence allows. The authors could strengthen this point by reporting apparent rate constants over temperature range and, if possible, performing Arrhenius or Eyring-type analysis. Without such analysis, the manuscript should describe the results more cautiously temperature-dependent activation rather than direct evidence for defined energetic barrier.

4. The term “evolvability” should be used more carefully: The manuscript is framed around dynamism and evolvability, but evolvability itself is not directly measured. The study identifies TNA catalysts and compares their temperature-dependent activity, but it does not directly assess adaptive trajectories, mutational robustness, or the accessible diversity of catalytic solutions relative to DNA under parallel selection conditions. The authors may retain evolvability broader conceptual framework, but statements suggesting that the study establishes fundamental limits on TNA evolvability should be moderated.

5. The relationship between selection conditions and post-selection activity deserves clarification: The threozyme selections were performed at 37°C, yet the best selected threozymes show much stronger activity at 50°C in the trans-cleavage assays.

This is intriguing but should be discussed more clearly. Are the selected catalysts more active in the original DNA-display format at 37°C than in the trans assay? Could the display architecture stabilize productive conformation? Clarifying this point would help readers understand why selection at 37°C yielded enzymes that appear to function best at elevated temperature.

6. The biological relevance should be presented cautiously: The nuclease-resistance experiments are useful and support the known biostability of TNA. However, the newly selected threozymes require elevated temperature and high Mg^{2+} concentration for strong activity. The manuscript should therefore distinguish more clearly between the conceptual importance of these catalysts and their immediate practical applicability in biological environments.

7. Writing and presentation issues: The manuscript is generally well written, but several minor issues should be corrected. For example, “randoms TNA sequences” should be changed to “random TNA sequences”; “a only limited set” should be “only limited set”; “It’s worth noting” should be changed to “It is worth noting”; “Noticeably” should be changed to “Notably”. The authors should also define what the error bars represent in all relevant figure legends.

In summary, this is an interesting and potentially important study that raises meaningful question about how nucleic acid backbone chemistry influences catalysis. I support publication after major revision, provided that the authors strengthen the statistical and kinetic support for the key findings, clarify the basis of the DNAzyme–threozyme comparison, and moderate the broader claims about free-energy barriers and evolvability.

Version 1:

Reviewer comments:

Reviewer #1

(Remarks to the Author)
The authors have satisfactorily addressed my questions and support publication.

Reviewer #2

(Remarks to the Author)
The authors have revised this manuscript to account for the comments of three reviewers (I was Referee#2), who were all supportive of publication. My original review included various comments, and the authors have now comprehensively addressed all the points that I raised and certainly markedly improved the quality of the manuscript. The authors have carried out an additional experiment recommended by reviewer#3 (Arrhenius analysis for all enzymes). Performing new selection at 50°C suggested by reviewers #1 and #2 (and alluded to by reviewer #3) might have improved the quality of the manuscript but is certainly too labor intensive. Hence, publication in Nature Communications is now recommended.

Reviewer #3

(Remarks to the Author)
I am pleased with the responses to my comments made on the original manuscript and the revisions made to the revised manuscript. I support the acceptance of this work for the publication in Nature Communications.

Open Access This Peer Review File is licensed under a Creative Commons Attribution 4.0 International License, which permits use, sharing, adaptation, distribution and reproduction in any medium or format, as long as you give appropriate credit to the original author(s) and the source, provide a link to the Creative Commons license, and indicate if changes were made.
In cases where reviewers are anonymous, credit should be given to 'Anonymous Referee' and the source.
The images or other third party material in this Peer Review File are included in the article's Creative Commons license, unless indicated otherwise in a credit line to the material. If material is not included in the article's Creative Commons license and your intended use is not permitted by statutory regulation or exceeds the permitted use, you will need to obtain permission directly from the copyright holder.
To view a copy of this license, visit https://creativecommons.org/licenses/by/4.0/

Reviewer #1

This is an excellent study that addresses a fundamental question in nucleic acid chemistry: how backbone structure influences the evolvability of catalytic nucleic acids. The authors pose a clear and compelling question, whether conformational restriction in nucleic acid backbones, exemplified here by the nucleic acid mimic TNA, imposes constraints on the emergence and optimisation of catalytic function.

Using established in vitro selection methodologies, the authors successfully isolate two TNA enzymes capable of cleaving the same substrate as the well-characterised 10–23 DNAzyme. Through kinetic and temperature-dependent analyses, they demonstrate that these TNA enzymes exhibit enhanced catalytic activity at elevated temperatures. This behaviour is interpreted as reflecting a higher energetic barrier to formation of a productive active site in TNA relative to DNA. Importantly, the authors further show that variants with increased active site flexibility display reduced temperature dependence, providing a coherent mechanistic link between backbone rigidity, conformational dynamics, and catalytic efficiency.

As far as I can see, the methodology is sound and consistent with current standards in the field. The use of in vitro selection, combined with kinetic and temperature-dependent assays, is appropriate for addressing the stated research question. The comparison to a classic DNAzyme system further strengthens the study design.

Response: We thank the reviewer for their positive assessment of the study.

Some minor points:

1. Could the increased activity at higher temperature reflect effects on substrate binding, metal ion coordination, or global folding equilibria rather than solely active site formation. A greater discussion of these alternative contributions would improve balance and clarity and would not detract from the overall conclusions.

Response: The text was modified on page 9 to include other factors that may have contributed to increased threozyme activity at higher temperature.

2. I also wonder what would have happened if the authors had attempted the selection experiments conditions at 50 and not 37 degC? Perhaps not possible, but did the authors try this?

Response: This is a very interesting question and one that speaks to how such selections could be performed in the future. While the answer to this question is unknown, and beyond the scope of the current study, it would be interesting to see if repeating the selection at 50 rather than 37C changes the outcome. Since the original selection oversampled the theoretical space available to the library, our initial feeling is that the results would be the same. Perhaps this question could be answered in the future once it is determined that the selection is still operational at the higher temperature.

Given the growing interest in xeno nucleic acids and their implications for synthetic biology and origins-of-life research, this work is both significant and timely. Excellent work, well done.

Reviewer #2

The authors have used in vitro selection to identify two TNAzymes capable of hydrolyzing RNA substrates. Interestingly, despite being selected at 37°C, both TNAzymes were poor catalysts at this temperature. Catalysis was much improved when the temperature was increased to 50°C. To shed light into this discrepancy, the authors investigated the temperature dependence of other TNAzymes as well as one of the most efficient DNAzymes (Dz10-23). This analysis revealed that Dz10-23 displayed an opposite tendency, i.e. performed well at 37°C but not at 50°C, while the other TNAzymes displayed an intermediate behaviour since they catalyzed RNA hydrolysis at both temperatures. The authors conclude that both the effect of conformationally restrained backbone of TNA and the nature of the active site dictated the efficiency of nucleic acid catalysts. All experiments were carried out in a competent manner, the manuscript is very well-written and clear, and the supporting information is of good quality. This manuscript addresses a very interesting question and of fundamental importance to derive more potent nucleic acid-based catalysts. However, the extent of the effects of the rigidity of the backbone and that of the conformation of the active site are difficult to separate and quantify. In addition, the conclusions of this manuscript rely on a very limited set of different sugar chemistries and substrates (see comments below). Hence, this manuscript could be considered for publication in Nature Communications if additional data are provided and the following revisions are considered:

- The authors should evaluate the temperature dependence of Dz10-23 with a more flexible active site. Temperature dependence might be identical to that observed with the less flexible active site but will allow for a direct comparison with Tz6-29 and Tz13-38 in Figure 4.

Response: This experiment is not straightforward because the active site architecture of each catalyst is an evolved property of that specific sequence. Dz10-23 requires base pairing interactions adjacent to the cleavage site for activity, whereas Tz6-29 and Tz13-38 were isolated from selections that yielded more flexible active site regions. Thus, simply removing base pairs from Dz10-23 or introducing new base pairs into threozymes would likely abrogate activity rather than provide a controlled test of active site flexibility. For this reason, we do not view such designs as a viable experiment with an interpretable outcome.

- The authors should also evaluate the temperature dependence of nucleic acid enzymes with other backbones including small ribozymes cleaving KRAS substrates (see e.g. 10.1016/j.chembiol.2026.03.001; 10.1054/bjoc.2000.1363), FANAzymes (e.g. 10.1038/s41557-022-01021-z), LNAzyme (e.g. 10.1039/d5ra00161g) and HNAzyme (10.1038/nature13982) which will have different degrees of conformational flexibility. These experiments are important to further confirm the hypothesis of a strong effect of backbone structure on catalysis.

Response: We agree that comparing the temperature dependence of nucleic acid enzymes across different classes of genetic polymers would provide valuable insight into the relationship between backbone structure, conformational flexibility, and catalysis. However, we respectfully feel that such experiments are beyond the scope of the current study, and better suited as the subject of a grant proposal aimed at systematically exploring this problem under comparable conditions. We have revised the discussion on pages 14 and 15 to clearly state that a systematic comparison across additional XNA systems represents an important future direction.

- The authors should evaluate whether increasing the temperature to 50°C during the in vitro selection protocol would yield better catalysts against this target RNA. Temperature of in vitro selection has been shown to be an important parameter (10.1093/nar/gkh1007).

Response: The manuscript was updated on page 11 to include the suggested reference as an example of an RNA-cleaving DNAzyme with a temperature optimum near ambient conditions. However, we respectfully feel that repeating the selection at 50C is beyond the scope of the current study, and it is also unclear if such an experiment could be performed at that temperature.

- The authors may want to nuance their conclusions in the discussion section because besides backbone structure and flexibility of the active site, the presence of modifications on the nucleobase can change the outcome of a selection experiment and the temperature dependence (see e.g. 10.1002/cbic.202100600; 10.1039/C5MB00102A; 10.1093/nar/gkn1070).

Response: The manuscript was revised on page 15 to clarify that base modifications could impact the temperature dependence of a nucleic acid enzyme. References 51-53 were added.

- The observed first-order rate constants of all catalysts should be provided in the main manuscript.

Response: The manuscript was updated to include first-order rate constants with a summary of the data compiled in Supplementary Table 4.

Reviewer #3

The manuscript, "Dynamism and Evolvability in Nucleic Acid Enzymes," examines how backbone chemistry may influence the catalytic behavior of nucleic acid enzymes. The authors compare the well-known 10-23 DNAzyme with newly selected TNA-based RNA-cleaving enzymes, or threozymes, acting on a related RNA substrate. The main finding is that 10-23 DNAzyme is active at 37°C but loses much of its activity at 50°C, whereas the selected threozymes show weak activity at 37°C but substantially enhanced cleavage at 50°C. The authors further compare these enzymes with previously reported threozymes that contain more flexible active-site architectures. Overall, the work addresses an interesting question at the interface of nucleic acid chemistry, XNA biology, catalysis, and molecular evolution. The manuscript is clearly written, and the DNA-display selection strategy is technically interesting.

However, I think the manuscript requires major revision before publication. The main concern is that the conceptual interpretation—linking TNA backbone rigidity to catalytic dynamism and evolvability—is broader than what the current data can fully support. The temperature-dependent activity difference is interesting, but additional experimental and quantitative support would make the conclusions more convincing.

1. The key experiments should be repeated with stronger statistical support: Many central experiments, including the activity screens, kinetic curves, melting analyses, and nuclease-resistance assays, are reported with n = 2. Given that the manuscript makes broad mechanistic claims about temperature-dependent catalysis, conformational constraints, and evolvability, the key quantitative experiments should ideally be repeated with at least n = 3 independent replicates, with appropriate error analysis. This is especially important for Figures 3 and 4 and the associated supplementary figures.

Response: All key experiments were updated to include data for three independent replicates (see main text Figures 3 and 4, and Supplementary Figures S2, S3, S5, S8, S9, S11, and S12).

2. The comparison between DNAzyme and threozymes should be better controlled or more cautiously interpreted: The DNAzyme and threozyme reactions are performed under substantially different conditions. For example, the DNAzyme reactions use 2 mM $MgCl_2$, 25 nM enzyme, and

250 nM substrate, whereas the threozyme reactions use 20 mM $MgCl_2$, 500 nM enzyme, and 250 nM substrate. These differences may be necessary, but they complicate direct comparison of catalytic performance and temperature dependence. The authors should either provide matched-condition experiments, where feasible, particularly enzyme/substrate settings, or more explicitly explain the rationale and limitations of the comparison.

Response: We agree that a direct comparison between the DNAzyme and threozyme systems is complicated by differences in the conditions used for each catalyst. The reaction conditions were chosen to reflect the optimal or near-optimal conditions for each enzyme, which differ substantially in their Mg^{2+} requirement and turnover ability. As noted by the reviewer, the DNAzyme reactions were performed using 2 mM $MgCl_2$ and 25 nM enzyme, whereas the threozyme reactions required higher Mg^{2+} and catalyst concentrations to achieve measurable activity. The manuscript was revised on page 11 to clarify this difference more explicitly.

3. The mechanistic claim about a higher free-energy barrier should be softened or supported by additional analysis: The data clearly show that the selected threozymes are more active at elevated temperature. However, the conclusion that TNA catalysis "must overcome a higher free-energy barrier" is stronger than the current evidence allows. The authors could strengthen this point by reporting apparent rate constants over a temperature range and, if possible, performing Arrhenius or Eyring-type analysis. Without such analysis, the manuscript should describe the results more cautiously as temperature-dependent activation rather than direct evidence for a defined energetic barrier.

Response: The manuscript was revised on page 11 to include new data for a two-point Arrhenius analysis for all enzymes (see new data in Supplementary Table 4). Collectively, these results demonstrate that the newly identified threozymes exhibit a fundamentally different temperature dependence from both Dz 10-23 and previously characterized threozymes, with catalytic activity increasing rather than decreasing with elevated temperature. This behavior is consistent with a model in which catalysis by the newly selected threozymes requires thermal activation to access productive conformational states.

4. The term "evolvability" should be used more carefully: The manuscript is framed around dynamism and evolvability, but evolvability itself is not directly measured. The study identifies TNA catalysts and compares their temperature-dependent activity, but it does not directly assess adaptive trajectories, mutational robustness, or the accessible diversity of catalytic solutions relative to DNA under parallel selection conditions. The authors may retain evolvability as a broader conceptual framework, but statements suggesting that the study establishes fundamental limits on TNA evolvability should be moderated.

Response: The central thesis of our manuscript is that the backbone architecture of a genetic polymer is an important determinant of catalytic evolvability. Throughout the text, we have made every effort to frame this problem using words, like 'may', 'could', and 'suggest' to indicate that further research is needed to better understand how backbone structure influences the evolvability of catalytic nucleic acids. However, we do respectfully feel that our use of the term evolvability is reasonable given the scope of the manuscript and the data provided. It also provides ample room for future studies that can explore a range of XNA systems (including DNA and RNA) under identical selection conditions.

5. The relationship between selection conditions and post-selection activity deserves clarification: The threozyme selections were performed at 37°C, yet the best selected threozymes show much stronger activity at 50°C in the trans-cleavage assays. This is intriguing but should be discussed more clearly. Are the selected catalysts more active in the original DNA-display format at 37°C

than in the trans assay? Could the display architecture stabilize a productive conformation? Clarifying this point would help readers understand why selection at 37°C yielded enzymes that appear to function best at elevated temperature.

Response: The question of why threozymes selected at 37C function better at 50C is interesting and certainly the basis of the current manuscript. Since the selection oversampled the starting library, we believe that repeating the selection at 50C would yield similar results. In this light, the best answer to the question is that the top performing sequences were 'good enough' to survive a lower temperature selection where their activity was suppressed but not abrogated.

We feel that a detailed analysis of the display strategy is beyond the scope of the current study, but could be addressed in a future study using a more traditional two-enzyme selection strategy to transcribe and reverse transcribe TNA information back and forth between TNA and DNA.

6. The biological relevance should be presented cautiously: The nuclease-resistance experiments are useful and support the known biostability of TNA. However, the newly selected threozymes require elevated temperature and high Mg^{2+} concentration for strong activity. The manuscript should therefore distinguish more clearly between the conceptual importance of these catalysts and their immediate practical applicability in biological environments.

Response: We do not feel that this is a valid concern since this is not a key part of the paper and the experiments were a standard part of nucleic acid characterization.

7. Writing and presentation issues: The manuscript is generally well written, but several minor issues should be corrected. For example, "randoms TNA sequences" should be changed to "random TNA sequences"; "a only limited set" should be "only a limited set"; "It's worth noting" should be changed to "It is worth noting"; "Noticeably" should be changed to "Notably". The authors should also define what the error bars represent in all relevant figure legends.

Response: The manuscript was revised to correct the mistakes.

In summary, this is an interesting and potentially important study that raises a meaningful question about how nucleic acid backbone chemistry influences catalysis. I support publication after major revision, provided that the authors strengthen the statistical and kinetic support for the key findings, clarify the basis of the DNAzyme–threozyme comparison, and moderate the broader claims about free-energy barriers and evolvability.
