# Supplementary material for: Dynamism and evolvability in nucleic acid enzymes
Source: Nat Commun. 2026 Aug 14;17:9757. doi: 10.1038/s41467-026-76473-9 (PMC13572484; doi:10.1038/s41467-026-76473-9)

Source Data for
Main Text and SI Figures

(graph data and uncropped gels)

Dynamism and Evolvability in Nucleic Acid Enzymes

Erica M. Lee[1] and John C. Chaput[1-4]*

[1]Department of Pharmaceutical Sciences, University of California, Irvine, CA 92697-3958 USA
[2]Department of Chemistry, University of California, Irvine, CA 92697-3958 USA
[3]Department of Molecular Biology and Biochemistry, University of California, CA 92697-3958 USA
[4]Department of Chemical and Biomolecular Engineering, University of California, Irvine, CA 92697-3958 USA

Figure 3a. Activity screen of chemically synthesized hits from round 8.

Rep #1	Sample	Product (sign	Total (signal)	% Cleavage
37˚C	N15-1	450	16450	2.73556231
	N15-2	141	17441	0.80843988
	N15-3	50	18650	0.26809651
	N15-4	53	23653	0.22407306
	N15-5	44.5	22244.5	0.20004945
	N15-6	79.8	21779.8	0.36639455
	N20-1	1060	13460	7.87518574
	N20-2	172	18272	0.941331
	N20-3	51.9	26651.9	0.19473283
	N20-4	83.6	27283.6	0.30641118
	N20-5	7.19	24707.19	0.02910084
	N20-6	2.65	25402.65	0.01043198

Rep #2	Sample	Product (sign	Total (signal)	% Cleavage
37˚C	N15-1	583	22783	2.55892551
	N15-2	124	16924	0.73268731
	N15-3	68.8	21668.8	0.3175072
	N15-4	73.5	26573.5	0.27659134
	N15-5	30.4	16930.4	0.17955866
	N15-6	63.5	18463.5	0.34392179
	N20-1	1340	16540	8.10157195
	N20-2	176	20276	0.86802131
	N20-3	44.3	23944.3	0.18501272
	N20-4	90.2	32390.2	0.27847929
	N20-5	13.6	24413.6	0.05570666
	N20-6	4.29	23004.29	0.0186487

Rep #3	Sample	Product (sign	Total (signal)	% Cleavage
37˚C	N15-1	398	13798	2.88447601
	N15-2	97	15597	0.62191447
	N15-3	34.5	18434.5	0.1871491
	N15-4	52.4	26652.4	0.19660518
	N15-5	6.63	16706.63	0.03968484
	N15-6	10.5	13310.5	0.07888509
	N20-1	762	8602	8.85840502
	N20-2	21.3	3991.3	0.53366071
	N20-3	3.58	17003.58	0.02105439
	N20-4	25	19925	0.12547051
	N20-5	2.63	9572.63	0.02747416
	N20-6	1.38	24101.38	0.00572581

AVERAGE	Sample	% Cleavage	STDEV
37˚C	N15-1	2.72632128	0.16297187
	N15-2	0.72101389	0.09380903
	N15-3	0.25758427	0.06581177
	N15-4	0.23242319	0.0406416
	N15-5	0.13976432	0.08727482
	N15-6	0.26306714	0.15990162
	N20-1	8.27838757	0.51490568
	N20-2	0.78100434	0.21731942
	N20-3	0.13359998	0.09758843
	N20-4	0.23678699	0.09740927
	N20-5	0.03742722	0.01585134
	N20-6	0.01160216	0.00654043

Rep #1	Sample	Product (sign	Total (signal)	% Cleavage
50˚C	N15-1	9780	14920	65.5495979
	N15-2	2870	18370	15.6232989
	N15-3	2020	20920	9.65583174
	N15-4	1580	22880	6.90559441
	N15-5	1180	21080	5.59772296
	N15-6	1290	19290	6.6874028
	N20-1	15900	18240	87.1710526
	N20-2	1800	18600	9.67741935
	N20-3	456	21156	2.1554169
	N20-4	1760	20660	8.51887706
	N20-5	472	19872	2.37520129
	N20-6	465	24365	1.90847527

Rep #2	Sample	Product (sign	Total (signal)	% Cleavage
50˚C	N15-1	14200	19270	73.6896731
	N15-2	2120	14120	15.0141643
	N15-3	2300	21600	10.6481481
	N15-4	1280	17280	7.40740741
	N15-5	1040	17940	5.79710145
	N15-6	1450	18750	7.73333333
	N20-1	19300	22190	86.9761154
	N20-2	1540	15940	9.66122961
	N20-3	373	15773	2.36480061
	N20-4	1800	18800	9.57446809
	N20-5	367	14767	2.48527121
	N20-6	387	18187	2.12789355

Rep #3	Sample	Product (sign	Total (signal)	% Cleavage
50˚C	N15-1	9590	12600	76.1111111
	N15-2	1440	10140	14.2011834
	N15-3	1310	13910	9.41768512
	N15-4	972	10042	9.67934674
	N15-5	824	14524	5.67336822
	N15-6	668	9038	7.39101571
	N20-1	16400	19280	85.0622407
	N20-2	1030	15730	6.54799746
	N20-3	531	19431	2.73274664
	N20-4	2010	19710	10.1978691
	N20-5	405	18005	2.24937517
	N20-6	472	24572	1.92088556

AVERAGE	Sample	% Cleavage	STDEV
50˚C	N15-1	71.7834607	5.53277732
	N15-2	14.9462155	0.71348851
	N15-3	9.90722167	0.65261588
	N15-4	7.99744952	1.47801819
	N15-5	5.68939754	0.10065113
	N15-6	7.27058395	0.53326403
	N20-1	86.4031362	1.16533292
	N20-2	8.62888214	1.80211718
	N20-3	2.41765472	0.29227141
	N20-4	9.43040475	0.84871624
	N20-5	2.36994922	0.11803569
	N20-6	1.98575146	0.12325496

Figure 3b-c. Kinetic assays of Tz N15-1 and Tz N20-1.

N15-1	Rep #1	Time (h)	Product (sign	Total (signal)	% Cleavage	Rep #2	Time (h)	Product (sign	Total (signal)	% Cleavage	Rep #3	Time (h)	Product (sign	Total (signal)	% Cleavage	Average	Time (h)	% Cleavage	STDEV
	37˚C	0	6.46	30906.46	0.02090178	37˚C	0	6.59	29806.59	0.0221092	37˚C	0	3.04	24103.04	0.01261252		0	0.01854117	0.00516973
		1	51.1	26451.1	0.19318667		1	52.4	23252.4	0.22535308		1	28.8	18128.8	0.15886325		1	0.19246767	0.03325075
		2	87.3	25987.3	0.33593332		2	91.9	24791.9	0.37068559		2	60.6	15260.6	0.39710103		2	0.36790665	0.0306784
		3	122	25022	0.48757094		3	115	22315	0.51534842		3	93.5	15593.5	0.59960881		3	0.53417606	0.05834365
		4	137	22637	0.60520387		4	230	32230	0.71362085		4	130	16130	0.80595164		4	0.70825879	0.10048125
		8	238	22538	1.05599432		8	250	20850	1.19904077		8	222	14922	1.48773623		8	1.24759044	0.21992742
		12	310	19210	1.61374284		12	361	20761	1.73883724		12	337	14537	2.31822247		12	1.89026752	0.37586065
		24	678	22578	3.0029232		24	801	24701	3.24278369		24	584	14984	3.89749066		24	3.38106585	0.46303801

	Rep #1	Time (h)	Product (sign	Total (signal)	% Cleavage	Rep #2	Time (h)	Product (sign	Total (signal)	% Cleavage	Rep #3	Time (h)	Product (sign	Total (signal)	% Cleavage	Average	Time (h)	% Cleavage	STDEV
	50˚C	0	2.73	28802.73	0.00947827	50˚C	0	3.21	27903.21	0.01150405	50˚C	0	6.03	22506.03	0.02679282		0	0.01592505	0.00946611
		1	1480	25980	5.69668976		1	1320	24020	5.49542048		1	1090	15990	6.81676048		1	6.00295691	0.71192311
		2	2770	25170	11.0051649		2	2420	21920	11.040146		2	1990	15990	12.4452783		2	11.4968631	0.8215379
		3	4280	26780	15.9820762		3	3760	24360	15.4351396		3	2810	15410	18.2349124		3	16.5507094	1.48397768
		4	4990	23690	21.06374		4	6030	27330	22.0636663		4	3760	15860	23.7074401		4	22.2782821	1.33485301
		8	9800	26000	37.6923077		8	8720	23420	37.2331341		8	6300	15090	41.749503		8	38.8916482	2.4856006
		12	12900	25400	50.7874016		12	13300	25700	51.7509728		12	9150	16370	55.8949297		12	52.8111014	2.71378251
		24	20700	27030	76.581576		24	27800	34710	80.0921925		24	14000	17380	80.552359		24	79.0753758	2.17191539

N20-1	Rep #1	Time (h)	Product (sign	Total (signal)	% Cleavage	Rep #2	Time (h)	Product (sign	Total (signal)	% Cleavage	Rep #3	Time (h)	Product (sign	Total (signal)	% Cleavage	Average	Time (h)	% Cleavage	STDEV
	37˚C	0	5.27	28505.27	0.01848781	37˚C	0	9.55	30509.55	0.03130167	37˚C	0	4.8	21504.8	0.0223206		0	0.02403669	0.00657705
		1	124	25224	0.49159531		1	117	23017	0.50831994		1	127	18627	0.68180598		1	0.56057374	0.1053227
		2	208	24208	0.85922009		2	224	24724	0.90600227		2	210	16310	1.28755365		2	1.017592	0.23496087
		3	299	24699	1.21057533		3	276	21776	1.2674504		3	320	17220	1.8583043		3	1.44544334	0.35867718
		4	437	28237	1.54761483		4	396	24596	1.61001789		4	395	16195	2.43902439		4	1.86555237	0.49762049
		8	667	24367	2.73730866		8	548	18248	3.00306883		8	646	12746	5.06825671		8	3.60287806	1.27599296
		12	800	19300	4.14507772		12	870	20170	4.31333664		12	878	12378	7.09322992		12	5.18388143	1.6556831
		24	1740	22740	7.65171504		24	1810	22310	8.11295383		24	1630	13830	11.7859725		24	9.18354713	2.26553498

	Rep #1	Time (h)	Product (sign	Total (signal)	% Cleavage	Rep #2	Time (h)	Product (sign	Total (signal)	% Cleavage	Rep #3	Time (h)	Product (sign	Total (signal)	% Cleavage	Average	Time (h)	% Cleavage	STDEV
	50˚C	0	11.5	26811.5	0.04289204	50˚C	0	11.9	22511.9	0.05286093	50˚C	0	4.48	23704.48	0.01889938		0	0.03821745	0.01745668
		1	1720	25420	6.76632573		1	1720	24420	7.04340704		1	1780	20180	8.82061447		1	7.54344908	1.11470045
		2	3390	25690	13.195796		2	3360	23960	14.0233723		2	3420	20520	16.6666667		2	14.6286117	1.81286287
		3	5130	26530	19.3366001		3	4750	23450	20.2558635		3	5110	21610	23.64646		3	21.0796412	2.26995161
		4	6080	24680	24.6353323		4	5430	19730	27.5215408		4	6440	21240	30.3201507		4	27.4923412	2.84252169
		8	11600	25400	45.6692913		8	9010	19610	45.9459459		8	10500	19840	52.9233871		8	48.1795415	4.11061893
		12	14700	24520	59.9510604		12	13100	20950	62.5298329		12	15100	22080	68.3876812		12	63.6228582	4.32321301
		24	23000	26550	86.6290019		24	19200	21850	87.8718535		24	20100	21920	91.6970803		24	88.7326452	2.64141561

Figure 4a. Kinetic assays of Dz 10-23 (multiple turnover).

Dz 10-23	Rep #1	Time (min)	Product (sign	Total (signal)	% Cleavage		Rep #2	Time (min)	Product (sign	Total (signal)	% Cleavage		Rep #3	Time (min)	Product (sign	Total (signal)	% Cleavage		Average	Time (min)	% Cleavage	STDEV
	37˚C	0	292	32992	0.88506305		37˚C	0	228	25228	0.90375773		37˚C	0	200	38300	0.52219321			0	0.770338	0.21510288
		30	9770	33570	29.1033661			30	6680	19680	33.9430894			30	10900	34200	31.871345			30	31.6392669	2.42819391
		60	13200	28200	46.8085106			60	11800	21750	54.2528736			60	16700	32100	52.0249221			60	51.0287688	3.82084754
		90	18200	31000	58.7096774			90	14800	22290	66.3974877			90	22200	35000	63.4285714			90	62.8452455	3.87695866
		120	22100	33000	66.969697			120	15800	21440	73.6940299			120	24400	34220	71.3033314			120	70.6556861	3.40862825
		150	23900	32520	73.4932349			150	16200	20430	79.2951542			150	25700	33220	77.3630343			150	76.7171411	2.95439507
	Rep #1	Time (min)	Product (sign	Total (signal)	% Cleavage		Rep #2	Time (min)	Product (sign	Total (signal)	% Cleavage		Rep #3	Time (min)	Product (sign	Total (signal)	% Cleavage		Average	Time (min)	% Cleavage	STDEV
	50˚C	0	320	35020	0.91376356		50˚C	0	220	23320	0.94339623		50˚C	0	148	33648	0.43984784			0	0.76566921	0.28255831
		30	953	34353	2.77413909			30	663	17363	3.81846455			30	392	29192	1.34283365			30	2.64514576	1.24284615
		60	1110	31610	3.51154698			60	1440	23940	6.01503759			60	301	19601	1.53563594			60	3.68740684	2.24487301
		90	1450	31450	4.61049285			90	1970	20770	9.4848339			90	506	26806	1.8876371			90	5.32765461	3.84903761
		120	1880	32480	5.78817734			120	2480	23580	10.5173876			120	565	27265	2.07225381			120	6.12593959	4.23268635
		150	2830	31630	8.94720202			150	3110	22110	14.0660335			150	553	27753	1.99257738			150	8.33527096	6.05994474

Figure 4b-c. Kinetic assays of Tz 6-29 and Tz 13-38.

6-29	Rep #1	Time (h)	Product (sign	Total (signal)	% Cleavage		Rep #2	Time (h)	Product (sign	Total (signal)	% Cleavage		Rep #3	Time (h)	Product (sign	Total (signal)	% Cleavage		Average	Time (h)	% Cleavage	STDEV
	37˚C	0	6.76	433.76	1.55846551		37˚C	0	4.93	397.93	1.23891137		37˚C	0	1.35	473.35	0.28520123			0	1.02752603	0.66242985
		1	29	494	5.87044534			1	25.4	415.4	6.11458835			1	47.7	703.7	6.77845673			1	6.25449681	0.46989568
		2	27.8	207.8	13.3782483			2	36.3	305.3	11.8899443			2	86.8	561.8	15.4503382			2	13.5728436	1.78815593
		3	42.4	248.4	17.0692432			3	49.9	294.9	16.9209902			3	128	574	22.2996516			3	18.763295	3.0634716
		4	75.9	340.9	22.2645937			4	97.2	467.2	20.8047945			4	142	574	24.738676			4	22.6026881	1.98861425
		8	53.5	138.1	38.7400434			8	61.1	177.1	34.5002823			8	177	485	36.4948454			8	36.5783904	2.1211149
		12	58.8	115	51.1304348			12	66.4	137.8	48.1857765			12	83.3	167.6	49.7016706			12	49.6726273	1.47254397
		24	100	136.6	73.2064422			24	85.2	117	72.8205128			24	95	142.4	66.7134831			24	70.9134794	3.64241838
	Rep #1	Time (h)	Product (sign	Total (signal)	% Cleavage		Rep #2	Time (h)	Product (sign	Total (signal)	% Cleavage		Rep #3	Time (h)	Product (sign	Total (signal)	% Cleavage		Average	Time (h)	% Cleavage	STDEV
	50˚C	0	4.44	744.44	0.59642147		50˚C	0	7.16	575.16	1.24487099		50˚C	0	0.119	550.119	0.02163168			0	0.62097472	0.61198917
		1	41.1	437.1	9.40288264			1	33.4	307.4	10.8653221			1	35.7	618.7	5.77016325			1	8.67945598	2.6234844
		2	86.2	500.2	17.2331068			2	64.7	405.7	15.9477446			2	57.8	462.8	12.4891962			2	15.2233492	2.4535145
		3	125	551	22.6860254			3	101	479	21.085595			3	83.1	484.1	17.1658748			3	20.3124984	2.84011873
		4	150	571	26.2697023			4	215	737	29.1723202			4	105	522	20.1149425			4	25.185655	4.62497476
		8	219	410	53.4146341			8	186	388	47.9381443			8	129	353	36.5439093			8	45.9655626	8.60660489
		12	248	373	66.4879357			12	268	437	61.3272311			12	112	190.2	58.8853838			12	62.2335169	3.88145772
		24	328	401.9	81.6123414			24	377	464.6	81.145071			24	155	192.3	80.6032241			24	81.1202122	0.5050177
13-38	Rep #1	Time (h)	Product (sign	Total (signal)	% Cleavage		Rep #2	Time (h)	Product (sign	Total (signal)	% Cleavage		Rep #3	Time (h)	Product (sign	Total (signal)	% Cleavage		Average	Time (h)	% Cleavage	STDEV
	37˚C	0	3.45	521.45	0.66161665		37˚C	0	4.84	532.84	0.90834021		37˚C	0	0.632	419.632	0.15060815			0	0.57352167	0.38647123
		1	58.2	332.2	17.5195665			1	152	578	26.2975779			1	85.3	320.3	26.6312832			1	23.4828092	5.16701433
		2	102	352	28.9772727			2	218	568	38.3802817			2	98.2	286.2	34.3116702			2	33.8897415	4.71568258
		3	156	356	43.8202247			3	290	617	47.0016207			3	110	288	38.1944444			3	43.00543	4.45976529
		4	218	426	51.1737089			4	316	602	52.4916944			4	119	280	42.5			4	48.7218011	5.42838632
		8	144	206.2	69.8351115			8	268	364.4	73.5455543			8	131	201.4	65.0446872			8	69.4751177	4.26185197
		12	115	154	74.6753247			12	237	297.6	79.6370968			12	48.9	73	66.9863014			12	73.7662409	6.37420434
		24	104	133	78.1954887			24	122	150.4	81.1170213			24	55.4	71.9	77.0514604			24	78.7879901	2.09654235
	Rep #1	Time (h)	Product (sign	Total (signal)	% Cleavage		Rep #2	Time (h)	Product (sign	Total (signal)	% Cleavage		Rep #3	Time (h)	Product (sign	Total (signal)	% Cleavage		Average	Time (h)	% Cleavage	STDEV
	50˚C	0	5.63	724.63	0.77694824		50˚C	0	7.01	882.01	0.79477557		50˚C	0	0.229	755.229	0.03032193			0	0.53401524	0.43630227
		1	24.3	215.3	11.2865769			1	43.9	355.9	12.3349255			1	36.6	307.6	11.8985696			1	11.840024	0.52662076
		2	54.5	277.5	19.6396396			2	74.4	347.4	21.4162349			2	63.5	324.5	19.568567			2	20.2081472	1.04683798
		3	80.9	321.9	25.1320286			3	101	351	28.7749288			3	92.6	358.6	25.8226436			3	26.5765337	1.93492716
		4	98	326	30.0613497			4	127	393	32.3155216			4	115	379	30.3430079			4	30.9066264	1.22823945
		8	159	340	46.7647059			8	150	327	45.8715596			8	168	328	51.2195122			8	47.9519259	2.86483292
		12	213	368	57.8804348			12	195	344	56.6860465			12	187	290	64.4827586			12	59.68308	4.19932461
		24	307	421	72.9216152			24	274	380	72.1052632			24	240	308	77.9220779			24	74.3163188	3.14924309

SI Figure 5a-b. Magnesium dependence.

Rep #1	Mg2+	Sample	Product (sign	Total (signal)	% Cleavage
	0 mM	RNA	13.3	24313.3	0.05470257
		N15-1	4.34	31604.34	0.01373229
		N20-1	1.85	30101.85	0.0061458
	1 mM	RNA	12.9	26412.9	0.04883977
		N15-1	1280	27180	4.70934511
		N20-1	1410	28610	4.92834673
	5 mM	RNA	118	16418	0.71872335
		N15-1	8290	24690	33.5763467
		N20-1	8720	21420	40.7096172
	10 mM	RNA	187	14587	1.28196339
		N15-1	10900	22800	47.8070175
		N20-1	13400	23800	56.302521
	20 mM	RNA	18.5	9398.5	0.19683992
		N15-1	9390	16670	56.3287343
		N20-1	12400	16880	73.4597156

Rep #2	Mg2+	Sample	Product (sign	Total (signal)	% Cleavage
	0 mM	RNA	1.53	25701.53	0.00595295
		N15-1	1.88	31901.88	0.00589307
		N20-1	1.18	29001.18	0.0040688
	1 mM	RNA	12.8	24512.8	0.05221762
		N15-1	1020	26520	3.84615385
		N20-1	1150	25550	4.50097847
	5 mM	RNA	84.1	13384.1	0.62835753
		N15-1	4770	16270	29.3177628
		N20-1	7740	18340	42.2028353
	10 mM	RNA	148	12948	1.14303367
		N15-1	8950	21650	41.3394919
		N20-1	8430	15990	52.7204503
	20 mM	RNA	32.1	5692.1	0.5639395
		N15-1	10500	16450	63.8297872
		N20-1	13100	17710	73.9695088

Rep #3	Mg2+	Sample	Product (sign	Total (signal)	% Cleavage
	0 mM	RNA	10.3	21710.3	0.04744292
		N15-1	7.93	30607.93	0.02590832
		N20-1	6.43	37106.43	0.01732853
	1 mM	RNA	-17.2	25082.8	0
		N15-1	1250	29350	4.25894378
		N20-1	1900	34000	5.58823529
	5 mM	RNA	-1.86	15098.14	0
		N15-1	7460	23260	32.072227
		N20-1	12400	28200	43.9716312
	10 mM	RNA	-28.1	16271.9	0
		N15-1	11300	24300	46.5020576
		N20-1	14700	23280	63.1443299
	20 mM	RNA	-41.3	8318.7	0
		N15-1	15000	25300	59.2885375
		N20-1	16600	21440	77.4253731

Average	Sample	Mg2+ mM	% Cleavage	STDEV
	RNA	0	0.03603281	0.0263016
		1	0.0336858	0.0292216
		5	0.44902696	0.39148487
		10	0.80833236	0.70347442
		20	0.25359314	0.2862213
	N15-1	0	0.01517789	0.01008563
		1	4.27148091	0.43173218
		5	31.6554455	2.15966767
		10	45.216189	3.42013384
		20	59.8156863	3.77820901
	N20-1	0	0.00918104	0.00713195
		1	5.0058535	0.54775663
		5	42.2946946	1.63294595
		10	57.3891004	5.29620691
		20	74.9515325	2.15751891

SI Figure 6a. Activity of Dz 10-23 pre-treated with TURBO DNase.

Replicate	Sample	Time (h)	TURBO	Product (sign	Total (signal)	% Cleavage
#1	Dz 10-23	30	-	5720	17920	31.9196429
			+	19.4	4489.4	0.43212902
		60	-	9100	18140	50.1653804
			+	28	4318	0.64844836

Replicate	Sample	Time (h)	TURBO	Product (sign	Total (signal)	% Cleavage
#2	Dz 10-23	30	-	1140	4430	25.7336343
			+	15.3	1515.3	1.00970105
		60	-	576	1796	32.0712695
			+	15.7	1175.7	1.33537467

Average	Sample	Time (h)	TURBO	% Cleavage	STDEV
Rep #1-2	Dz 10-23	30	-	28.8266386	4.37416859
			+	0.72091503	0.4084051
		60	-	41.1183249	12.7944685
			+	0.99191151	0.48573026

SI Figure 6b-c. Activity of Tz N15-1 and Tz N20-1 pre-treated with TURBO DNase.

Replicate	Sample	Time (h)	TURBO	Product (sign	Total (signal)	% Cleavage
#1	N15-1	6	-	1350	6510	20.7373272
			+	2050	10410	19.6926033
		18	-	3120	6310	49.4453249
			+	4410	8640	51.0416667
	N20-1	6	-	3090	11630	26.5692175
			+	2270	8380	27.0883055
		18	-	6780	10810	62.719704
			+	5410	8610	62.8339141

Replicate	Sample	Time (h)	TURBO	Product (sign	Total (signal)	% Cleavage
#2	N15-1	6	-	845	3855	21.919585
			+	1370	6580	20.8206687
		18	-	2720	5310	51.2241055
			+	3850	7720	49.8704663
	N20-1	6	-	1760	6900	25.5072464
			+	1670	6200	26.9354839
		18	-	4960	8430	58.8374852
			+	4470	7430	60.1615074

Average	Sample	Time (h)	TURBO	% Cleavage	STDEV
Rep #1-2	N15-1	6	-	21.3284561	0.83598248
			+	20.256636	0.79766271
		18	-	50.3347152	1.25778781
			+	50.4560665	0.82816371
	N20-1	6	-	26.038232	0.75092701
			+	27.0118947	0.1080612
		18	-	60.7785946	2.74514324
			+	61.4977107	1.88967687

SI Figure 9. Kinetic assays of Dz 10-23 (single turnover).

Dz 10-23	37C #1	Time (min)	Product (sign	Total (signal)	% Cleavage		37C #2	Time (min)	Product (sign	Total (signal)	% Cleavage		37C #3	Time (min)	Product (sign	Total (signal)	% Cleavage		Average	Time (min)	% Cleavage	STDEV
		0	12.5	48212.5	0.02592689			0	11.3	48311.3	0.02338997			0	14.8	48314.8	0.03063244			0	0.02664976	0.00367495
		1	18000	52500	34.2857143			1	17300	52700	32.8273245			1	18800	56700	33.1569665			1	33.4233351	0.7648133
		2	23200	47000	49.3617021			2	21900	46300	47.300216			2	24900	51700	48.1624758			2	48.274798	1.03532289
		3	25700	42100	61.0451306			3	27900	46600	59.8712446			3	30000	49900	60.1202405			3	60.3455386	0.61852366
		4	33700	48900	68.9161554			4	31200	45600	68.4210526			4	35200	51800	67.953668			4	68.430292	0.48131025
		5	32600	43500	74.9425287			5	32100	43400	73.9631336			5	36700	49200	74.5934959			5	74.4997194	0.49638615
		7	36600	44680	81.915846			7	38400	46630	82.3504182			7	39700	48530	81.805069			7	82.0237777	0.28825053
		10	35600	40860	87.1267744			10	38100	43690	87.2053101			10	38700	44290	87.3786408			10	87.2369084	0.12887208
		15	36400	40240	90.4572565			15	37900	41960	90.3241182			15	39900	43900	90.8883827			15	90.5565858	0.2949548
		30	37700	40390	93.3399356			30	39700	42590	93.2143696			30	39800	42670	93.273963			30	93.2760894	0.06281003
		60	35000	37020	94.54349			60	38600	40750	94.7239264			60	41700	44150	94.4507361			60	94.5727175	0.13892053
	50C #1	Time (min)	Product (sign	Total (signal)	% Cleavage		50C #2	Time (min)	Product (sign	Total (signal)	% Cleavage		50C #3	Time (min)	Product (sign	Total (signal)	% Cleavage		Average	Time (min)	% Cleavage	STDEV
		0	16.8	57816.8	0.0290573			0	16.5	57616.5	0.02863763			0	17.7	48217.7	0.03670851			0	0.03146781	0.00454343
		1	3640	51340	7.08998831			1	3570	61670	5.78887628			1	3430	58830	5.83035866			1	6.23640775	0.73951337
		2	3550	47750	7.43455497			2	3310	51110	6.47622774			2	3070	49670	6.18079324			2	6.69719198	0.65543766
		3	3900	44700	8.72483221			3	3840	51740	7.421724			3	3400	47700	7.1278826			3	7.75814627	0.84996881
		4	4740	46940	10.0979974			4	4590	53190	8.62944162			4	3860	47760	8.08207705			4	8.93650537	1.04244898
		5	5190	47290	10.9748361			5	4980	53180	9.36442272			5	4730	52230	9.05609803			5	9.79845229	1.03037625
		7	5550	42050	13.1985731			7	5580	49080	11.3691932			7	5140	47340	10.8576257			7	11.808464	1.23074282
		10	6610	41910	15.7718921			10	6510	47910	13.5879775			10	6090	47190	12.9052765			10	14.088382	1.49738934
		15	9040	43940	20.5735093			15	8560	48660	17.5914509			15	8090	48490	16.6838523			15	18.2829375	2.03493813
		30	13800	41400	33.3333333			30	14400	50600	28.458498			30	13600	50800	26.7716535			30	29.5211616	3.40746986
		60	23200	44800	51.7857143			60	23800	51800	45.9459459			60	21700	48900	44.3762781			60	47.3693128	3.90440981

SI Figure 10.

Replicate #1 (Tm plots graphed with temperature vs normalized absorbance)								
Dz 10-23			N15-1			N20-1		
Temperature	Normalized A	Abs	Temperature	Normalized A	Abs	Temperature	Normalized A	Abs
19.9699993	0	0.44189712	19.9699993	0	0.48332843	20.1200008	0	0.51584041
21.0699997	0.00096853	0.44232512	21.1100006	0.00076416	0.48369777	21.2099991	0.00169949	0.51671708
21.9200001	0.00233908	0.44293076	22.1700001	0.0018291	0.48421249	22.2199993	0.00318267	0.51748216
22.9699993	0.0034425	0.44341835	23.1700001	0.00325395	0.48490116	23.2700005	0.00477944	0.51830584
24.0200005	0.00505968	0.44413298	24.1200008	0.00461209	0.48555759	24.2199993	0.00636708	0.51912481
25.0200005	0.00671808	0.44486582	25.1200008	0.0056597	0.48606393	25.2700005	0.00827386	0.5201084
25.9200001	0.008527	0.44566518	26.0400009	0.00708813	0.48675433	26.2199993	0.01015326	0.52107787
27.0200005	0.01009509	0.44635811	27.1200008	0.00851952	0.48744616	27.2700005	0.01240414	0.52223897
27.9699993	0.01195358	0.44717938	28.1200008	0.01010636	0.48821312	28.2199993	0.01426748	0.52320015
28.9699993	0.01408467	0.4481211	29.0699997	0.01147837	0.48887625	29.1700001	0.01648925	0.52434623
30.0200005	0.01625603	0.44908062	30.1700001	0.01309425	0.48965725	30.2199993	0.01869657	0.52548486
31.0200005	0.01855505	0.45009655	31.0699997	0.01490392	0.49053192	31.2199993	0.02154924	0.52695638
31.9899998	0.02109228	0.45121774	32.1699982	0.01632797	0.49122021	32.2200012	0.02389638	0.52816713
32.9599991	0.0236173	0.45233354	33.1199989	0.01831474	0.49218047	33.1699982	0.02640009	0.52945864
34	0.02643016	0.45357654	34.2200012	0.02049475	0.49323413	34.2700005	0.02946501	0.53103966
34.9900017	0.02933339	0.45485947	35.2200012	0.02222038	0.49406818	35.2200012	0.0327392	0.53272861
35.9700012	0.03227561	0.45615962	36.0699997	0.0241191	0.49498588	36.2200012	0.03557349	0.53419065
36.9700012	0.03592603	0.45777273	37.1199989	0.02621531	0.49599904	37.2700005	0.03930999	0.53611809
38.0200005	0.0390361	0.45914707	38.1699982	0.02840673	0.49705821	38.2200012	0.04279516	0.53791589
38.9599991	0.04300047	0.46089891	39.1199989	0.03079213	0.49821115	39.2700005	0.0468359	0.54000026
39.9799995	0.04707813	0.46270081	40.0699997	0.03345618	0.49949875	40.3199997	0.05094203	0.54211837
40.9900017	0.05131529	0.4645732	41.0699997	0.03614057	0.5007962	41.1699982	0.05525743	0.54434443
41.9700012	0.05554942	0.46644425	42.0699997	0.03881196	0.50208735	42.2200012	0.06018153	0.54688448
43	0.06113427	0.46891218	43.1199989	0.04247386	0.50385726	43.2700005	0.06482104	0.54927772
43.9700012	0.06629714	0.47119364	44.1199989	0.04630508	0.50570899	44.2700005	0.0693547	0.55161637
45.0200005	0.07193865	0.47368661	45.0699997	0.05060171	0.50778568	45.3199997	0.07461228	0.55432844
45.9700012	0.07777499	0.47626567	46.0400009	0.05579427	0.51029539	46.2200012	0.07889059	0.55653536
46.9599991	0.08436803	0.47917911	47.1199989	0.06165659	0.51312882	47.2200012	0.08344135	0.55888283
48.0200005	0.09015224	0.48173514	48.0699997	0.06787371	0.51613373	48.2200012	0.08758966	0.5610227
49.0099983	0.09661883	0.48459271	49.1699982	0.07492793	0.51954323	49.2700005	0.09176594	0.56317699
50.0200005	0.10288828	0.48736316	50.1699982	0.08184576	0.52288681	50.2200012	0.0946862	0.56468338
50.9700012	0.1084415	0.48981711	51.0699997	0.08913157	0.52640826	51.1699982	0.09791613	0.56634951
52.0200005	0.11333332	0.49197879	52.0200005	0.09596085	0.52970904	52.2200012	0.10072939	0.5678007
52.9700012	0.11885337	0.49441808	53.1699982	0.10241413	0.53282809	53.2200012	0.10353722	0.56924909
54.0699997	0.12369231	0.4965564	54.1699982	0.108521	0.53577971	54.3199997	0.10641554	0.57073385
55.0200005	0.12794499	0.49843565	55.1699982	0.11347863	0.53817588	55.2700005	0.10871773	0.57192141
56.0200005	0.13185816	0.50016487	56.1199989	0.11745796	0.5400992	56.2700005	0.11103471	0.5731166
57.0699997	0.13573175	0.50187659	57.1199989	0.12136132	0.54198581	57.2200012	0.11275003	0.57400143
58.0200005	0.13937117	0.50348485	58.1199989	0.12471948	0.5436089	58.2200012	0.11529822	0.57531589
59.0699997	0.14242993	0.5048365	59.1199989	0.12831701	0.54534769	59.2299995	0.11736851	0.57638383
59.9700012	0.14572931	0.50629449	60.1199989	0.13133887	0.54680824	60.2200012	0.11952591	0.57749671
60.9700012	0.14847838	0.50750929	61.1199989	0.13416848	0.54817587	61.1899986	0.12127578	0.57839936
61.9700012	0.15119641	0.50871038	62.0699997	0.13683024	0.54946238	62.1699982	0.12320799	0.57939607
62.9199982	0.15406093	0.50997621	63.1199989	0.13955293	0.55077833	63.2200012	0.12536227	0.58050734
63.9199982	0.15632307	0.51097584	64.1699982	0.1421941	0.55205488	64.2699966	0.12717234	0.58144104
64.9700012	0.15892173	0.51212418	65.1200028	0.14476559	0.55329776	65.2699966	0.12942277	0.5826019
65.9700012	0.16088361	0.51299113	66.0699997	0.14684269	0.55430168	66.1699982	0.13109025	0.58346206
66.9700012	0.16320496	0.51401693	67.1200028	0.14940851	0.55554181	67.2699966	0.13291603	0.58440387
67.9700012	0.16533895	0.51495993	68.1200028	0.15142148	0.55651474	68.2200012	0.13445295	0.58519667
68.9700012	0.16719374	0.51577955	69.1200028	0.15335763	0.55745053	69.2200012	0.13618005	0.58608758
70.0199966	0.1687739	0.51647782	70.0199966	0.15501099	0.55824965	70.1699982	0.13792171	0.58698601
71.0199966	0.17030887	0.51715612	71.1200028	0.15749394	0.55944973	71.2399979	0.13985011	0.58798075
71.9700012	0.17205615	0.51792824	72.0199966	0.15894432	0.56015074	72.1699982	0.14128048	0.58871859
72.9700012	0.17413997	0.51884907	73.1200028	0.16052012	0.56091237	73.2699966	0.14275095	0.58947712
74.0199966	0.17493956	0.51920241	74.0699997	0.16192253	0.56159019	74.2200012	0.14427955	0.59026563
74.9199982	0.17640264	0.51984894	75.0699997	0.16355111	0.56237733	75.1699982	0.14581033	0.59105527
75.9700012	0.17769253	0.52041894	76.0699997	0.16543694	0.56328881	76.2699966	0.14723274	0.59178901
77.0199966	0.17858249	0.52081221	77.1699982	0.16661268	0.56385708	77.3199997	0.14868946	0.59254044
77.9700012	0.17988223	0.52138656	78.0199966	0.16802471	0.56453955	78.1699982	0.15005767	0.59324622
78.9899979	0.18116551	0.52195364	79.1699982	0.16895443	0.56498891	79.2699966	0.15114695	0.59380811
79.9700012	0.18207368	0.52235496	80.1200028	0.17063234	0.56579989	80.2200012	0.15268566	0.59460247
81.0199966	0.18325971	0.52287906	81.0699997	0.17184631	0.56638664	81.2699966	0.15399903	0.59527934
82.0699997	0.18428685	0.52333295	82.1200028	0.17290243	0.56689709	82.3199997	0.15507537	0.59583455
82.9800034	0.18515564	0.52371687	83.1200028	0.1736796	0.56727272	83.1699982	0.15623675	0.59643364
83.9199982	0.18580982	0.52400595	84.1200028	0.17487631	0.56785113	84.2200012	0.15741269	0.59704024
85.0199966	0.18663396	0.52437013	85.0699997	0.17587596	0.56833428	85.2699966	0.15854633	0.59762502
86.0199966	0.18729786	0.52466351	86.0800018	0.17690026	0.56882936	86.2699966	0.15995533	0.59835184
87.0199966	0.18846959	0.52518129	87.0800018	0.17803395	0.5693773	87.3199997	0.16108725	0.59893572
87.9700012	0.18908614	0.52545375	88.0699997	0.1785794	0.56964093	88.2699966	0.16199823	0.59940565
89.0199966	0.18950941	0.52564079	89.0699997	0.17958706	0.57012796	89.2200012	0.16457346	0.60073406
89.9700012	0.19031925	0.52599865	90.1200028	0.18060989	0.57062232	90.2200012	0.16513977	0.60102618

Replicate #2 (Tm plots graphed with temperature vs normalized absorbance)								
N20-1			N15-1			Dz 10-23		
Temperature	Normalized A	Abs	Temperature	Normalized A	Abs	Temperature	Normalized A	Abs
19.9200001	0	0.49162689	20.0200005	0	0.46010154	19.9699993	0	0.42755529
20.9200001	0.00108643	0.49216101	20.9699993	0.00010131	0.46014816	21.0699997	0.001172	0.42805639
21.9899998	0.00204525	0.49263239	22.1299992	0.00269619	0.46134207	22.2199993	0.00199883	0.4284099
22.9699993	0.00413069	0.49365765	23.0200005	0.00374183	0.46182317	23.2199993	0.00361311	0.4291001
24	0.00568123	0.49441993	24.0699997	0.00388232	0.46188781	24.2199993	0.00461859	0.42952999
24.9699993	0.00638884	0.49476781	25.1200008	0.00456659	0.46220264	25.1700001	0.00634042	0.43026617
25.9200001	0.00817034	0.49564365	26.0200005	0.00597172	0.46284914	26.1700001	0.00684291	0.43048102
27.0200005	0.01083633	0.49695432	27.1200008	0.00749719	0.46355101	27.2700005	0.00872213	0.43128449
28.0200005	0.01280381	0.49792159	28.2199993	0.0086249	0.46406987	28.3199997	0.01086637	0.43220127
28.9699993	0.01380731	0.49841493	29.1200008	0.00981368	0.46461684	29.2199993	0.01129024	0.43238249
30.0699997	0.01646852	0.49972326	30.0699997	0.01140277	0.46534798	30.3199997	0.01360581	0.43337253
30.9200001	0.01805021	0.50050086	31.0699997	0.01245061	0.46583009	31.1200008	0.01610944	0.43444297
32.0200005	0.02015542	0.50153583	32.1199989	0.01431731	0.46668896	32.1199989	0.01729343	0.43494919
32.9700012	0.02256105	0.50271851	33.1199989	0.01581234	0.46737683	33.2200012	0.01955958	0.43591809
34.0200005	0.02576056	0.50429147	34.0699997	0.01755002	0.46817634	34.2200012	0.02188825	0.43691373
34.9700012	0.02801526	0.50539994	35.2200012	0.01939437	0.46902493	35.3199997	0.02485297	0.43818131
36.0200005	0.03114227	0.50693727	36.0699997	0.02115731	0.46983606	36.2200012	0.0275142	0.43931913
37.0200005	0.03453965	0.50860751	37.0600014	0.02295691	0.47066405	37.1699982	0.03000333	0.44038337
38.0200005	0.03883929	0.51072133	38.0699997	0.02509184	0.47164634	38.2700005	0.03392237	0.44205898
38.9700012	0.04271422	0.51262635	39.0699997	0.02732775	0.47267509	39.2200012	0.03776194	0.44370061
39.9700012	0.04750937	0.51498377	40.0699997	0.02978778	0.47380695	40.1399994	0.04210353	0.44555688
41	0.05247535	0.51742518	41.1699982	0.03315159	0.47535464	41.3199997	0.04757369	0.44789568
42.0699997	0.05817288	0.52022624	42.1699982	0.03610622	0.47671407	42.2200012	0.05261301	0.45005026
42.9799995	0.06351591	0.52285302	43.2200012	0.0400004	0.47850579	43.2700005	0.05802671	0.45236492
43.9199982	0.06875637	0.52542937	44.1199989	0.04401816	0.48035437	44.2700005	0.06395874	0.45490119
44.9700012	0.07448082	0.52824366	45.0699997	0.04876132	0.4825367	45.1699982	0.06983437	0.45741335
46.0200005	0.07994981	0.53093237	46.0600014	0.05457842	0.48521316	46.2700005	0.07692417	0.46044463
46.9700012	0.0854587	0.53364068	47.1199989	0.06111975	0.48822284	47.2700005	0.084394	0.4636384
48.0699997	0.09012788	0.53593618	48.0699997	0.06877	0.49174273	48.1699982	0.09125072	0.46657002
48.8699989	0.09448923	0.53808033	49.1199989	0.07617885	0.49515155	49.1699982	0.09890938	0.46984452
49.9700012	0.09893715	0.54026705	50.0200005	0.08534466	0.49936876	50.2200012	0.10492748	0.47241759
50.9700012	0.10179573	0.54167241	51.0200005	0.09313645	0.50295377	51.1699982	0.11214094	0.47550175
51.9700012	0.10552652	0.54350656	52.0200005	0.10168588	0.50688738	52.1699982	0.11829943	0.47813484
53	0.1090872	0.54525709	53.1199989	0.11026395	0.51083416	53.2700005	0.12435134	0.48072237
54.0200005	0.11147696	0.54643196	54.1699982	0.11659088	0.51374519	54.2200012	0.12891403	0.48267317
55.0200005	0.11453474	0.54793525	55.1199989	0.12275316	0.51658046	55.1699982	0.13365258	0.48469916
55.9199982	0.11725548	0.54927284	56.2200012	0.12818233	0.51907843	56.1699982	0.1383539	0.48670924
56.9700012	0.11941015	0.55033213	57.1699982	0.13268538	0.52115029	57.1699982	0.14258271	0.48851728
57.9700012	0.12180524	0.55150962	58.1199989	0.13654069	0.52292413	58.2200012	0.14605913	0.49000365
59.0200005	0.12418772	0.55268091	59.1699982	0.14054212	0.52476519	59.2200012	0.1495927	0.49151444
60.0200005	0.12637149	0.55375451	60.1199989	0.14368791	0.52621257	60.2200012	0.15277351	0.49287441
61.0699997	0.12873129	0.55491465	61.1699982	0.14708462	0.52777541	61.2200012	0.15595905	0.49423641
62.0200005	0.13111426	0.55608618	62.0699997	0.14987726	0.5290603	62.2200012	0.15894489	0.49551302
62.9799995	0.13357372	0.55729532	63.0699997	0.15275644	0.53038502	63.2700005	0.16164146	0.49666595
63.9700012	0.13555623	0.55826998	64.0699997	0.15585791	0.53181201	64.2699966	0.16448929	0.49788356
64.9800034	0.13740308	0.55917794	65.1200028	0.15832073	0.53294516	65.2200012	0.16696371	0.49894151
65.9199982	0.13936388	0.56014192	66.1200028	0.16052432	0.53395903	66.1500015	0.16907965	0.49984619
66.8700028	0.14159142	0.56123704	67.0699997	0.16309711	0.53514278	67.2200012	0.17125323	0.50077552
67.9700012	0.14328986	0.56207204	68.1699982	0.16556485	0.53627819	68.1699982	0.17333347	0.50166494
68.8700028	0.14478002	0.56280464	69.1200028	0.16750857	0.5371725	69.1699982	0.17573924	0.50269353
69.8700028	0.14674956	0.56377292	70.0699997	0.16976735	0.53821176	70.1699982	0.17744699	0.50342369
70.9700012	0.14871291	0.56473815	71.2200012	0.17199192	0.53923529	71.2200012	0.17906942	0.50411737
71.9700012	0.15017409	0.56545651	72.0699997	0.17387592	0.54010212	72.1699982	0.18069213	0.50481117
72.9700012	0.15185023	0.56628054	73.0699997	0.175538	0.54086685	73.1699982	0.18196478	0.5053553
74.0199966	0.15364045	0.56716067	74.1200028	0.17717522	0.54162014	74.1699982	0.18346398	0.50599629
75.0199966	0.15521438	0.56793445	75.1200028	0.1789719	0.54244679	75.2699966	0.18524101	0.50675607
76.0199966	0.1571324	0.5688774	76.1200028	0.18019016	0.54300731	76.2699966	0.18642542	0.50726247
76.9199982	0.15802157	0.56931454	77.1200028	0.18180988	0.54375255	77.2200012	0.187632	0.50777835
78.0199966	0.15977542	0.57017678	78.1200028	0.18350098	0.54453063	78.2200012	0.18871743	0.50824243
78.9899979	0.16145483	0.57100242	79.0699997	0.18442789	0.5449571	79.2699966	0.18980356	0.50870681
79.9199982	0.16255229	0.57154197	80.0199966	0.18597118	0.54566717	80.2699966	0.19110354	0.50926262
80.9599991	0.16403675	0.57227176	81.0199966	0.18700833	0.54614437	81.2699966	0.19194905	0.50962412
81.9199982	0.16565117	0.57306546	82.0699997	0.1882297	0.54670632	82.1699982	0.19288238	0.51002318
82.9199982	0.16642068	0.57344377	83.0199966	0.18945288	0.54726911	83.1200028	0.19381474	0.51042181
83.9700012	0.16817369	0.57430559	84.1200028	0.19056827	0.5477823	84.1900024	0.19488833	0.51088083
84.9300003	0.16931613	0.57486725	85.1200028	0.19171139	0.54830825	85.2200012	0.19528397	0.51104999
86.0199966	0.17162404	0.57600188	86.0699997	0.19250279	0.54867238	86.1699982	0.1963397	0.51150137
87	0.17236203	0.5763647	87.1200028	0.19365615	0.54920304	87.2200012	0.19745385	0.51197773
87.9700012	0.17329982	0.57682574	88.1200028	0.19464783	0.54965931	88.3199997	0.19788379	0.51216155
88.949997	0.17430332	0.57731909	89.1699982	0.19537264	0.5499928	89.1699982	0.19871996	0.51251906
90.0199966	0.17554978	0.57793188	90.0299988	0.196291	0.55041534	90.2200012	0.19907851	0.51267236

SI Figure 12a. Temperature optimum of RNA cleavage of Dz 10-23.

Rep #1	Temp (˚C)	Product (sign	Total (signal)	% Cleavage
Dz10-23	16	367	2927	12.5384353
	20	6710	14990	44.7631755
	30	25200	27950	90.1610018
	37	12100	14230	85.0316233
	44	12800	25200	50.7936508
	50	1750	19350	9.04392765

Rep #2	Temp (˚C)	Product (sign	Total (signal)	% Cleavage
Dz10-23	16	1370	8650	15.8381503
	20	2360	6760	34.9112426
	30	24600	27650	88.9692586
	37	16800	19260	87.2274143
	44	12200	24700	49.3927126
	50	416	5086	8.17931577

Rep #3	Temp (˚C)	Product (sign	Total (signal)	% Cleavage
Dz10-23	16	832	3642	22.8445909
	20	2150	4610	46.637744
	30	6100	7570	80.5812417
	37	4020	4777	84.1532342
	44	5490	12410	44.2385173
	50	2090	16590	12.5979506

Average	Temp (˚C)	% Cleavage	STDEV
Dz10-23	16	17.0737255	5.2630025
	20	42.104054	6.29927806
	30	86.5705007	5.22096545
	37	85.4707573	1.58343765
	44	48.1416269	3.45200742
	50	9.940398	2.34175739

SI Figure 12b. Temperature optimum of RNA cleavage of Tz 6-29 and Tz 13-38.

Rep #1	Temp (˚C)	Product (sign	Total (signal)	% Cleavage
N15-1	40	467	20767	2.24876005
	45	1930	24130	7.99834231
	50	8300	26100	31.8007663
	55	10700	24900	42.9718876
	60	6600	28000	23.5714286
	65	816	12516	6.51965484
N20-1	40	683	12183	5.60617254
	45	2810	13910	20.201294
	50	8050	20150	39.9503722
	55	12600	27800	45.323741
	60	3720	18320	20.3056769
	65	555	14555	3.81312264

Rep #2	Temp (˚C)	Product (sign	Total (signal)	% Cleavage
N15-1	40	713	43613	1.63483365
	45	3190	45790	6.96658659
	50	10800	32400	33.3333333
	55	14400	31600	45.5696203
	60	6820	29420	23.1815092
	65	1500	19300	7.77202073
N20-1	40	1370	22770	6.01668863
	45	3390	17990	18.8438021
	50	10900	26100	41.7624521
	55	11500	26100	44.0613027
	60	5740	25340	22.6519337
	65	1230	21230	5.79368818

Rep #3	Temp (˚C)	Product (sign	Total (signal)	% Cleavage
N15-1	40	445	25345	1.75577037
	45	1250	16150	7.73993808
	50	3000	10950	27.3972603
	55	6790	17490	38.8221841
	60	3310	19610	16.8791433
	65	384	9034	4.25060881
N20-1	40	1170	20870	5.60613321
	45	3220	16120	19.9751861
	50	5500	13690	40.1753104
	55	6270	13850	45.2707581
	60	3040	16640	18.2692308
	65	9.13	9929.13	0.09195166

Average	Temp (˚C)	% Cleavage	STDEV
N15-1	40	1.87978802	0.32521023
	45	7.56828899	0.53686826
	50	30.8437866	3.08157401
	55	42.454564	3.40333534
	60	21.2106937	3.7562955
	65	6.18076146	1.78499916
N20-1	40	5.74299812	0.23702293
	45	19.6734274	0.72731686
	50	40.6293783	0.98769508
	55	44.8852673	0.71406584
	60	20.4089471	2.19317574
	65	3.23292083	2.89481009

SI Figure 12c. Temperature optimum of RNA cleavage of Tz N15-1and Tz N20-1.

Rep #1	Temp (˚C)	Product (sign	Total (signal)	% Cleavage
6-29	30	65.6	371.6	17.6533907
	37	67.7	235.7	28.7229529
	44	205	398	51.5075377
	51	143	434	32.9493088
	60	7.91	188.91	4.18717908
13-38	30	55.5	278.5	19.9281867
	37	370	499	74.1482966
	44	139	408	34.0686275
	51	106	637	16.6405024
	60	1.48	122.48	1.20836055

Rep #2	Temp (˚C)	Product (sign	Total (signal)	% Cleavage
6-29	30	57.9	471.9	12.2695486
	37	36.3	174.3	20.8261618
	44	91.4	238.4	38.3389262
	51	125	422	29.6208531
	60	3.49	87.09	4.00734872
13-38	30	166	386	43.0051813
	37	146	274	53.2846715
	44	169	411	41.1192214
	51	101	559	18.0679785
	60	1.7	122.7	1.38549307

Rep #3	Temp (˚C)	Product (sign	Total (signal)	% Cleavage
6-29	30	22.3	107.6	20.7249071
	37	125	278	44.9640288
	44	46	88.1	52.2133939
	51	20.6	51.6	39.9224806
	60	10.4	231.4	4.49438202
13-38	30	11.4	58.1	19.6213425
	37	96.8	172.8	56.0185185
	44	127	212.5	59.7647059
	51	12.7	64	19.84375
	60	4.91	152.91	3.21103917

Average	Temp (˚C)	% Cleavage	STDEV
6-29	30	16.8826155	4.28005168
	37	31.5043812	12.306966
	44	47.3532859	7.81463814
	51	34.1642142	5.25717403
	60	4.22963661	0.24627696
13-38	30	27.5182369	13.4129648
	37	61.1504955	11.3391183
	44	44.9841849	13.276881
	51	18.184077	1.60477662
	60	1.93496426	1.10865656

SI Table 4. Apparent activation energies.

Enzyme	T1	T2	k1	k1 SE	k2	k2 SE		Tcomb	Tcomb/R	ln (kcomb)	Ea (kJ/mol)	Ea SE
N15-1	310	323	0.0173068	0.0019389	0.0580815	0.0008165		0.0001298	1.5616E-05	1.2107477	77.532634	7.23061
N20-1	310	323	0.0213425	0.0008244	0.0736913	0.0013827		0.0001298	1.5616E-05	1.2391844	79.353631	2.748941
6-29	310	323	0.0715607	0.0040896	0.082439	0.0049342		0.0001298	1.5616E-05	0.1415126	9.0620395	5.299479
13-38	310	323	0.271877	0.0175549	0.130434	0.0079469		0.0001298	1.5616E-05	-0.7344824	-47.03404	5.685021
10-23	310	323	21.71568	1.081224	1.837182	0.402639		0.0001298	1.5616E-05	-2.4698017	-158.15866	14.392044

Figure 3a. and SI Figure 2a-b. Activity screen of chemically synthesized hits from round 8.

Replicate #1 (37˚C, top; 50˚C, bottom)

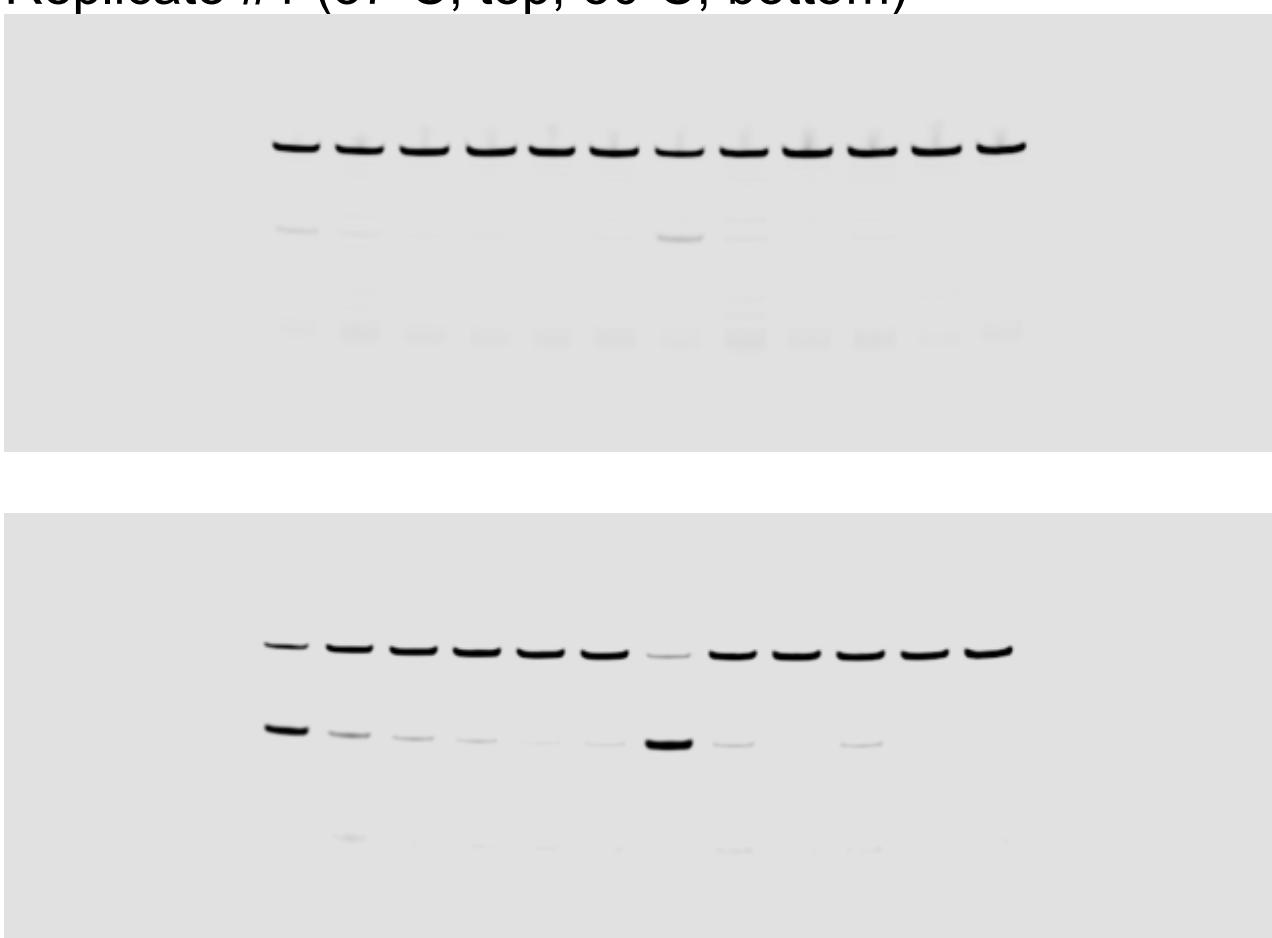

Replicate #2 (37˚C, top; 50˚C, bottom)

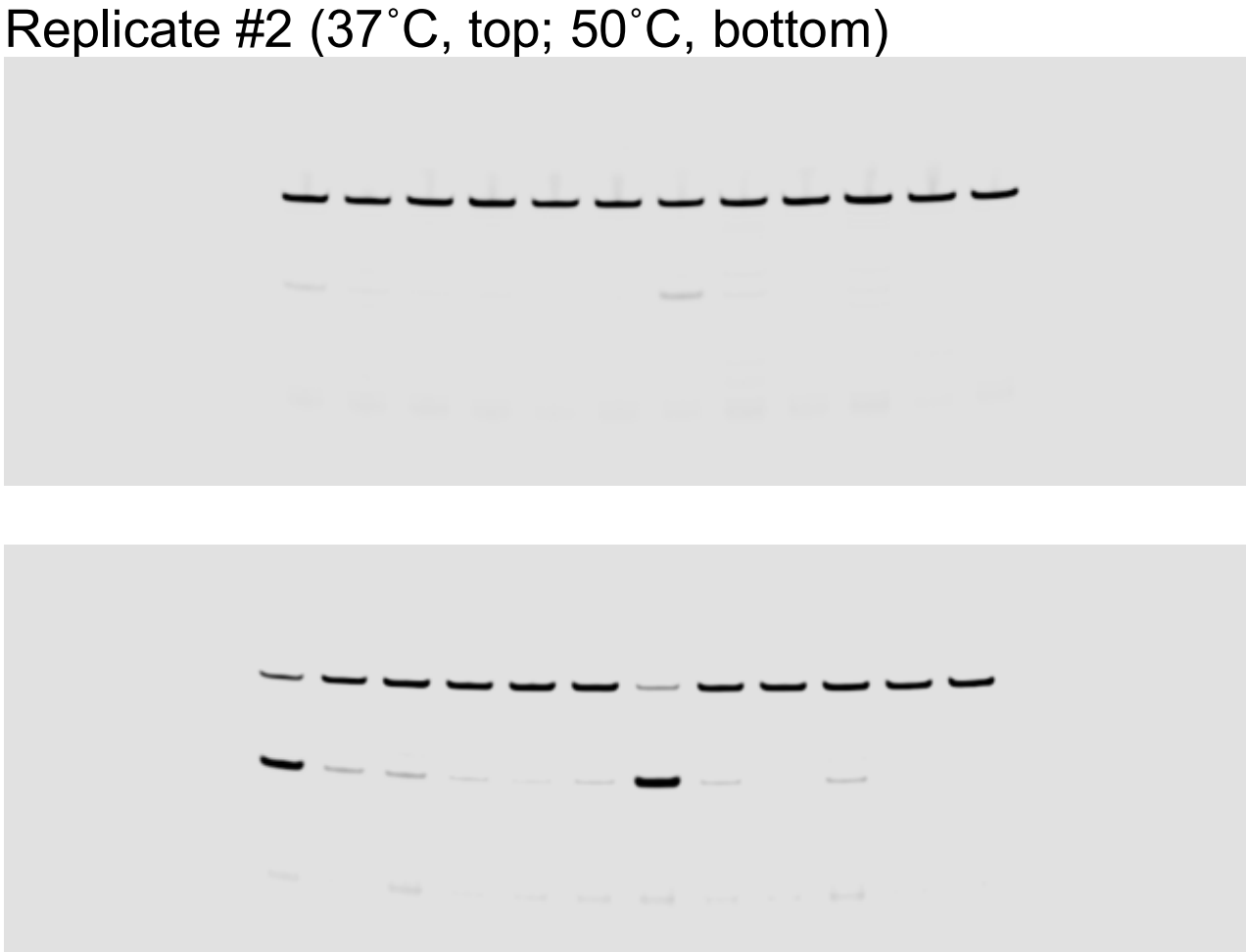

Replicate #3 (37˚C, top; 50˚C, bottom)

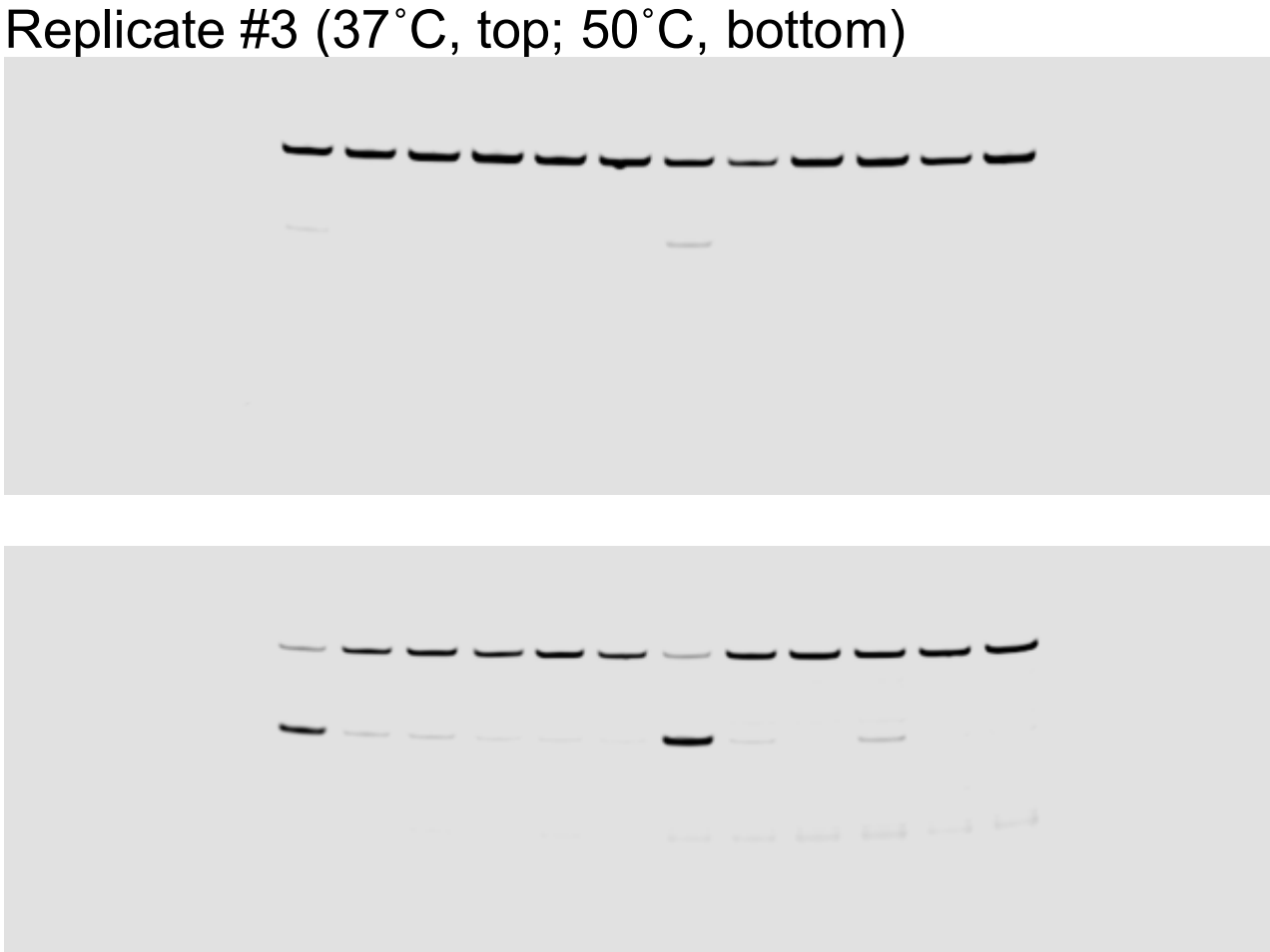

Figure 3b-c. and SI Figure 3a-b. Kinetic assays of Tz N15-1 and Tz N20-1.

Replicate #1 (37˚C, top; 50˚C, bottom)

N15-1 N20-1

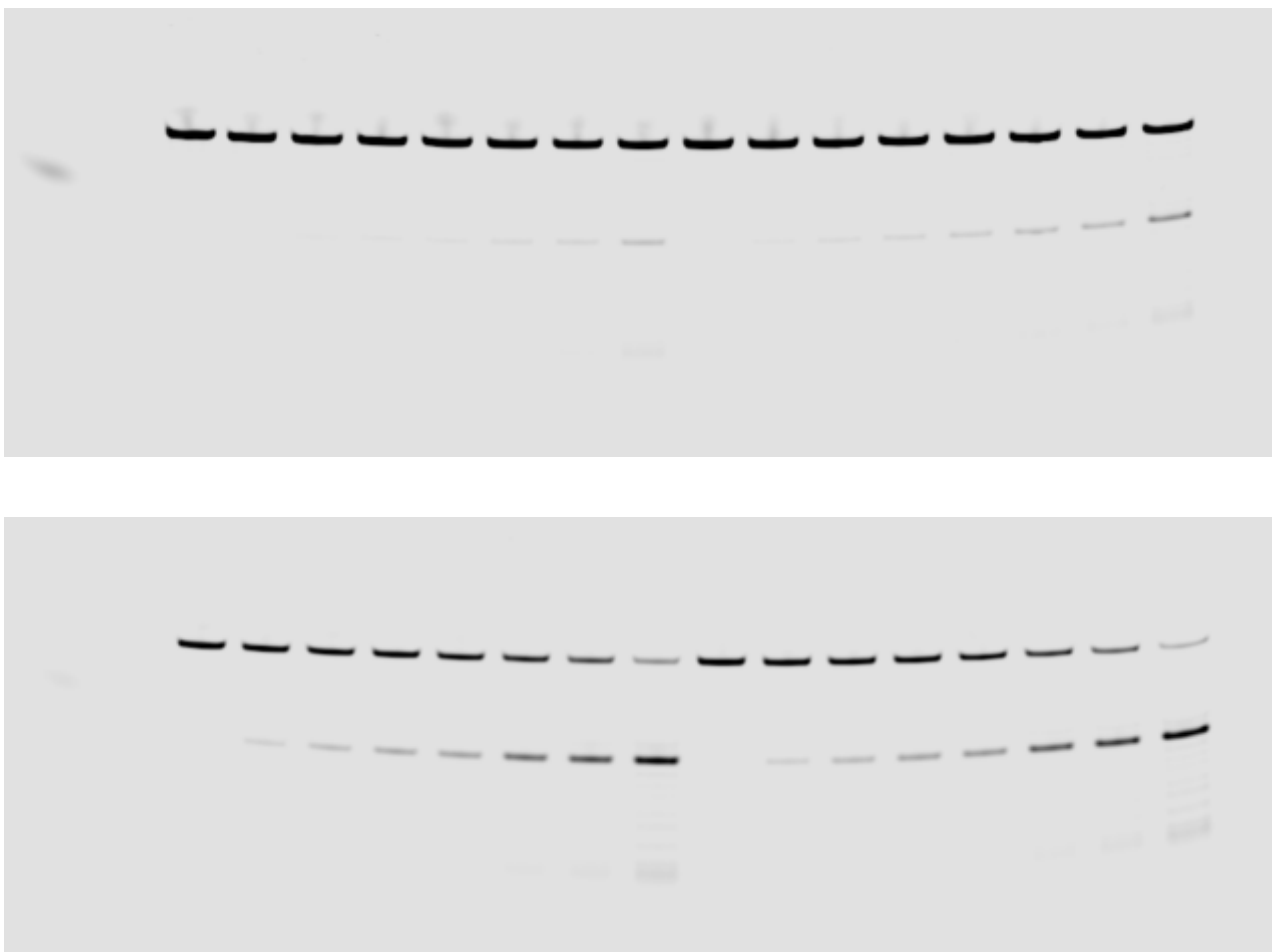

Replicate #2 (37˚C, top; 50˚C, bottom)

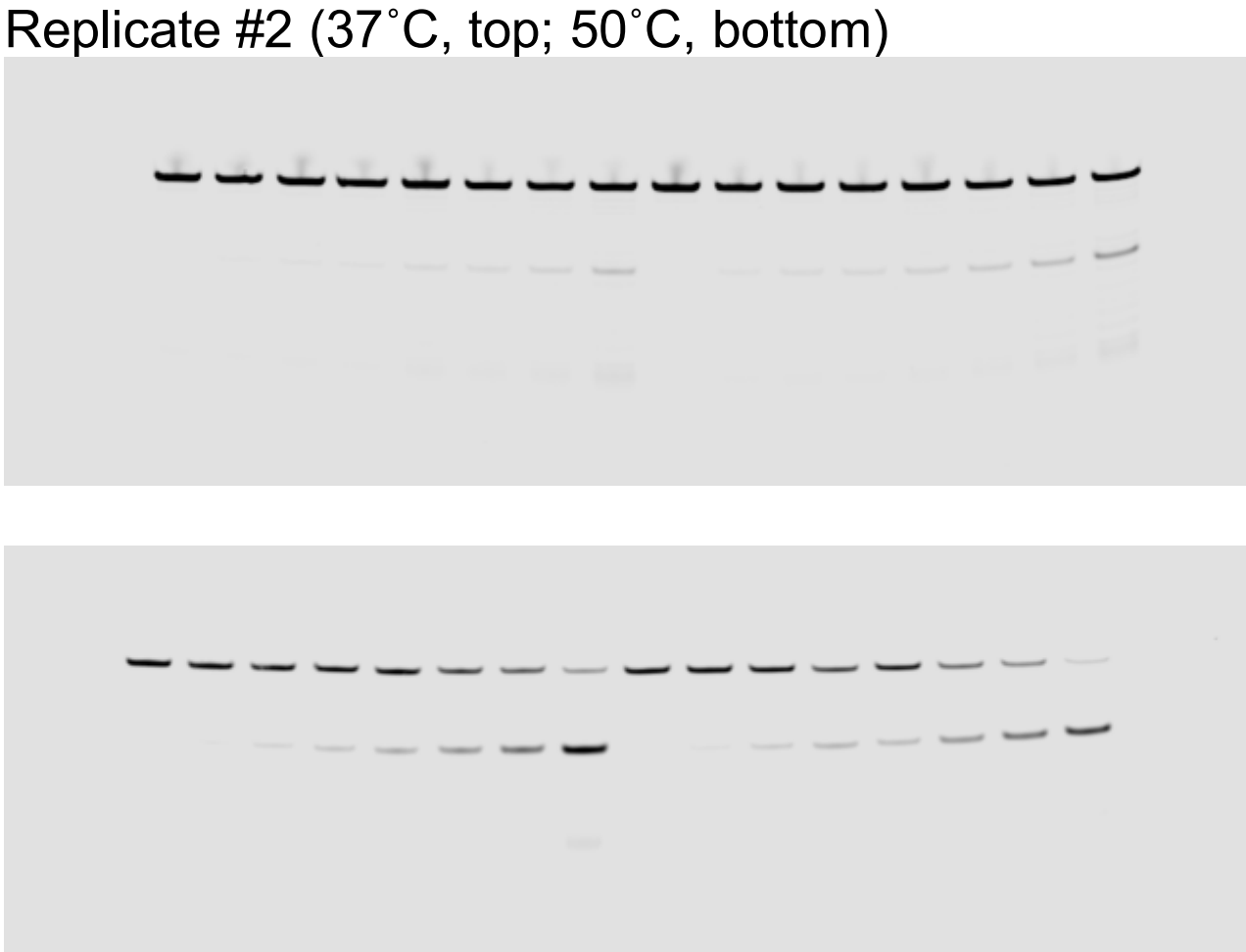

Replicate #3 (37˚C, top; 50˚C, bottom)

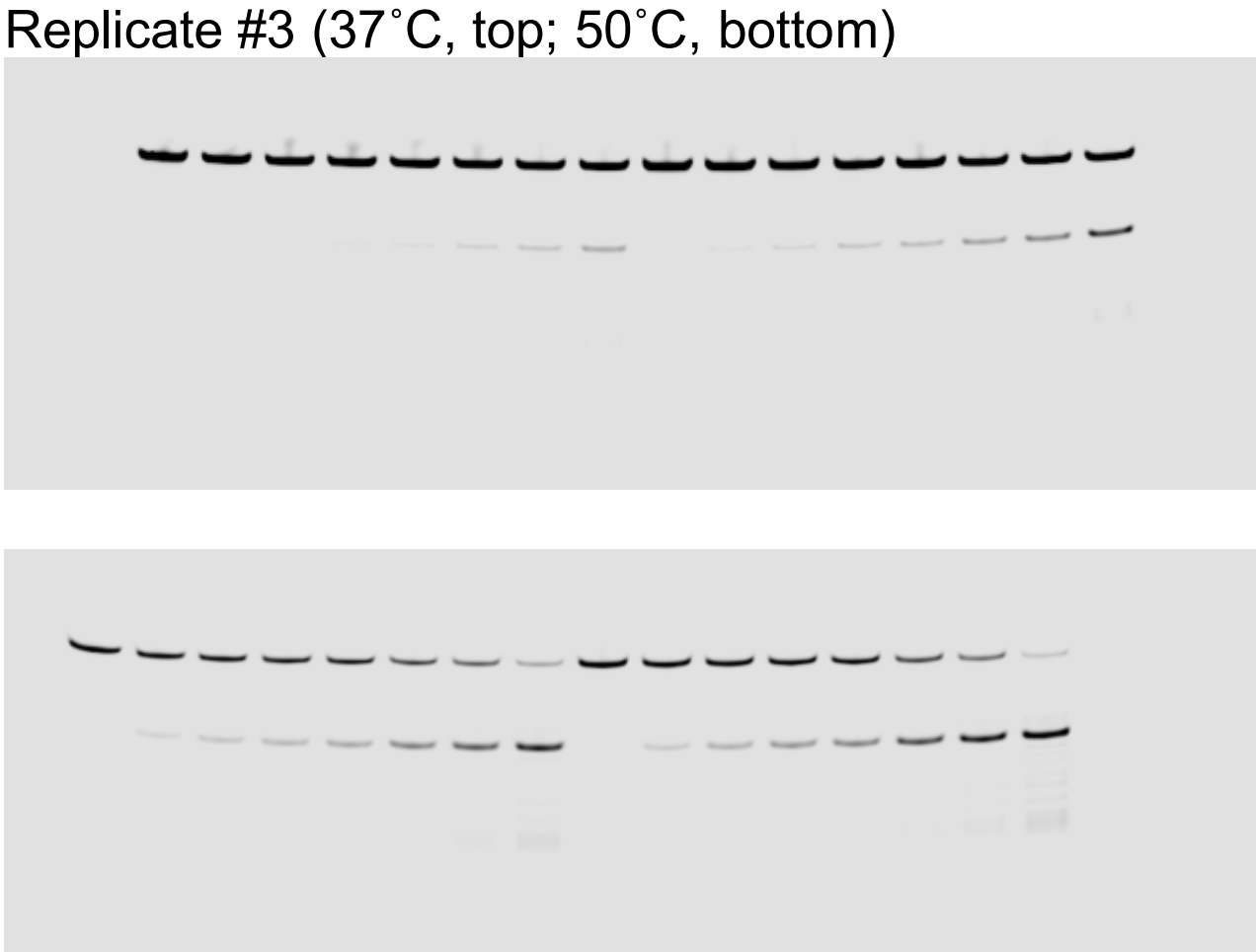

Figure 4a. and SI Figure 8a-b. Kinetic assays of Dz 10-23 (multiple turnover).

Replicate #1

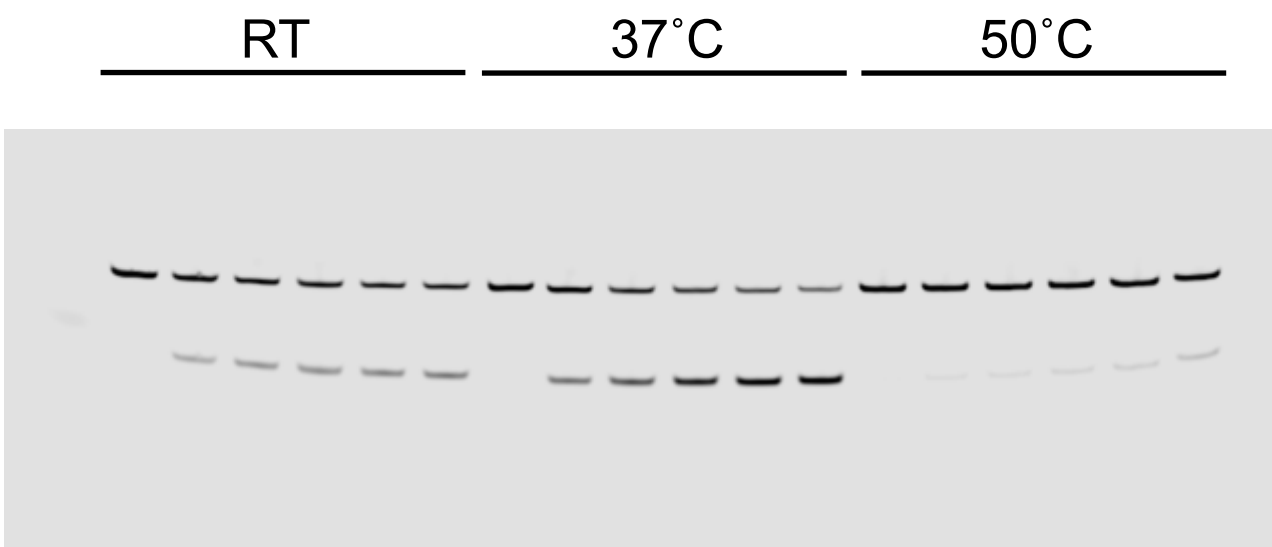

Replicate #2

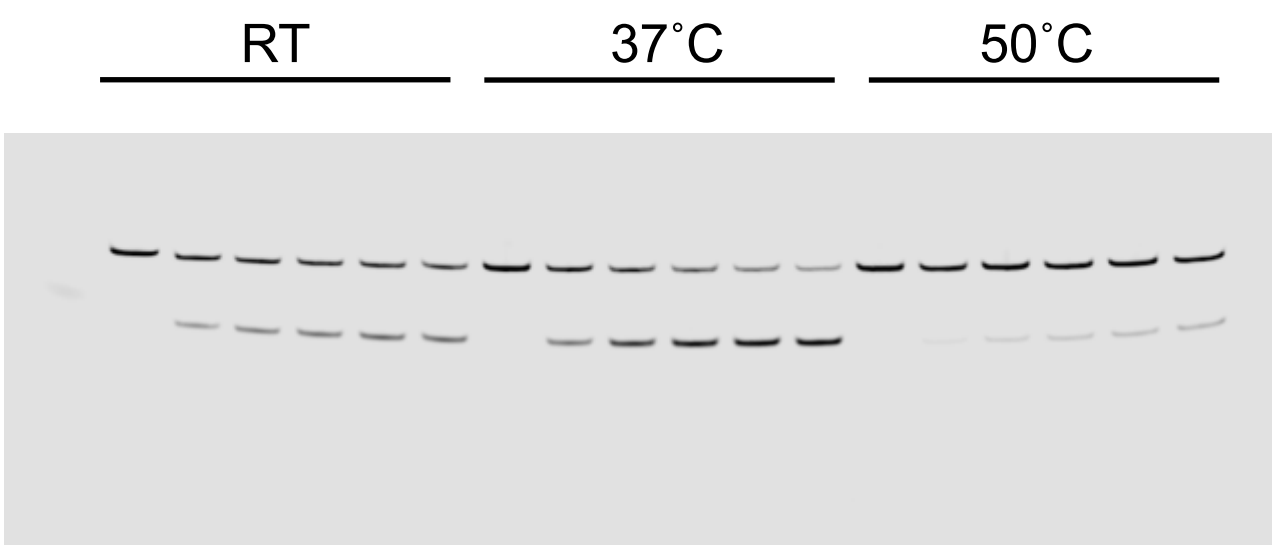

Replicate #3

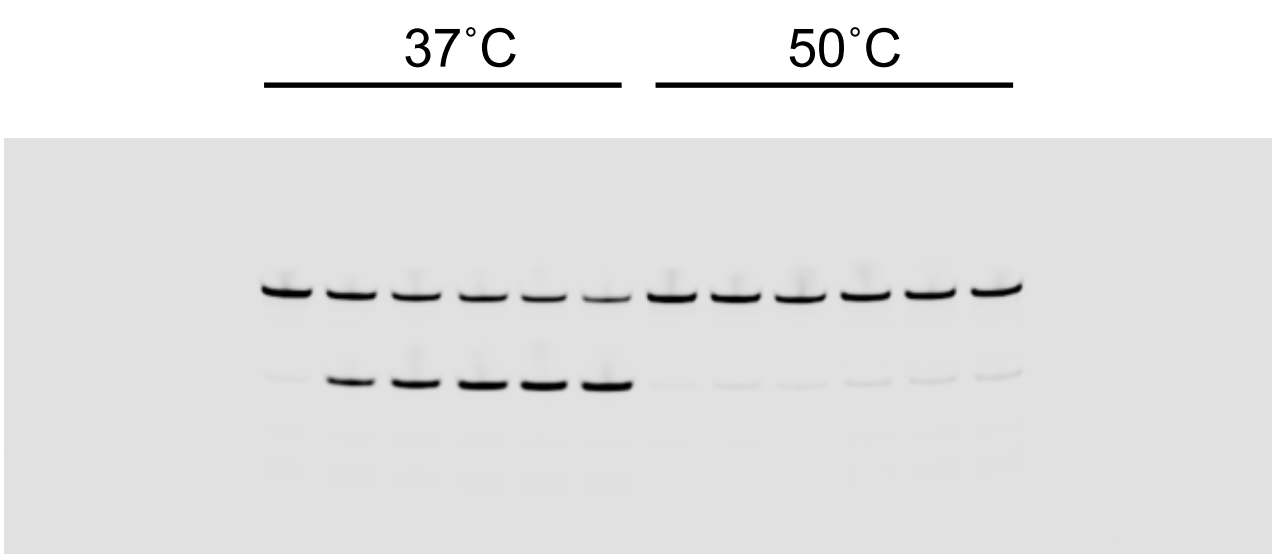

Figure 4b-c. and SI Figure 11a-b. Kinetic assays of Tz 6-29 and Tz 13-38.

Replicate #1 (37˚C, top; 50˚C, bottom)

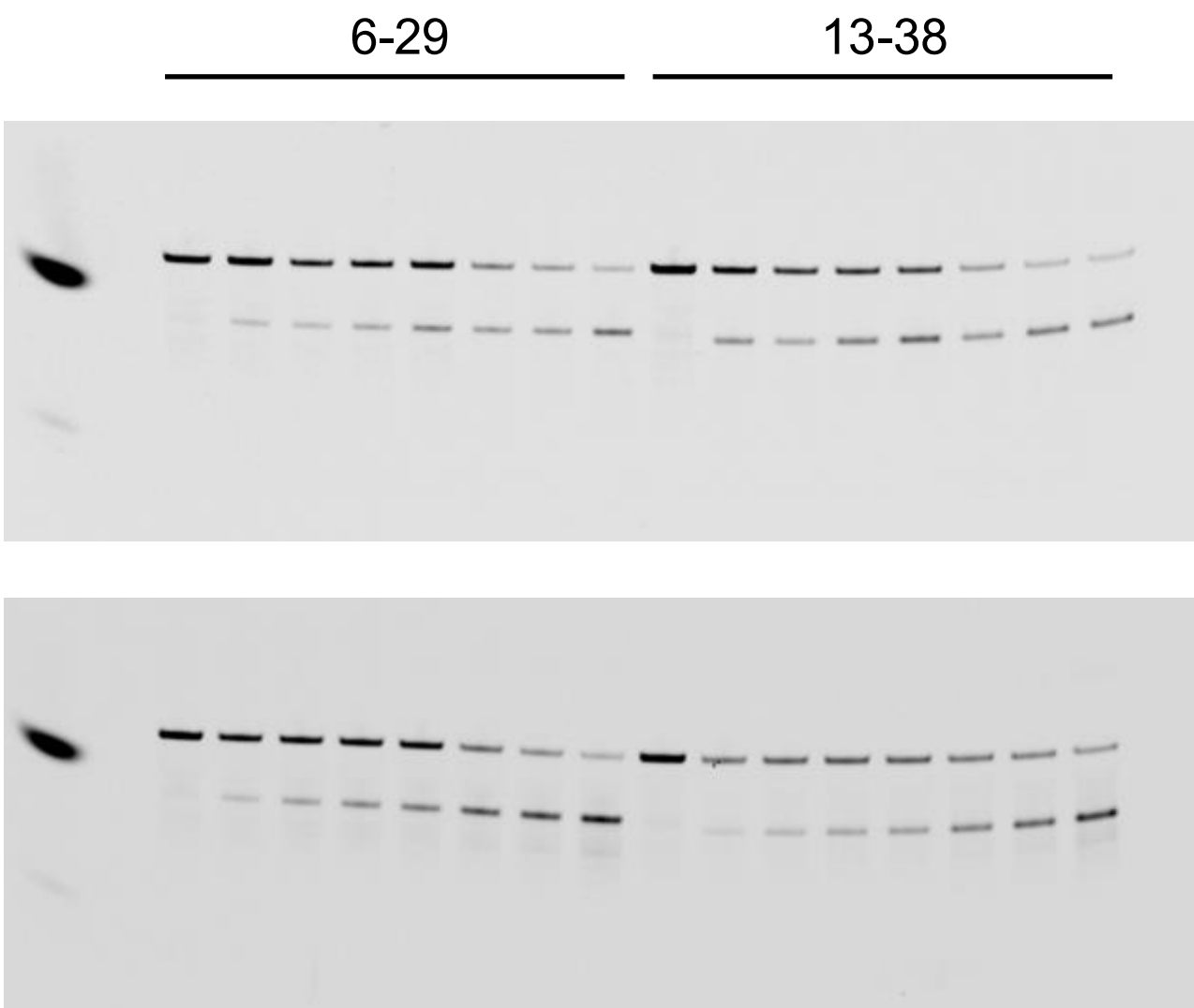

Replicate #2 (37˚C, top; 50˚C, bottom)

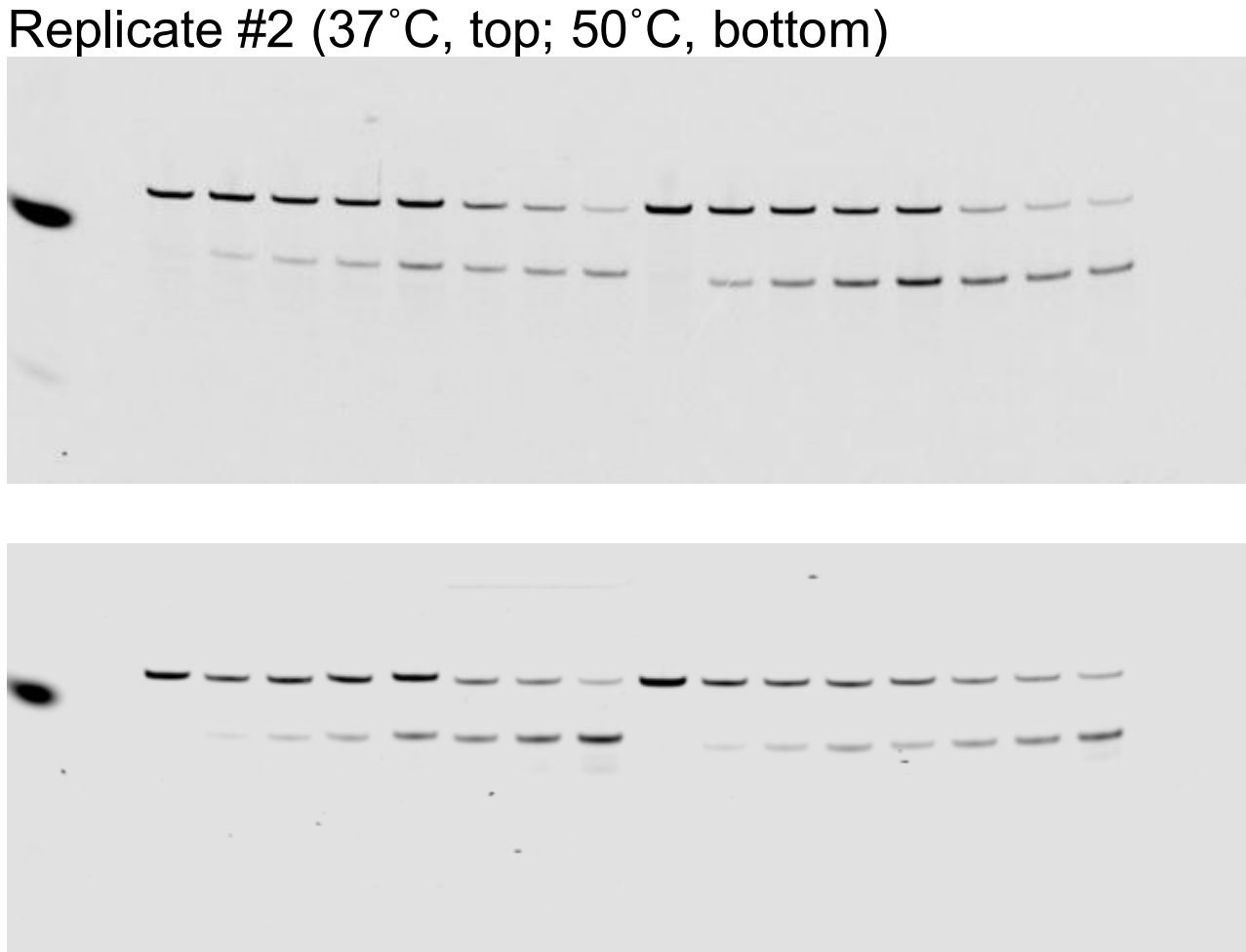

Replicate #3 (37˚C, top; 50˚C, bottom)

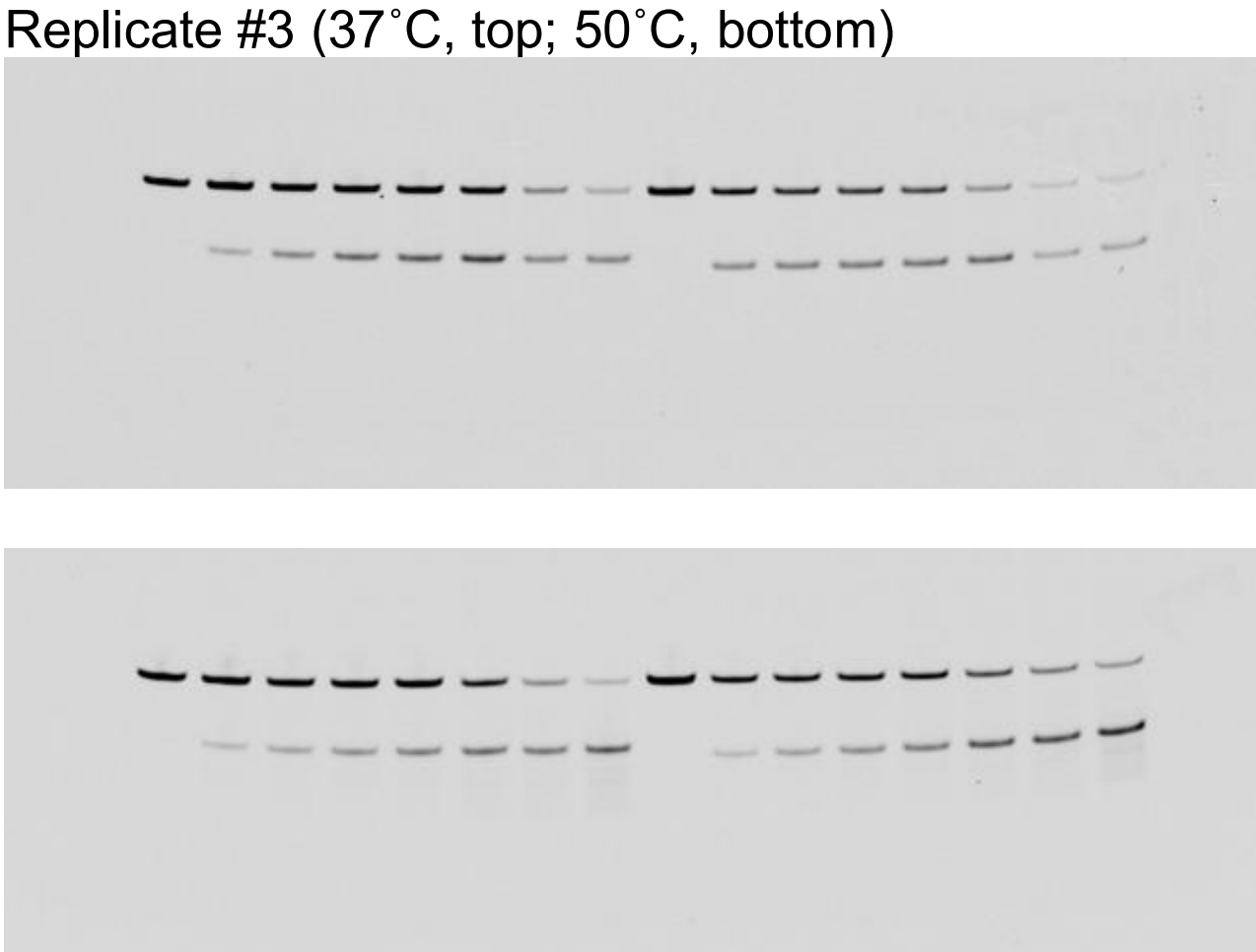

SI Figure 4. Product validation by RNase T1 digestion.

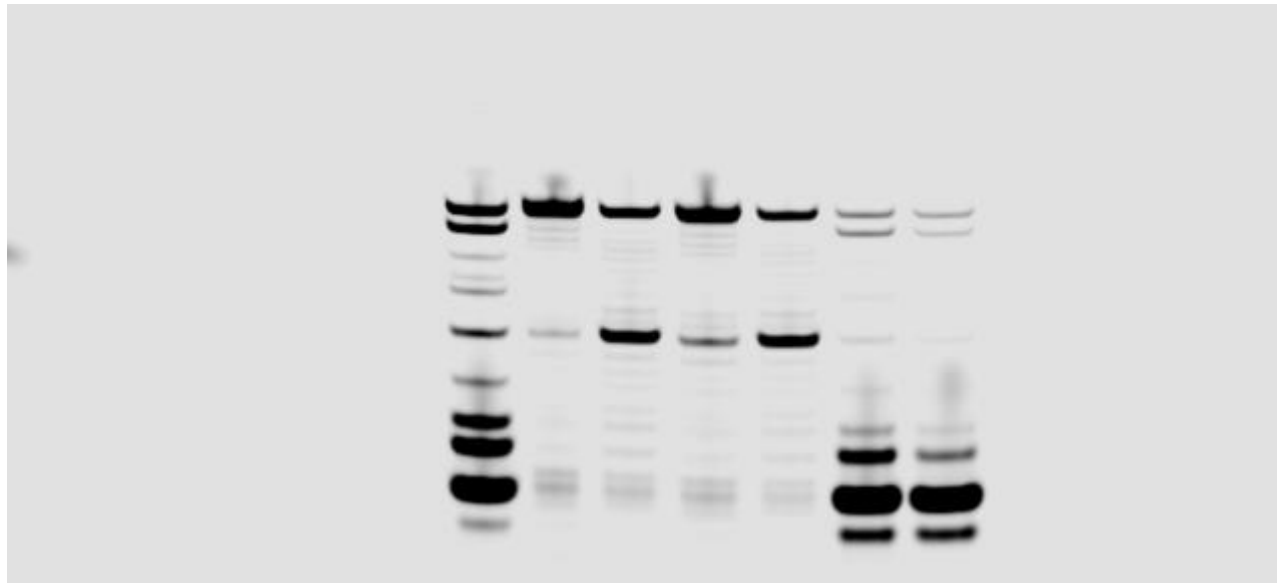

Note: The last 2 lanes are additional RNase T1 digestion reactions with longer incubation times (>1 min), which digested the entire RNA substrate.

SI Figure 5a-b. Magnesium dependence.

Replicate #1

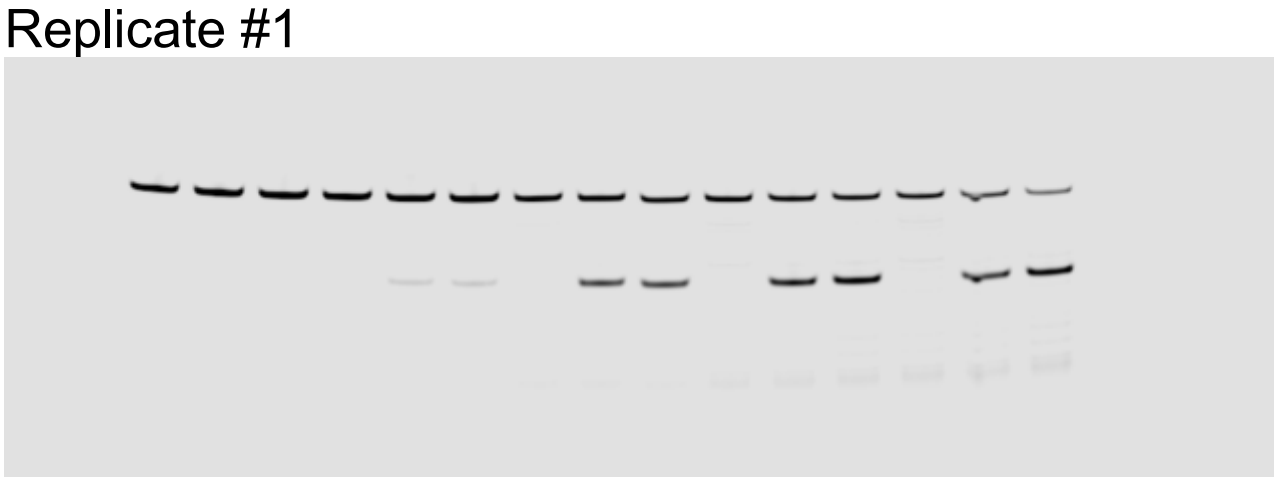

Replicate #2

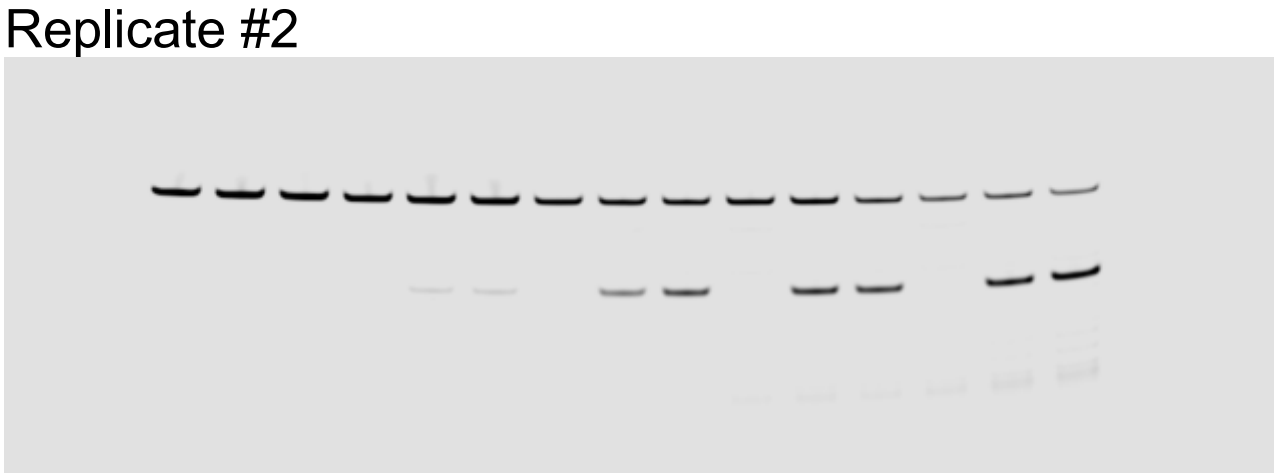

Replicate #3

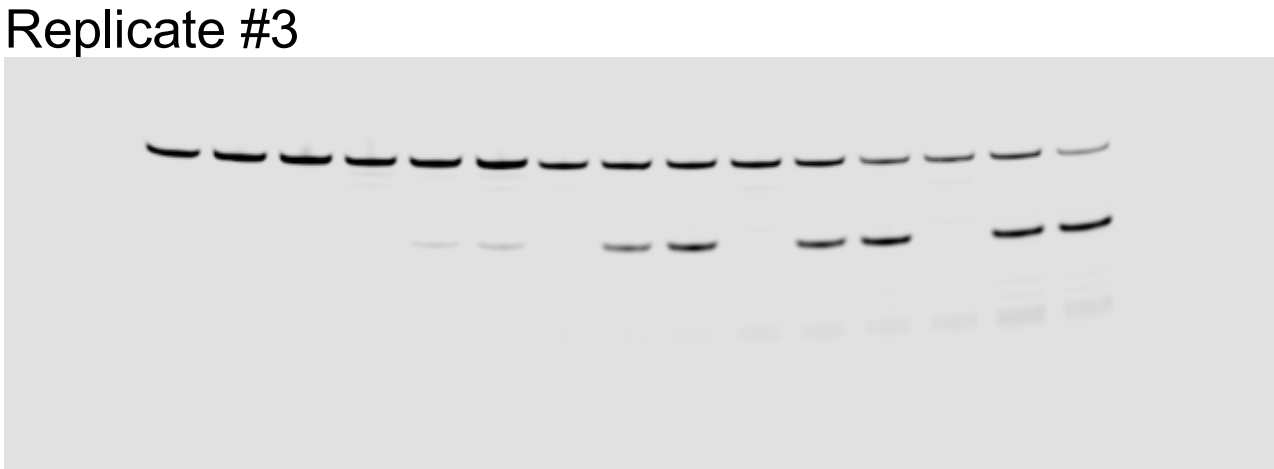

SI Figure 6a. Activity of Dz 10-23 pre-treated with TURBO DNase.

Replicate #1 (lanes 1-4), Replicate #2 (lanes 5-8)

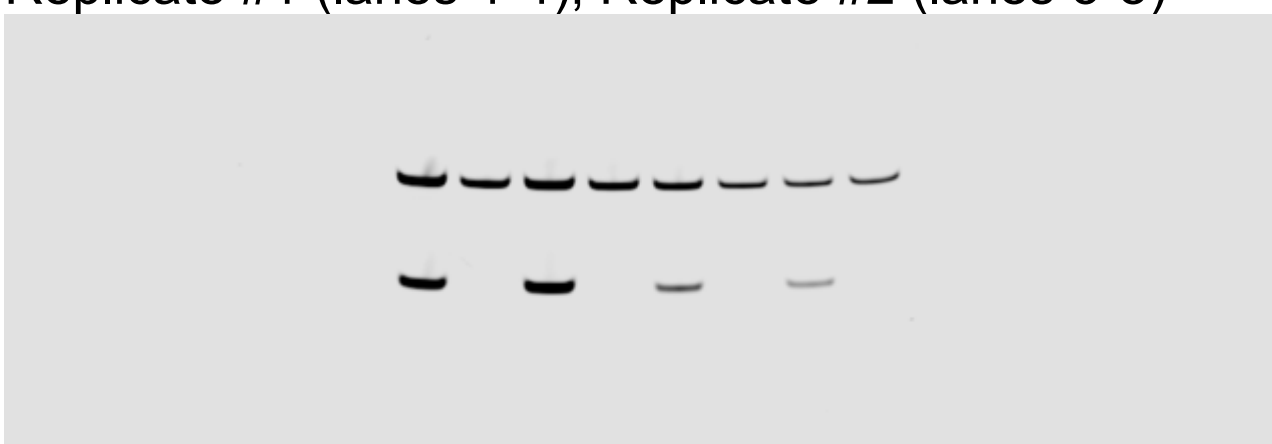

SI Figure 6b-c. Activity of Tz N15-1 and Tz N20-1 pre-treated with TURBO DNase.

Replicate #1 (lanes 1-8), Replicate #2 (lanes 9-16)

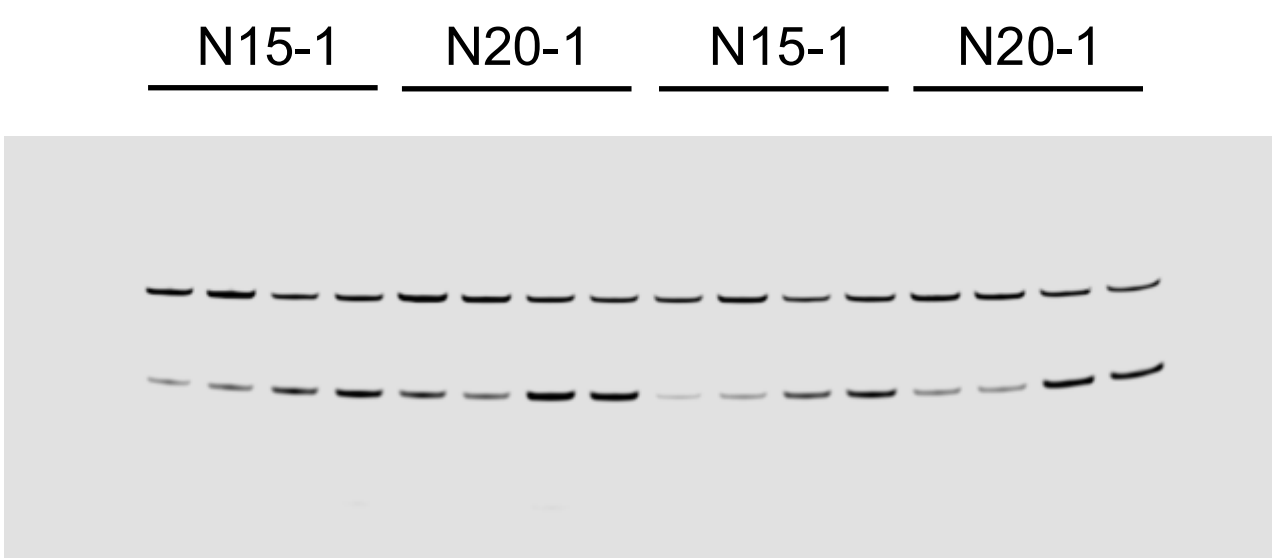

SI Figure 7a. Activity of Dz 10-23 in absence and presence of RNase H1.

Replicate #1 (lanes 1-6), Replicate #2 (lanes 7-12)

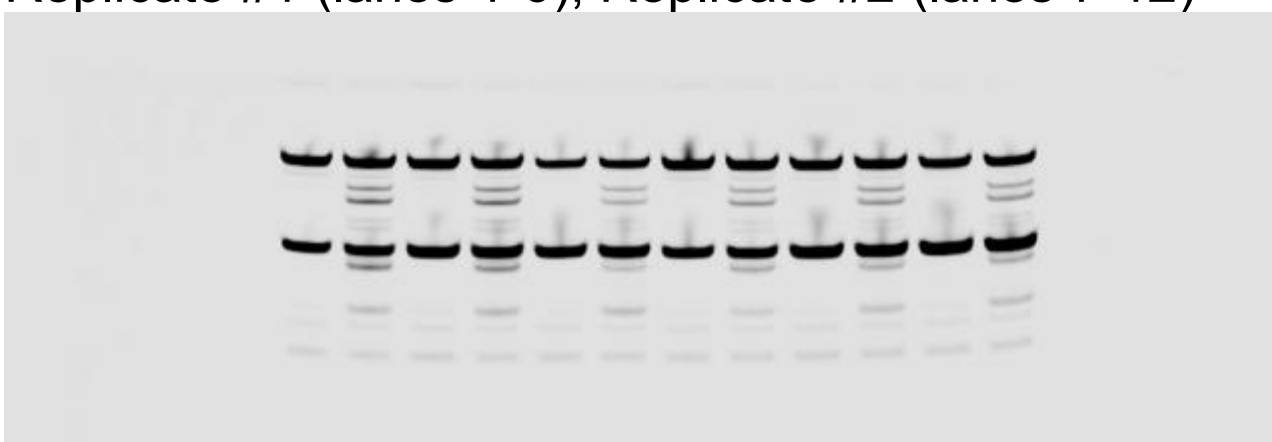

SI Figure 7b. Activity of Tz N15-1 in absence and presence of RNase H1.

Replicate #1 (lanes 1-6), Replicate #2 (lanes 7-12)

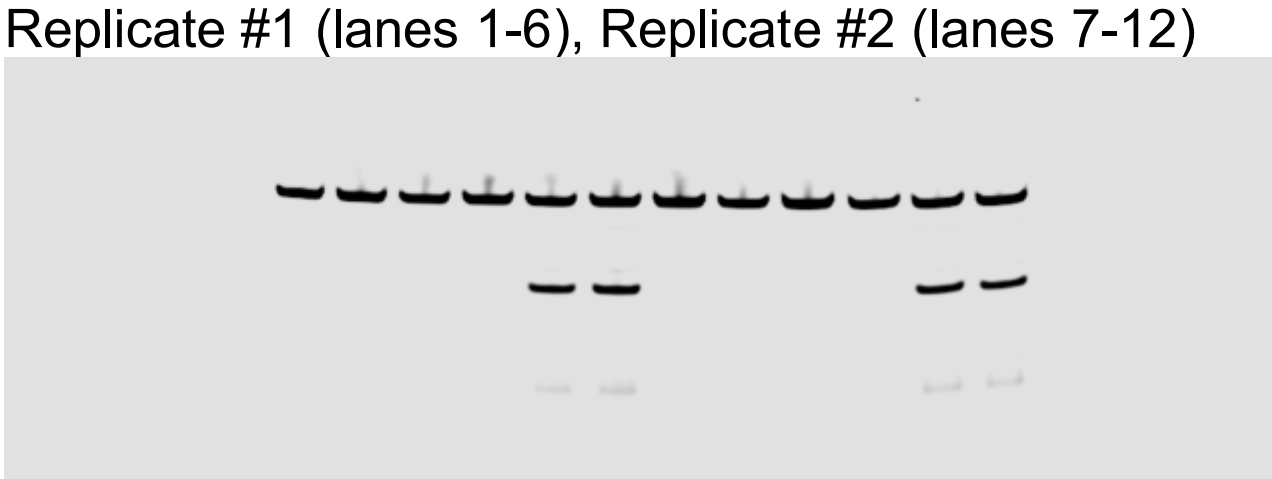

SI Figure 7c. Activity of Tz N20-1 in absence and presence of RNase H1.

Replicate #1 (lanes 1-6), Replicate #2 (lanes 7-12)

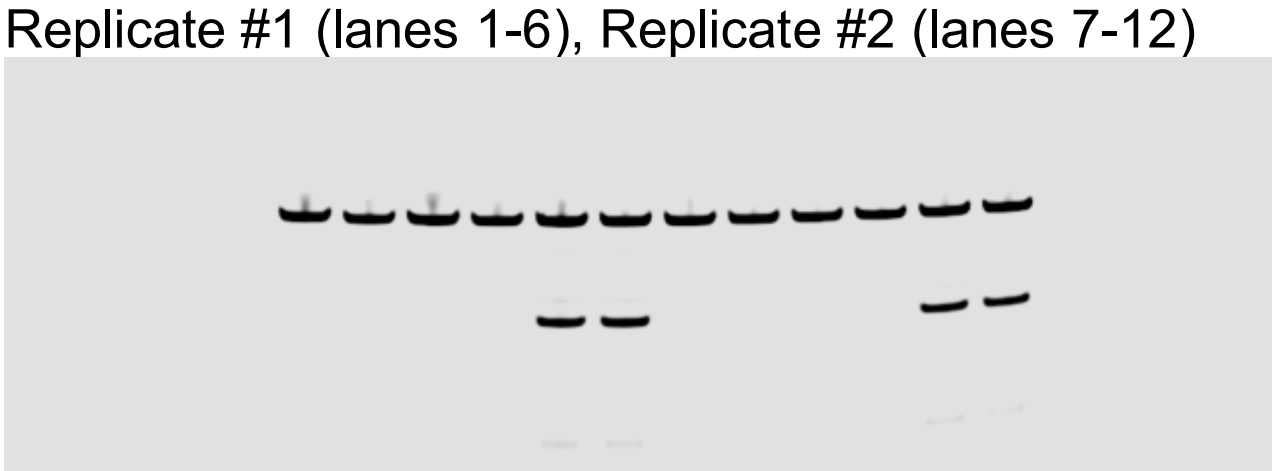

SI Figure 9b-c. Kinetic assays of Dz 10-23 (single turnover).

Replicate #1 (37˚C, top; 50˚C, bottom)

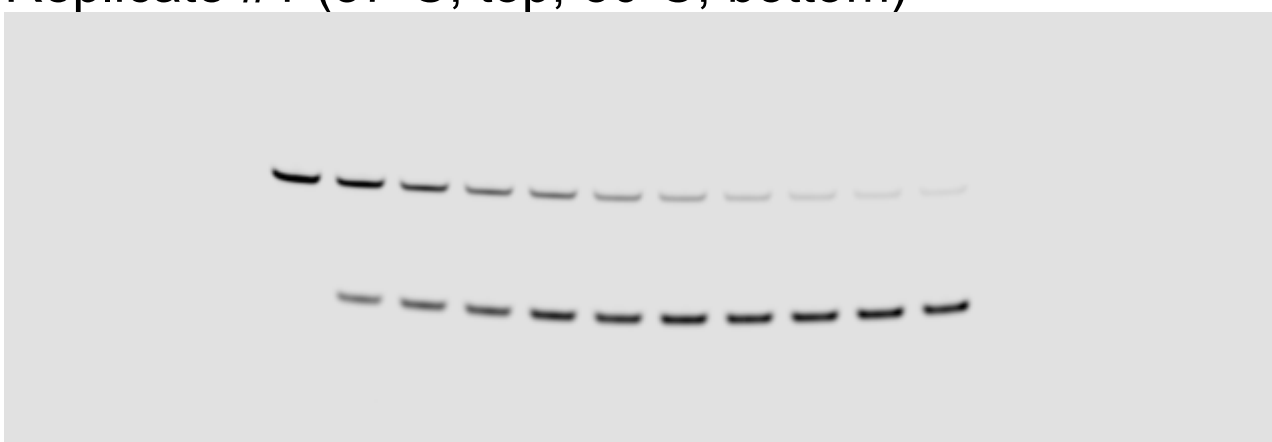

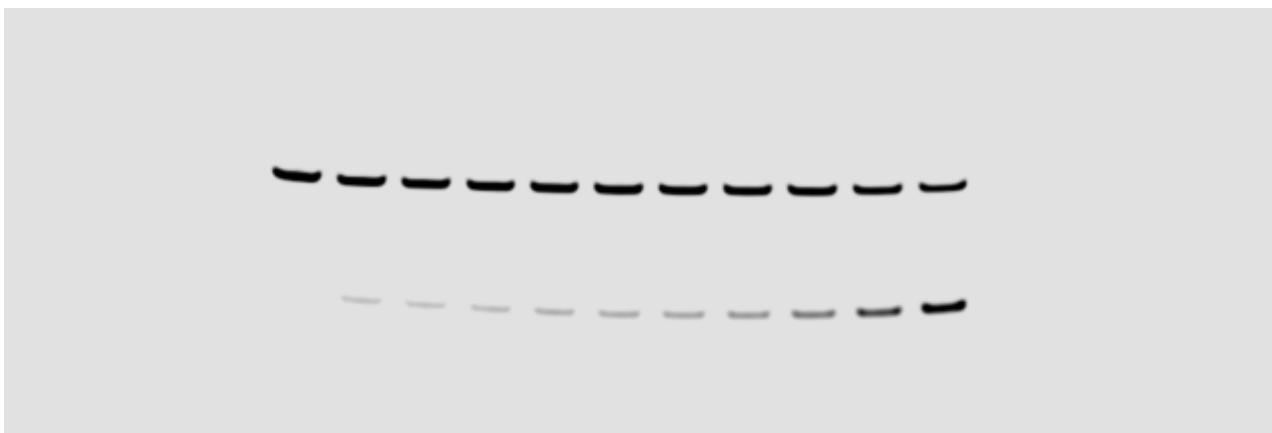

Replicate #2 (37˚C, top; 50˚C, bottom)

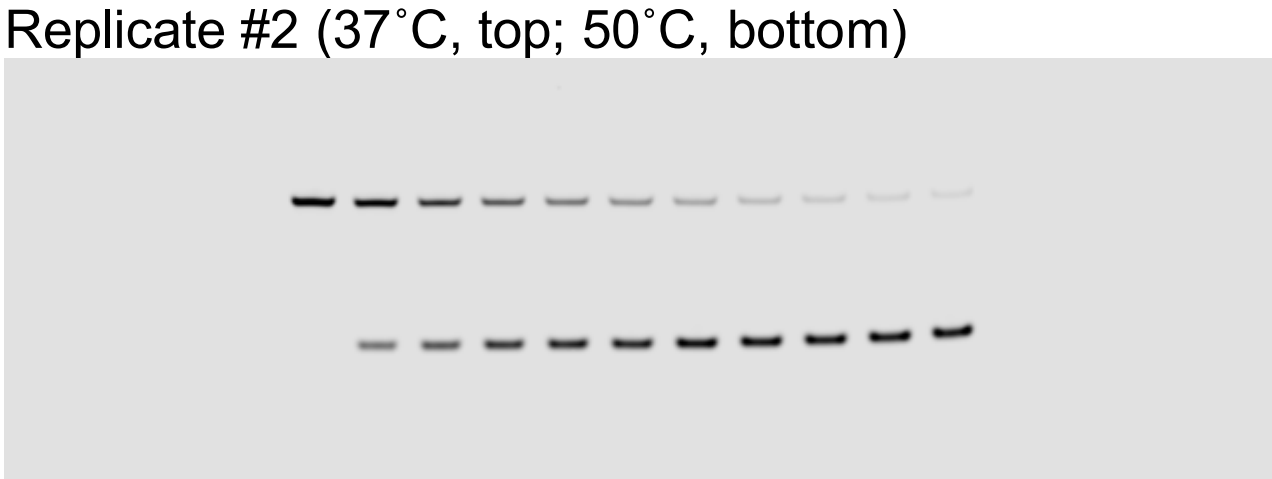

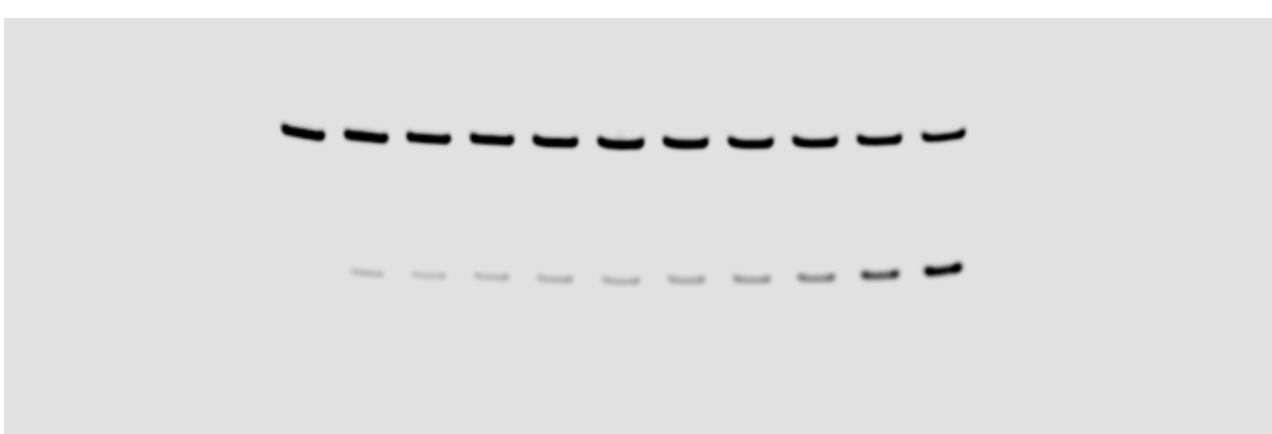

Replicate #3 (37˚C, top; 50˚C, bottom)

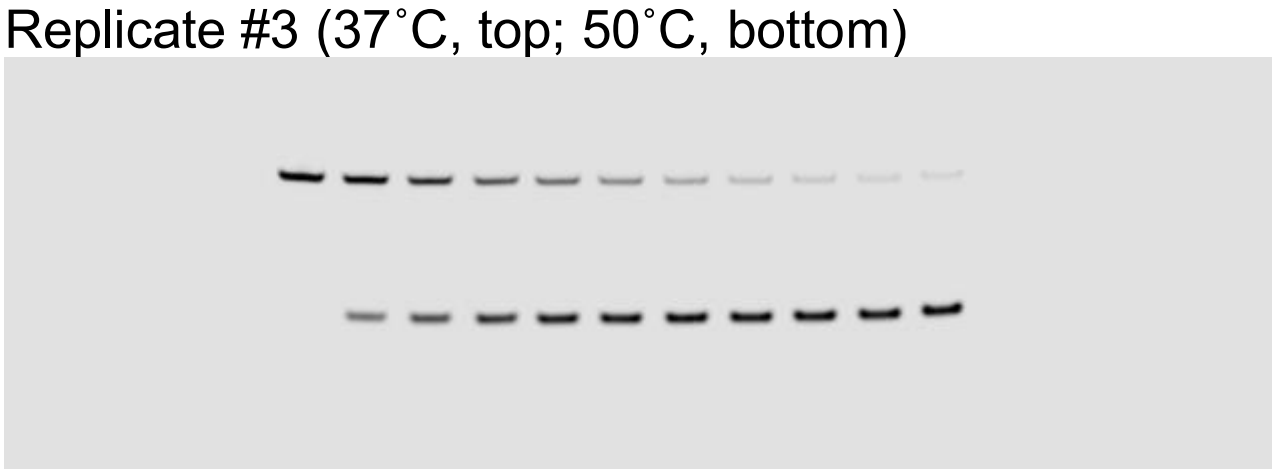

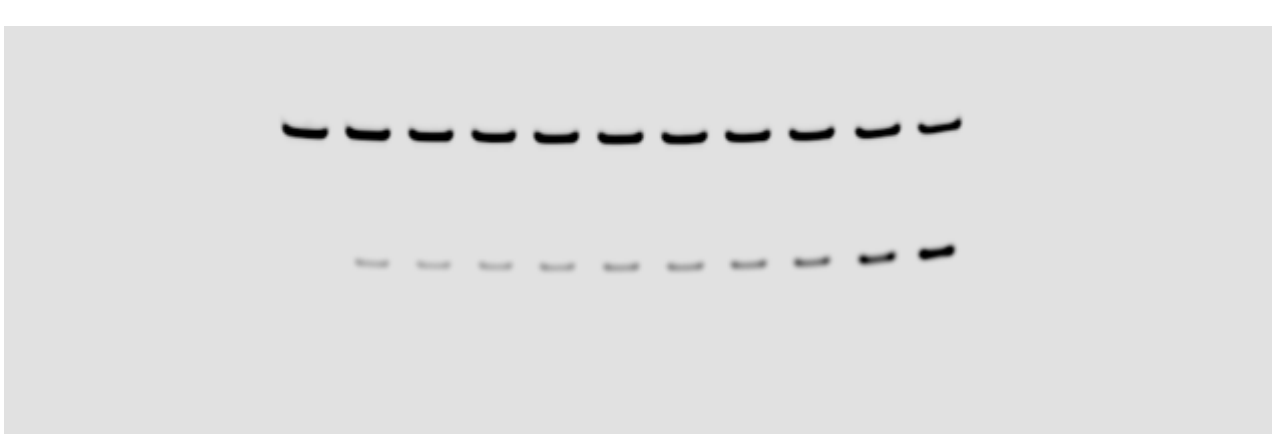

SI Figure 12a. Temperature optimum of RNA cleavage of Dz 10-23.

Replicate #1 (lanes 1-6), Replicate #2 (lanes 7-12)

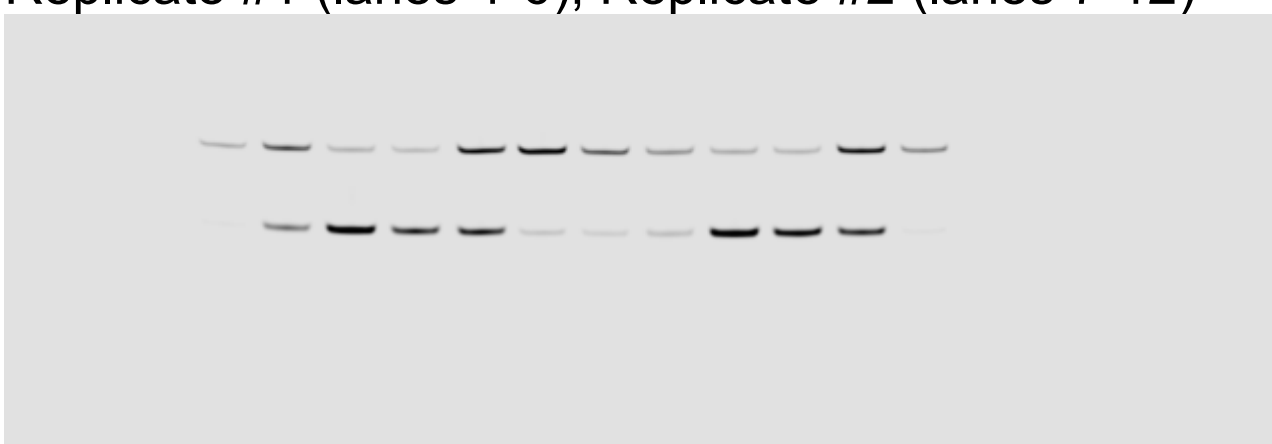

Replicate #3

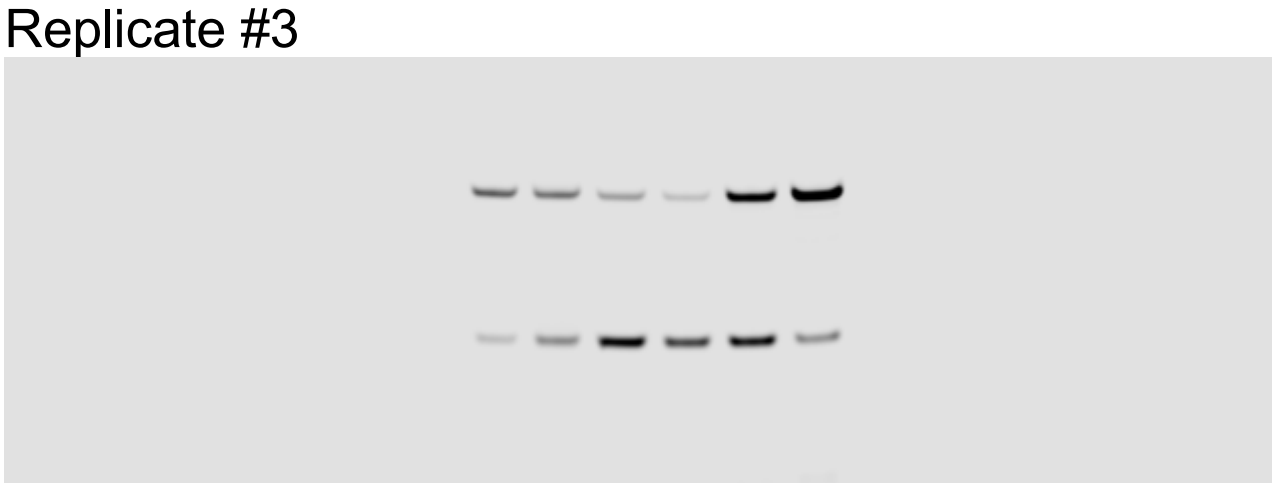

SI Figure 12b. Temperature optimum of RNA cleavage of Tz 6-29 and Tz 13-38.

Replicate #1

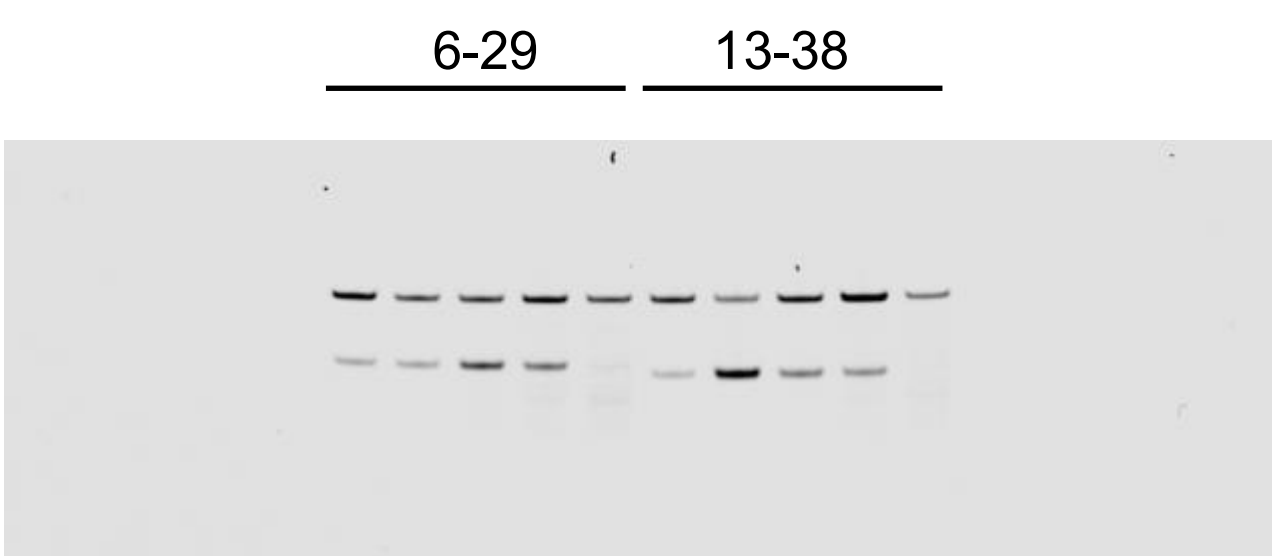

Replicate #2

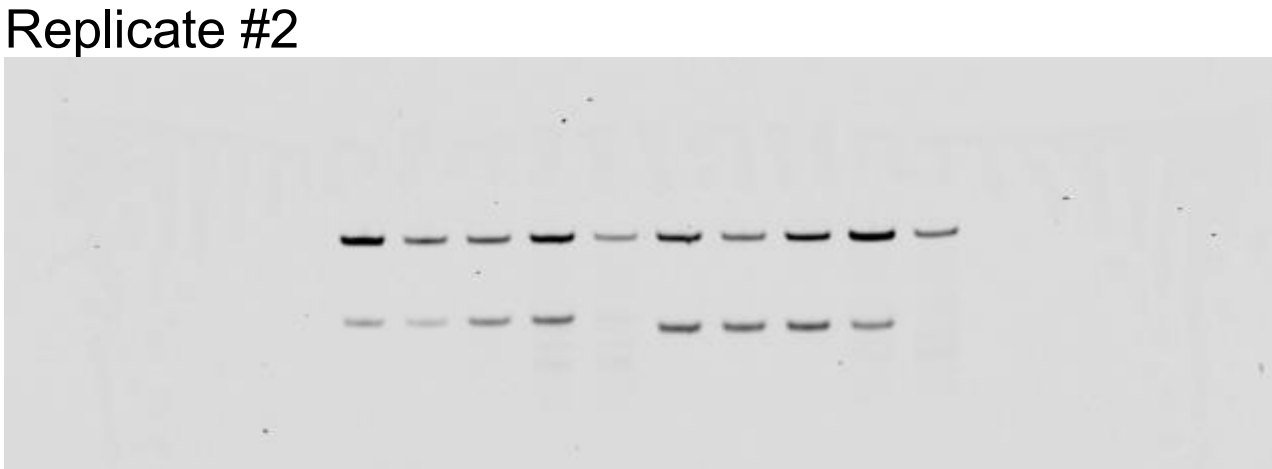

Replicate #3

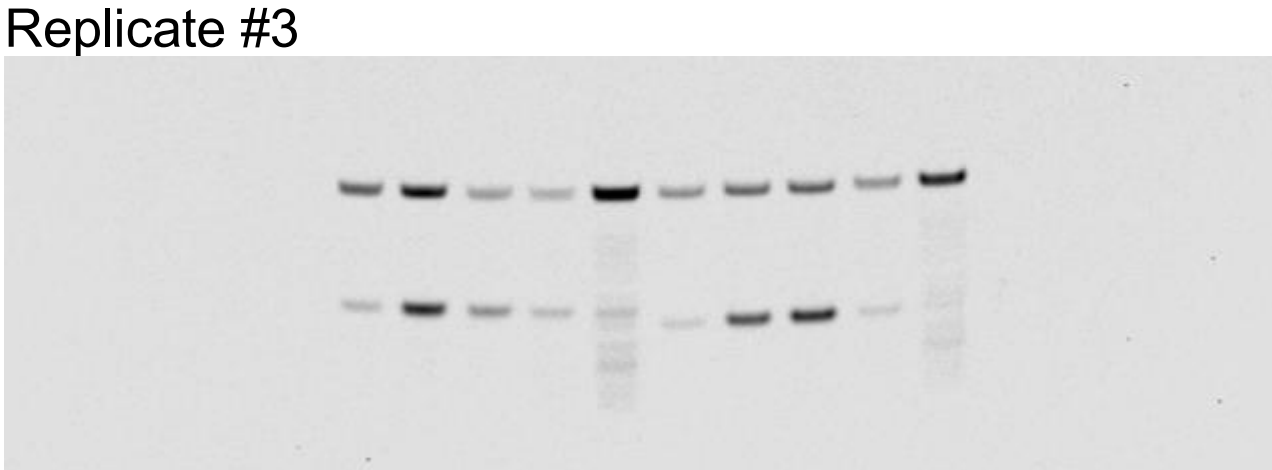

SI Figure 12c. Temperature optimum of RNA cleavage of Tz N15-1and Tz N20-1.

Replicate #1

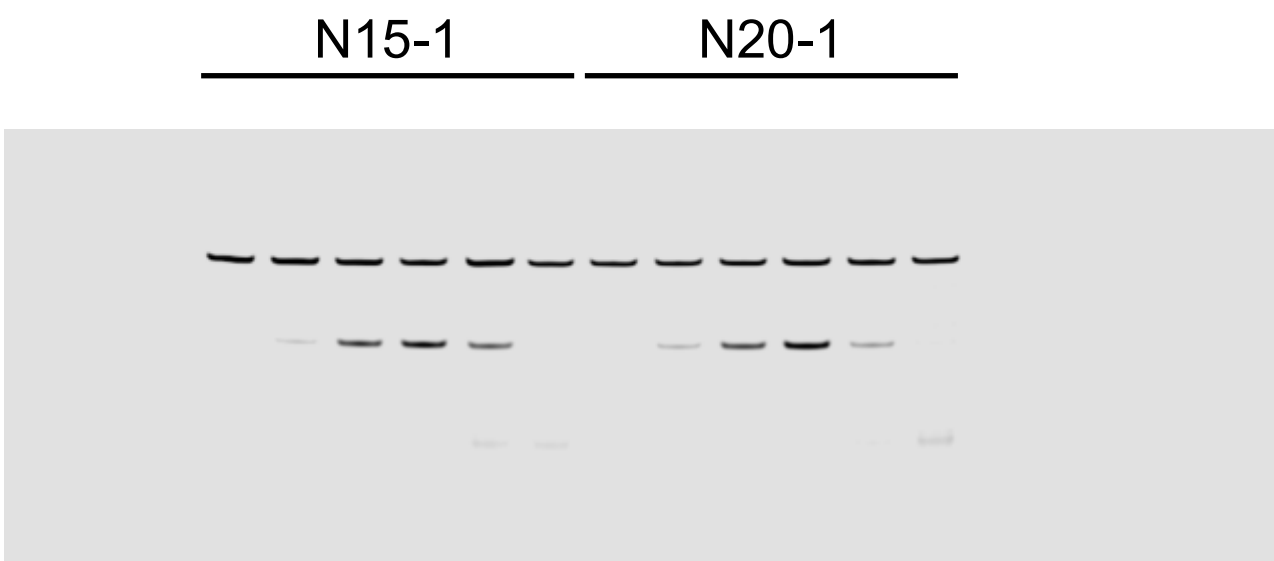

Replicate #2

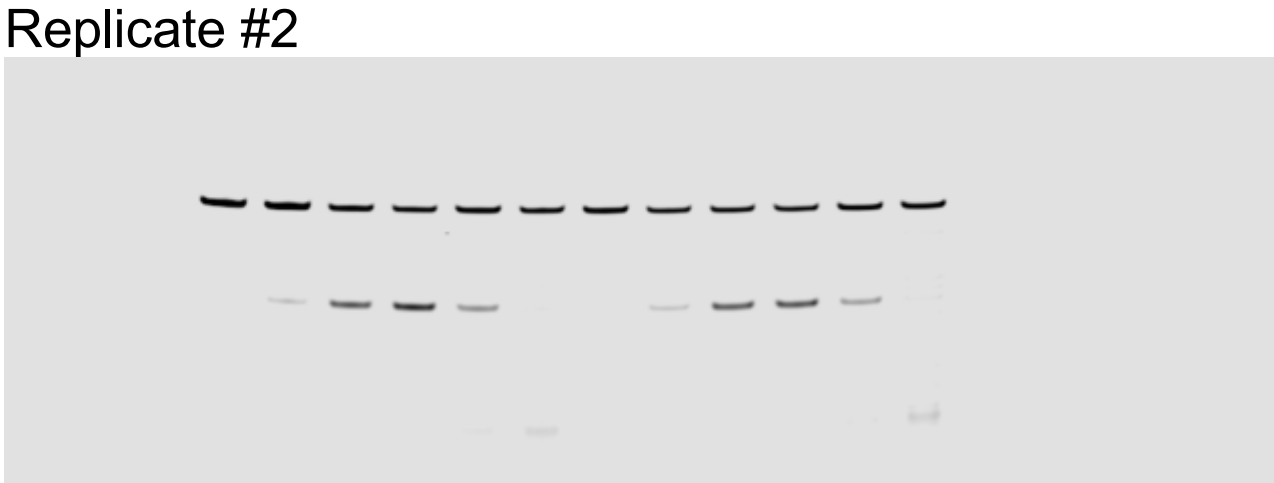

Replicate #3

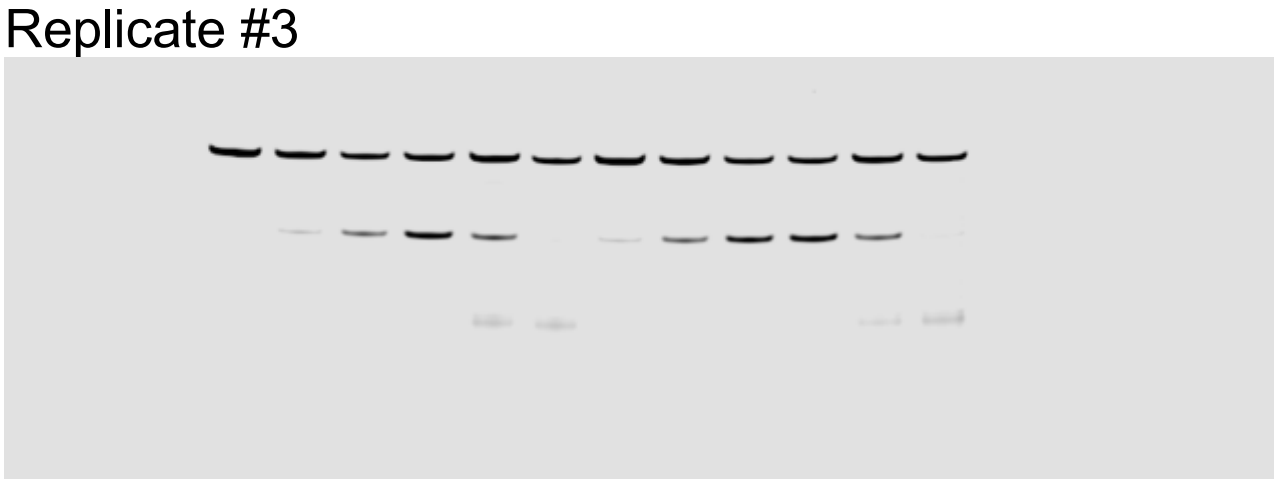

Supplement: Supplementary file 4 [file 41467_2026_76473_MOESM4_ESM.pdf]
